# MiR-662 is associated with metastatic relapse in early-stage breast cancer and promotes metastasis by stimulating cancer cell stemness

**DOI:** 10.1038/s41416-023-02340-9

**Published:** 2023-07-13

**Authors:** Margherita Puppo, Manoj Kumar Valluru, Martine Croset, Davide Ceresa, Michele Iuliani, Ashrin Khan, Julien Wicinski, Emmanuelle Charafe-Jauffret, Christophe Ginestier, Francesco Pantano, Penelope Dawn Ottewell, Philippe Clézardin

**Affiliations:** 1 Department of Oncology and Metabolism, Medical School, University of Sheffield, Sheffield, UK; 2 INSERM, Research Unit UMR_S1033, LyOS, Faculty of Medicine Lyon-Est, Lyon, France; 3 Univ Lyon, Université Claude Bernard Lyon 1, F-69008 Lyon, France; 4 Department of Infection, Immunity and Cardiovascular, Medical School, University of Sheffield, Sheffield, UK; 5 IRCCS AOU San Martino, Università degli studi di Genova, Genova, Italy; 6 Medical Oncology, Fondazione Policlinico Universitario Campus Bio-Medico, Via Alvaro del Portillo 200, 00128 Roma, Italy; 7 Department of Medicine and Surgery, Università Campus Bio-Medico di Roma, Via Alvaro del Portillo 21, 00128 Roma, Italy; 8 Aix-Marseille Univ, Inserm, CNRS, Institut Paoli-Calmettes, CRCM, Epithelial Stem Cells and Cancer Lab, “Equipe labellisée Ligue Contre le Cancer”, Marseille, France; 9 Present Address: INSERM U1052, CNRS UMR_5286, Centre Léon Bérard, Centre de Recherche en Cancérologie de Lyon, Lyon, France

**Keywords:** Breast cancer, miRNAs, Prognostic markers, Cancer stem cells

## Abstract

**Background:**

Breast cancer (BC) metastasis, which often occurs in bone, contributes substantially to mortality. MicroRNAs play a fundamental role in BC metastasis, although microRNA-regulated mechanisms driving metastasis progression remain poorly understood.

**Methods:**

MiRome analysis in serum from BC patients was performed by TaqMan™ low-density array. MiR-662 was overexpressed following MIMIC-transfection or lentivirus transduction. Animal models were used to investigate the role of miR-662 in BC (bone) metastasis. The effect of miR-662-overexpressing BC cell conditioned medium on osteoclastogenesis was investigated. ALDEFLUOR assays were performed to study BC stemness. RNA-sequencing transcriptomic analysis of miR-662-overexpressing BC cells was performed to evaluate gene expression changes.

**Results:**

High levels of hsa-miR-662 (miR-662) in serum from BC patients, at baseline (time of surgery), were associated with future recurrence in bone. At an early-stage of the metastatic disease, miR-662 could mask the presence of BC metastases in bone by inhibiting the differentiation of bone-resorbing osteoclasts. Nonetheless, metastatic miR-662-overexpressing BC cells then progressed as overt osteolytic metastases thanks to increased stem cell-like traits.

**Conclusions:**

MiR-662 is involved in BC metastasis progression, suggesting it may be used as a prognostic marker to identify BC patients at high risk of metastasis.

## Introduction

Breast cancer (BC) affects women worldwide, being the most common cancer in the UK with around 55,920 new cases and 11,547 deaths every year (Cancer Research UK, https://www.cancerresearchuk.org/, Last reviewed April 2023), the latter being essentially due to the metastatic progression of this disease [1, 2]. Metastatic BC occurs when cancer cells disseminate in distant organs—mainly bone—where they proliferate, generating secondary cancers that are often incurable. During metastasis, BC cells first evade from their primary site colonising surrounding areas, then intravasate in the systemic circulation (in vascular and lymphatic vessels), and finally extravasate to reach new microenvironments [1]. BC cells preferentially metastasise to the ‘fertile soil’ in bone where they survive and proliferate into overt metastases, although several years (even up to 10+ years) may be required before bone macro-metastases become detectable, posing a critical clinical problem [3]. So far, only a few biomarkers exist that have the potential to predict the risk of bone recurrence in BC patients, and there are no preventive targeted therapies that efficiently block the development of tumour growth in bone [1]. Moreover, although metastasis is a late event during cancer progression, it has been shown that the systemic spread of BC cells is an early event, with disseminated tumour cells being present in distant organs even when the primary tumour is not yet clinically detectable [1]. From a therapeutic point of view, it is essential to understand molecular mechanisms that allow BC cells to acquire a metastatic phenotype and develop tools to detect those changes at their early onset, even before the dissemination of cancer cells from a primary tumour to distant sites and their interaction with new microenvironments, such as bone, have occurred.

MicroRNAs (miRNAs) are small non-coding RNAs that are powerful and extremely versatile gene expression regulators within cells, playing an important role in every step of cancer progression [4]. A single miRNA can regulate thousands of transcripts at the same time; thus, an imbalance in the expression level of a single miRNA is able to produce a cascade of modifications in the transcriptome, allowing the transformation of normal cells into cancer cells [4, 5]. This explains why the dysregulation of miRNAs has profound effects in the acquisition of a malignant phenotype in BC cells, where proliferative, migratory, and invasive properties are enhanced to ultimately lead to BC metastasis [6, 7]. Interestingly, miRNAs are also essential regulators of cancer cell stemness, which is associated with a worse prognosis for BC patients due to an increased risk of metastasis recurrence [7]. Furthermore, miRNAs, conventionally described as circulating miRNAs, can be secreted from cancer cells –either contained within extracellular vesicles (EVs) or bounded to carrier proteins/lipoproteins– with the potential to reach the circulation [8]. Interestingly, EV-embedded miRNAs and Argonaute 2-bounded miRNAs have been shown to regulate gene expression of surrounding and distant cells, making the range of miRNA action tremendously wide [9]. The ability of miRNAs to be released from cells and circulate systemically can be used to monitor molecular changes that are happening within cells. Interestingly, circulating miRNAs are extremely stable in the serum, and can be detected with various techniques (e.g., PCR-based techniques) making them valid biomarkers with a high clinical potential to monitor disease progression [6]. Identifying primary tumour-derived circulating miRNAs, with a known role in metastasis progression, in the serum of cancer patients is also a critical information for the development of preventive therapeutic strategies to treat BC patients at high risk to develop metastases [5].

In the present study, we screened miRNA expression levels in the serum at baseline (time of surgery) from early-stage oestrogen receptor (ER)-positive (ER+) BC patients, and the data obtained were combined with long-term clinical follow-up information (>10 years) to identify circulating miRNAs associated with future recurrence in bone or other distant sites, compared to patients who did not experience relapse. Among these miRNAs, we identified hsa-miR-662 (here, abbreviated miR-662) as being associated with bone metastasis recurrence. Moreover, we found miR-662 as cargo of small EVs (sEVs) produced by BC cells. Of note, the role of miR-662 in BC and metastasis is currently unknown. So far, miR-662 has been found to be a poor prognosis factor in patients with early-stage squamous cell lung carcinoma (SCC) and to promote motility and chemoresistance of SCC cells in vitro [10]. Here, miR-662 functions in BC cells were investigated using several in vitro cell-based assays (proliferation, migration, cancer cell stemness, osteoclastogenesis), RNA sequencing, and metastatic outgrowth in bones of established animal models of BC metastasis [11]. Overall, our results show a pro-metastatic role for miR-662 in animal models, through enhancement of malignant traits (proliferation, migration, stemness) in BC cells.

## Materials and methods

### Human and murine cell lines

Human breast cancer lines (MDA-MB-231, T47-D, MCF7, BT-474, ZR-75-1, SK-BR3, Hs-578T) and human normal epithelial cell lines (MCF-10A, HMEC-1) were obtained from the ATCC and authenticated in-house by DNA fingerprinting, using short tandem repeat method of 10 loci. Stably firefly luciferase gene luc2-transfected MDA-MB-231 cell line, named MDA-MB-231-*luc2*-NW1 (NW1), was obtained from Dr Ning Wang, University of Sheffield, Sheffield, UK [12]. Murine triple-negative (TN) mammary 4T1*-luc2* cells were obtained from Dr. Amy Kwan, University of Sheffield, Sheffield, UK [13]. MDA-MB-231, NW1, T47-D, MCF7, BT-474, and 4T1*-luc2* cell lines were maintained in Dulbecco’s modified Eagle’s medium (DMEM) 4.5 g/L glucose GlutaMAX (Gibco, UK), ZR-75-1 in RPMI 1640 medium (Gibco, UK), SK-BR3 in McCoy’s 5A medium (Sigma), supplemented with 10% (v/v) FCS (Life Technologies, Paisley, UK /Invitrogen) and 100 U/mL penicillin/streptomycin, at 37 °C, 5% CO_2_. MCF-10A and HMEC-1 cells were cultured as previously described [14, 15]. Cell cultures were routinely tested for mycoplasma contamination (MycoAlert PLUS Mycoplasma Detection Kit, Lonza). Cell lines in culture were used within 20 passages after receipt.

### MiRome analysis by TaqMan™ low-density array (TLDA)

#### Serum collection and handling

Serum samples from Stage I, II and III BC patients were obtained from a large Phase 3 prospective trial with long-term clinical follow-up (>10 years) (Azure Trial; BIG 01/04), for which the subsequent metastasis status is known [16]. Serum samples were collected from treatment-naïve patients, at baseline, (time of surgery), aliquoted in 1-mL tubes, and stored at −80 °C under approved ethical conditions until assayed. The absence of serum haemolysis and fibrin was assessed by macroscopic visual analysis.

For this study, a discovery cohort of 48 serum samples were selected based on patients’ metastatic status and the subsequent treatment received after surgery (Table 1). According to the metastatic status, we divided the 48 patients into three groups: (1) patients with no metastatic recurrence at 10-year follow-up (NOMET, *n* = 16), (2) patients who first relapsed in bone (BONEMET, *n* = 16), and (3) patients who first relapsed in soft tissues (SOFTMET, *n* = 16). Moreover, in order to avoid any bias due to the effect of a bone-targeted therapy (zoledronic acid), patients who received this treatment at baseline were equally randomised between the three groups (43.7–62.5% of cases per group; Table 1).

**Table 1:** Clinical details on patients’ cohort.

Characteristics	NOMET *n (%)*	SOFTMET *n (%)*	BONEMET *n (%)*	Total
Sample size (*n*)	16	16	16	48
Age (range in years)	53–76	51–75	54–73	51–76
Menopausal status	More than 5 years since menopause (100)			48
Lymph node involvement	Positive (100)			48
ER status	Oestrogen receptor positive (100)			48
Tumour grade				
I	4 (25)	0 (0)	0 (0)	4
II	7 (43.7)	7 (43.7)	4 (25)	18
III	5 (31.3)	9 (56.3)	12 (75)	26
Tumour stage				
T1	3 (18.7)	7 (43.7)	5 (31.2)	15
T2	12 (75)	8 (50)	3 (18.7)	23
T3	1 (6.3)	1 (6.3)	6 (37.5)	8
T4	0 (0)	0 (0)	2 (12.6)	2
Zoledronic acid treatment	7 (43.7)	10 (62.5)	9 (56.3)	26

In total, 48 early-stage BC patients that belong to the AZURE cohort have been selected for the scope of this study. Clinical-biological characteristics are here displayed alongside relative percentages.

#### RNA isolation from serum

Total RNA was extracted from 200 µL serum using the miRCURY Biofluids RNA Isolation Kit (Qiagen) according to the manufacturer’s recommendations. Briefly, serum samples were thawed on ice, centrifuged, and a lysis buffer solution containing glycogen and ath-miR159a as synthetic spike-in control RNA (Life Technology) added. After 3 min incubation at RT, a protein precipitation solution was added, samples were centrifuged, the clear supernatant was collected, mixed 1:1 with isopropanol, loaded in a silica micro spin-column, washed twice, and further centrifuged. An on-column DNase digestion was performed by adding 50 µL of rDNase onto the membrane for 15 min at RT. After washes, total RNA was eluted with 45 µL RNase/DNase-free water (Invitrogen), and immediately stored at −80 °C until assayed.

#### MiRNA expression profile by TLDA

For each serum sample, 10 μg of total RNA was retro-transcribed in cDNA by two consequential RT reactions using the TaqMan MicroRNA Reverse Transcription Kit and the Megaplex™ RT Human Pool A, Pool B (Applied Biosystems) according to the manufacturer’s recommendations. Briefly, a master mix containing 10× RT Buffer, 100 mM dNTP mix, 20 U/µL RNase Inhibitor, 50 U/µL MultiScribe RT, and 25 mM MgCl_2_, was added to the total RNA for a final volume of 20 µL. 10X Megaplex™ RT Human Pool A v2.1 was added allowing the reverse transcription of a first set of miRNAs; in a second reaction mixture, 10× Megaplex™ RT Human Pool B v3.0 was added allowing the reverse transcription of a second set of miRNAs. The thermal cycler program used was: 16 °C for 2 min, 42 °C for 1 min, 50 °C for 1 s (×40 cycles), followed by 5 min at 85 °C. MiRNA quantification was improved by a cDNA pre-amplification step using the Megaplex™ PreAmp Primers. The cDNA was added to 2× Single Cell PreAmp mix containing the 10× Megaplex™ PreAmp Primers Card A v2.1 and Card B v3.0. The reaction conditions were: 95 °C for 10 min, 55 °C for 2 min, 72 °C for 2 min, followed by 18 cycles of: 95 °C for 15 s, 60 °C for 4 min, and a final step at 99 °C for 10 min. Two preloaded 384-well TaqMan microfluidic cards (A, B) were used to analyse 646 human miRNAs (Supplementary Table S1). Amplified cDNAs were mixed with 2× TaqMan Universal PCR Master Mix, No AmpErase UNG, and nuclease-free water. 100 µL of each sample were added to the eight fill ports of the array and a QuantStudio® 7 flex real-time qPCR system (Applied Biosystems) was used for cDNA amplification. The thermal cycling parameters used were: 95 °C for 10 min, followed by 95 °C 1 s and 60 °C 20 s (×40 cycles). CTs were recorded as cycle numbers at which the fluorescence generated within the reaction crosses the fluorescence threshold significantly above the ROX™ fluorescence background recorded in each sample.

### Receiver operating characteristic (ROC) curves analysis

ROC curves were constructed to evaluate the value of circulating miRNAs in distinguishing between groups [MET (BONEMET + SOFTMET) vs NOMET, BONEMET vs NOMET, SOFTMET vs NOMET]. Areas under the ROC curves (AUC) were calculated based on 2^-ΔCT^ values.

### MiRNA overexpression in cancer cells

#### Transient miRNA overexpression

2’O-methylated miRNA mimics for hsa-miR-662 (MIMIC-miR-662), negative control (MIMIC-negCTRL), and FAM-labelled negative control (MIMIC-negCTRL-FAM+), were purchased from GenePharma, Shanghai. An optimised concentration of 50 nM of miRNA mimic was transfected into cells (MDA-MB-231, NW1, 4T1*-luc2*), using Lipofectamine 2000 (Invitrogen) as transfecting agent (Supplementary Fig. S1). Evaluation of transfection efficiency has been routinely performed by flow-cytometry (BD LSR II Flow Cytometer) followed analysis with Flowing Software 2.5.1 (Perttu Terho, University of Turku), and by real-time qPCR, using specific primers (MIMIC-negCTRL: 5’-TTCTCCGAACGTGTCACGTTT-3’; MIMIC-miR-662: 5’-TCCCACGTTGTGGCCCAGCAG-3’). U6 and SNO234 have been used as housekeeping genes (U6: 5’-TTCGTGAAGCGTTCCATATTTTT-3’; SNO234: 5’-TTCGTCACTACCACTGAGA-3’). All in vitro experiments conducted with MIMIC-transfected cells were performed between 24 h and 6 days based on the length of MIMIC-miR-662 overexpression (> ten fold after 8 days, Supplementary Fig. S1). All in vivo experiments conducted with MIMIC-transfected cells were performed between 8 and 10 days taking in consideration that non-proliferative, circulating cancer cells in blood are likely to conserve miRNA-mimics for a longer period of time.

#### ShMIMIC lentiviral microRNA particles transduction

Lentivirus vectors (shMIMIC human lentiviral microRNA hsa-miR-662 hCMV-TurboGFP, SMARTvector non-targeting hCMV-TurboGFP control particles) were purchased from Horizon Discovery LTD (Cambridge, UK). NW1 cell infections were carried out at a MOI (multiplicity of infection) of 20, and the culture medium was changed 6 h post-transduction. Transduced cells (NW1/LENTI-662-GFP + , NW1/LENTI-Ctrl-GFP + ) were further selected for 2 weeks with 8 μg/mL puromycin.

### Real-time quantitative PCR (qPCR)

Cultured cells were collected at 80% confluency. Total RNA, enriched for miRNAs, was extracted using a miRNeasy kit (Qiagen) and manufacturers’ instructions. RNA concentration and purity were evaluated using a Nanodrop™ 2000 spectrophotometer (Thermo Scientific). RNA was reverse transcribed into cDNA using miScript II RT Kit (Qiagen) on a Peltier Thermocycler-200 (MJ Research). Real-time qPCR was performed using a C1000 Touch Thermal Cycler (Bio-Rad) and miScript SYBR® Green PCR Kit (Qiagen). Gene expression was assessed by analysing the number of cycles (CTs) of genes (*hsa-miR-662, Notch1*, *Wnt7b*, *Zeb1*, *Tcf3*, *Ctnnb1*, *Cd44*, *Cd24*, *Snai2, Oct-04*, *c-Myc*, *Ehz2*) in comparison to housekeeping genes (*U6*, *SNO234* for miRNAs; *B2M* for mRNAs) to obtain delta CT values (ΔCts). Relative expression levels are expressed as 2^-ΔCT^.

### Small extracellular vesicle (sEV) isolation by ultracentrifugation

Cells (NW1, NW1/LENTI-Ctrl-GFP + , NW1/LENTI-662-GFP + , SK-BR3) were seeded at 20% of confluency in their respective complete medium. After 24 h, media were replaced with sEV-free media (sEV-deprived media by ultracentrifugation), and cells were kept in culture for an additional 72 h. Then, conditioned media were collected, and centrifuged at 300×*g* for 10 min at 4 °C to remove cell debris. A second centrifugation at 10.000×*g* for 10 min at 4 °C has been conducted to eliminate large EVs, followed by an ultracentrifugation at 100.000×*g* for 70 min at 4 °C (Optima XPN-80 Beckman Coulter Ultracentrifuge). sEV pellet has been then resuspended in QIAzol (Qiagen) for small-RNA extraction as previously described.

### ALDEFLUOR assay

ALDH activity experiments were conducted as previously described [17], using the ALDEFLUOR kit (StemCell Technologies, Durham, NC, USA). Briefly, for breast cancer patient-derived xenograft (PDX) models, a single-cell suspension from freshly dissociated tumours was suspended in ALDEFLUOR assay buffer and incubated with the ALDH substrate (BAAA, 1 μmol/L per 1 × 10^6^ cells) for 40 min at 37 °C. As a negative control, each sample was additionally treated with 50 mmol/L of an ALDH inhibitor, diethylamino-benzaldehyde (DEAB). Murine cells were excluded following staining with an anti-H2Kd antibody (BD biosciences, 1/200, 20 min on ice), followed by a secondary antibody labelled with phycoerythrin (PE) (Jackson labs, 1/250, 20 min on ice). For BC cell lines, a single-cell suspension was suspended in ALDEFLUOR assay buffer and incubated with BAAA (1 μmol/L per 3 × 10^5^ cells) for 30 min at 37 °C. Sorting gates were established using ALDEFLUOR-stained, DEAB-treated cells as a negative control (Supplementary Fig. S2).

### Tumour sphere assay

Transfected cells (NW1/MIMIC-miR-662, NW1, MIMIC-negCTRL) were seeded in wells (2 × 10^3^ cells/well) of non-adherent round bottom 96-well plates (Nunclon Sphera, Thermo Scientific) where 33 µL/well of Matrigel (Thermo Scientific) in complete medium were previously added. Plates were centrifuged for 10 min at 4 °C prior to being placed in the incubator. Tumour spheres were monitored over time and collected at day 7 to perform ALDH activity experiments.

### Cell proliferation assay

Twenty-four hours post-transfection, MIMIC-transfected MDA-MB-231 cells (MDA-MB-231/MIMIC-miR-662, MDA-MB-231/MIMIC-negCTRL-FAM + ) or stably miRNA-overexpressing cells (NW1/LENTI-Ctrl-GFP + , NW1/LENTI-662-GFP + ) were seeded 24 h post-transfection into 12-well plates (Costar) in triplicate at a concentration of 1 × 10^4^ cells/500 μL/well. At day 0, 1, 2, 3, and 4, tumour cells were washed with PBS, fixed with 4% (v/v) paraformaldehyde (PFA; Fisher Scientific, UK), stained with crystal violet (Sigma-Aldrich), solubilised with a solution 10% (v/v) acetic acid, and the optical density (590 nm) was measured using a SpectraMax M5e microplate reader (Molecular Devices).

### Transwell cell migration assay

Twenty-four hours post-transfection, MIMIC-transfected MDA-MB-231 cells (MDA-MB-231/MIMIC-miR-662, MDA-MB-231/MIMIC-negCTRL-FAM + ) or stably miRNA-overexpressing cells (NW1/LENTI-Ctrl-GFP + , NW1/LENTI-662-GFP + ) at 80% confluence were treated with 10 μg/mL of mitomycin C (Sigma) to prevent proliferation prior performing the cell migration assay. Tumour cells were then resuspended in RPMI 1640 serum-free medium containing 0.1% (w/v) BSA, and seeded in the upper chamber (8-μm diameter pore-size inserts) of 24-well transwell migration plates (10^4^ cells/insert; Costar), while the chemoattractant [10% (v/v) FBS-containing DMEM] was placed in the lower chamber. After 24 h at 37 °C, cells in the upper chamber were removed with a cotton swab, and cells that had migrated through the porous membrane were fixed in 100% ethanol, stained with crystal violet, imaged under a Leica RMRB upright microscope, and analysed with ImageJ 1.53k, Java 1.8.9_172 (64-bit) software.

### Osteoclastogenesis assays

#### Conditioned medium collection

For MIMIC-miR-662 or MIMIC-negCTRL transfected NW1 cells, culture medium was replaced after 6 h from transfection with new serum-free DMEM medium, collected after 24 h, centrifuged to remove cells/cell-debris, aliquoted, and stored at −80 °C until further use. For NW1/LENTI-662 and NW1/LENTI-neg cells, once cells reached 70–80% confluency, culture medium was replaced with new serum-free DMEM medium, collected after 24 h, centrifuged, aliquoted, and stored at −80 °C until further use for osteoclastogenesis assays.

#### Murine osteoclastogenesis assay

Experiments with murine-derived osteoclasts were conducted as previously described [18], with minor modifications. Briefly, bone marrow cells from tibiae and femora of 6/8-week-old OF1 male mice (Charles River, Kent, UK) were flushed, centrifuged, resuspended in Ficoll® Paque Plus (Cytiva), and further centrifuged to allow the isolation and enrichment of mononuclear cells. Overall, 1 × 10^5^ cells from the isolated mononuclear cell fraction were then seeded in 12-well plate wells, and cultured 24 h in α-MEM medium containing 20% (v/v) FCS (Invitrogen) with 20 ng/mL of macrophage colony-stimulating factor (M-CSF; R&D Systems). The next day, culture medium was replaced with a differentiation MEM-α medium containing 10% (v/v) FCS, 20 ng/mL M-CSF, and 10 ng/mL of soluble recombinant receptor activator of nuclear factor κB ligand (RANKL; R&D Systems), and kept in culture for 7 days. Cells were continuously (day 1–7) exposed to the conditioned medium from transfected tumour cells (1:16 dilution). At day 7, mature multinucleated osteoclasts were fixed, stained for TRAP activity (Sigma-Aldrich), and counted as osteoclasts when having 3 or more nuclei. In addition, the total area covered by osteoclasts was measured using ImageJ.

#### Human osteoclastogenesis assay

Primary human osteoclasts were differentiated from human peripheral blood mononuclear cells of healthy donors as previously described [19, 20]. Briefly, 1 × 10^4^ CD14+ monocytes resuspended in RPMI medium containing 10% (v/v) FCS, M-CSF (25 ng/mL) and RANKL (50 ng/mL) were seeded in 96-well plates. After 72 h in a 5% CO_2_ incubator, cell culture medium was replaced with fresh medium containing 10% (v/v) foetal calf serum, M-CSF, and RANKL with or without conditioned medium from cancer cells (1:16 dilution). This medium was changed every 3 days. At day 12, cells were fixed and stained for TRAP activity. Mature multinucleated osteoclasts (>3 nuclei) were enumerated, and the total area covered by osteoclasts measured using a Nikon NIS-Elements microscope imaging software.

### High throughput sequencing and RNA-seq bioinformatic analysis

Total RNA of three independent samples was extracted from efficiently (>90%) transiently transfected NW1 (NW1/MIMIC-miR-662, NW1/MIMIC-negCTRL-FAM + ) cells using the RNeasy Mini Kit (Qiagen), according to the manufacturer’s recommendations. Cell collections were performed 36 h post-transfection based on previous published work [21]. The optional on-column DNase digestion (15 min, room temperature) was performed using RNase-free DNase I Kit (Qiagen). After washing, total RNA was eluted with 30 µL RNase/DNase-free water, and immediately stored at -80 °C until assayed. Total RNA quantity and purity (all samples, RIN ≥ 9.6) were evaluated with a 2100 Bioanalyzer (Agilent) and a Qubit RNA IQ Assay (ThermoFisher), respectively. Total RNA (675 ng) was used for RNA-seq library preparation with Poly-A enrichment, and single-end sequencing was undertaken on the Illumina HiSeq™ 2500 platform in rapid run mode using the Illumina HiSeq™ Rapid Cluster Kit (Illumina, Inc., San Diego, CA, USA). RNA-seq data was aligned to GRCh38 human genome assembly using STAR v2.7.5c (Dobin and Gingeras, [22]). Transcript quantification was performed using RSEM v1.3.1 (Li and Dewey, [23]), and poorly expressed transcripts (less than 0.5 counts per million in all samples) were eliminated for further analysis. Counts were normalized by weighted trimmed mean of *M*-values [24] using TMM function of EdgeR Bioconductor package [25]. Differential gene expression (DE) analysis, based on negative binomial generalized linear models, was performed using EdgeR Bioconductor package to compare mimic miRNA-662 transfected cells to mimic negative control cells. Further gene set enrichment analysis (GSEA) [26] was performed using ClusterProfiler Bioconductor package [27] on genes ranked by the fold change estimated in DE analysis. A manual annotation of gene networks has been performed based on the description of each gene network retrieved from GSEA website (https://www.gsea-msigdb.org/gsea/index.jsp, Accessed May, 2022).

### TargetScan target prediction and ClueGo Analysis

TargetScanHuman 7.0 software (https://www.targetscan.org/vert_70/, Accessed April, 2021) was used to predict hsa-miR-662 targets in human transcriptome by searching for the presence of 8mer, 7mer, and 6mer sites that matched with miR-662 seed region. Top 200 predicted targets (arbitrary threshold) were used to perform a ClueGo-based analysis [28] entering ‘GO Biological Process’, ‘GO molecular functions’, ‘KEGG’, ‘Reac Pathways’ and ‘Reac Reactions’ as databases.

### Animal studies

Animal experiments conducted at the University of Sheffield (UK) were performed using young (6-to-7-week-old) female BALB/c ^fox/-^ *nude* or BALB/c mice (Charles River, Kent, UK) kept on a 12-h/12-h light/dark cycle with free access to food and water. Animal studies were carried out in accordance with local guidelines and UK Home Office approval under project licenses 70/8964 and P99922A2E, University of Sheffield, UK.

For the long-term experiment in immunodeficient mouse models, NW1 cells stably overexpressing miR-662 (NW1/LENTI-662-GFP + ) or control cells (NW1/LENTI-Ctrl-GFP + ) were injected into the left cardiac ventricle (2.5 × 10^4^ cells) of BALB/c ^*fox/-*^ *nude* mice. Mice were sacrificed when reaching their humane endpoint at a maximum of 7 weeks post-tumour cell injection. Serum was collected, aliquoted, and stored at −80 °C for downstream analysis by ELISA; tibiae were fixed in 4% (v/v) paraformaldehyde (PFA; Fisher Scientific, UK), decalcified with 0.5 M EDTA, and processed for histology.

For the short-term experiment in an immunodeficient mouse model, NW1 cells transiently transfected with MIMIC-miR-662 (NW1/MIMIC-miR-662) or negative control (NW1/MIMIC-negCTRL-FAM + ) cells were injected into the left cardiac ventricle (5 × 10^4^ cells) of BALB/c ^*fox/-*^ *nude* mice 72 h post-transfection. Mice were sacrificed 9 days after intracardiac tumour cell injection. Serum was collected as described above.

For the experiment in a syngeneic animal model, 4T1-*luc2* cells transiently transfected with MIMIC-miR-662 (4T1/MIMIC-miR-662) or negative control (4T1/MIMIC-negCTRL-FAM + ) were injected into the left cardiac ventricle (5 × 10^4^ cells) of BALB/c mice 72 h post-transfection. Mice were sacrificed 10 days after intracardiac tumour inoculation. Serum and tibiae were collected as described above.

For patient-derived xenograft (PDX) model experiments, animal studies were conducted at the Aix-Marseille University (France) in agreement with French Guidelines for animal handling and approved by local ethics committee (Agreement no. 16487‐2018082108541206, v3). Animals were kept on a 12-h/12-h light/dark cycle with free access to food and water. ER-positive or TN breast tumours (*N* = 6) were implanted in the fat pad of NOD/SCID immunodeficient mice, and collected when reaching 1 cm diameter, as previously described [7, 29].

### Bioluminescence and microcomputed tomography (micro-CT) imaging

Experiments were conducted as previously described [11, 30]. Briefly, tumour growth was monitored in anesthetised mice using an IVIS Lumina II system (Caliper Life Sciences, UK), following subcutaneous injection of d-Luciferin (Invitrogen, UK) to animals 4 min before imaging. At autopsy, additional ex vivo imaging was performed on hindlimbs and major organs. Micro-CT analysis was carried out on fixed tibiae using a Skyscan 1172 X-ray–computed microtomography scanner (Aartselaar, Belgium). Pixel size was set to 4.3 mm, and scanning initiated from the top of the proximal tibia.

### Bone histology

Histological analysis was carried out on paraffin-embedded medial sections of tibial metaphysis as previously described [11, 30]. The in situ detection of osteoclasts was performed on tartrate-resistant acid phosphatase (TRAP)-stained bone tissue sections using a commercial kit (Sigma). Osteoclast numbers per millimeter (mm) in the cortical-endosteal and trabecular bone surfaces were blind counted and analysed following University of Liverpool’s [31]. The presence of metastasis was assessed by haematoxylin and eosin (H&E) staining of bone tissue sections.

### Enzyme-linked immunosorbent assays (ELISA)

Serum concentrations of bone resorption (tartrate-resistant acid phosphatase-5b, TRAcP 5b) and formation (pro-collagen type I N propeptide, P1NP) markers were measured by ELISA using commercially available kits (MBS727192, MyBioSource; E-EL-M3033, Elabscience), following manufacturers’ instructions.

### Statistical analysis

For clinical data, to determine the sample size for the discovery patients’ cohort (*N* = 48), we evaluated that with a sample size of 16 for each group (MET, BONEMET, SOFTMET) for which the effect size d was set at 1.3 −/+ (α err prob = 0.01), we achieved a power (1-β err prob) of 0.815 by a two-tails post hoc compute achieved power analysis. For ROC curve analysis, statistical significance was set at *P* < 0.05 (two-sided test). We implemented AUC > .65 (*P* < 0.05) cut-off for generating dot plot or heatmap using Broad institute software Morpheus. For combined ROC curves, a binary logistic regression was calculated using IBM SPSS Statistics. Correlations of expression between the different miRNAs were performed by Spearman’s or Pearson’s correlation test. Intervene’s UpSet module was used to visualize the intersection of multiple cohort sets using UpSet plots (https://bitbucket.org/CBGR/intervene). Survival analysis was performed dividing patients into two groups [high (value > 0.1) and low (value <−0.1)] based on miR-662 expression (Z-score of 2^-ΔCT^). The Kaplan–Meier estimator was used to determine the relationship between miRNA expression and patient survival. Differences in survival across the strata were calculated using a log-rank *P* test.

For in vitro experiments, all statistical analyses on experimental data were performed using Prism GraphPad 9.2.0 (GraphPad Software Inc., San Diego, CA, USA). Statistical significance was measured using parametric testing (*t* test or ANOVA), assuming equal variance, or non-parametric testing (Fisher’s exact test). ANOVA was followed by Tukey’s multiple comparison testing. The ANCOVA analysis of co-variance was performed to evaluate multiple regression for metastatic progression. ROUT method (Q = 1%) was used to identify outliers (Prism GraphPad 9.2.0). For all tests, statistical significance was defined as *P* value (*P*) ≤0.05. All graphs represent mean ± standard error mean (SEM), *P < 0.05, **P < 0.01, ***P < 0.001, ****P < 0.0001. All in vitro experiments consisted of at least three independent biological repeats with appropriate controls.

For in vivo experiments, power calculations for animal experiments are based on our previous work [11, 18, 32]. In experiments conducted with NW1 cells or 4T1 cells, 60–70% of animals have metastasis. Assuming a power of 80% and a level of significance of 5%, we estimated that we will be able to measure a difference of 60% or greater with ten animals per group, using a Mann–Whitney test. Prior to anaesthesia, mice were randomised in two groups that were then injected with control or experimental cancer cells.

For TaqMan™ low-density array (TLDA) analysis, CT values were normalised by global mean normalisation (GMN) [33]. Resulting delta CT values were then converted in their linear numbers (2^-ΔCT^), and subjected to quantile normalisation (QQ) [34] followed by differential expression unpaired analyses (DeT, Student’s *t* test, *P* < 0.05 being considered significant) using R package DEGandMore (https://github.com/zhezhangsh/DEGandMore). Multiple comparisons have been adjusted by Benjamini–Hochberg False Discovery Rate (FDR). The log2-ratio of group means and −log10 (*P* values) were used to generate volcano plots using R package VolcaNoseR (https://github.com/JoachimGoedhart/VolcaNoseR), and Z-score of 2^-ΔCT^ values were used as an input for generating heat maps using Broad institute software Morpheus (https://software.broadinstitute.org/morpheus/).

For Cancer cell line Encyclopaedia (CCLE) analysis, a database with information on the miRNA content of cell lines obtained by RNA-seq [35] has been used to retrieve information of miRNA expression of 44 BC cell lines that belonged to different BC subtypes (Supplementary Table S2). The violin plot was used to visualise the distribution of miRNA expression levels, which were reported as log2 read counts, and the statistical significance was evaluated using the unpaired *t* test, where values of *P* < 0.05 were considered significant.

For ClueGo-based analysis, gene/protein networks with a ‘Term *P* value’ <0.05 and ‘Group *P* value’ <0.05 were identified as statistically significant.

## Results

### High levels of miR-662 in the serum of early-stage BC patients is associated with future relapse in bone

**Fig. 1:**
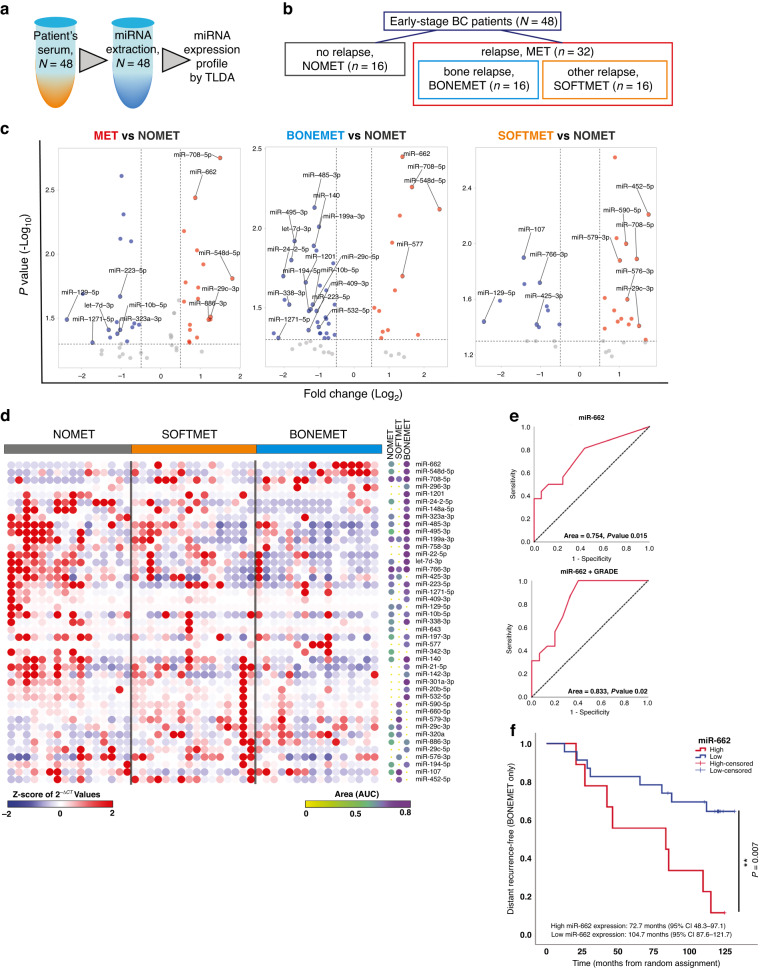
Screening of circulating miRNAs by TaqMan low-density array (TLDA) in serum of early-stage ER + BC patients at the time of surgery. **a** Schematic workflow on serum sample processing of 48 early-stage ER + BC patients. Serum samples have been used for miRNA-specific extractions, and the expressions of 646 human miRNAs and 1 unrelated to mammalian species miRNA (ath-miR159a) has been quantified by TLDA. **b** Dendrogram showing how BC patient have been divided in groups based on their clinical information over a 10-year period. The initial cohort of 48 early-stage BC patients has been first divided in a first group of 16 BC patients who did not show any metastasis recurrence (NOMET) and a second of 32 BC patients who experienced metastasis recurrence over this period (MET). In addition, MET group has been further divided in two sub-groups based if the primary site of metastasis was bone (BONEMET, *n* = 16) or other soft tissues (SOFTMET, *n* = 16). **c** Volcano plots of de-miRNAs which expression resulted significative (log_2_FC > 0.5 or log_2_FC <-0.5, *P* < 0.05) in MET versus (vs) NOMET, BONEMET vs NOMET, and SOFTMET vs NOMET comparisons. Total numbers of DE-miRNAs down- or upregulated is reported for each comparison at the top for their respective volcano plot. MiRNA name is specified for whose miRNAs which expression resulted significative also for ROC analyses (AUC > 0.70, asymptotic signature < 0.05). **d** Heatmap showing the expression levels of DE-miRNAs (-2 ≥ Z-score of 2^-ΔCT^ values ≤ 2) and their relative ROC area (0 ≥ AUC ≤ 0.8). **e** Receiver operating characteristic (ROC) curves analysis for miR-662 to predict bone relapse, either alone (upper panel) or in combination with patient’s tumor grade (GRADE) (lower panel). **f** Distant recurrence-free survival for high or low levels of circulating miR-662 levels in early-stage BC patients who develop or not skeletal metastasis (BONEMET, NOMET).

We used TaqMan™ low-density arrays (TLDA) to screen circulating miRNA expression levels in the serum of 48 early-stage ER + BC patients at baseline (time of surgery), from whom we had long-term clinical follow-up information (>10 years), including subsequent development of metastasis [16]. Specifically, we quantified the expression levels of 646 human miRNAs (Fig. 1a and Supplementary Table S1) in order to identify circulating miRNAs associated with future metastatic recurrence (MET) in bone (BONEMET) or soft tissues (SOFTMET), compared to patients who did not relapse (NOMET) (Fig. 1b and Table 1). As a result of differential expression (DE) analyses, several miRNAs were differentially expressed (DE-miRNAs) according to the metastasis status of BC patients with 36 DE-miRNAs associated with MET, and 45 and 40 DE-miRNAs associated with BONEMET or SOFTMET, respectively (Fig. 1c, d and Supplementary Table S3). ROC analysis was conducted for DE-miRNAs to test their sensitivity and specificity to predict metastatic recurrence (Fig. 1d and Supplementary Table S4). Interestingly, hsa-miR-662 (here, abbreviated miR-662), the most overexpressed DE-miRNA in the serum of patients who first relapsed in bone, showed a good sensitivity (area under the curve, AUC = 0.754; *P* = 0.015) to predict metastatic relapse (Fig. 1d, e). Serum miR-662 expression levels combined with tumour grade had a higher predictability for the risk of bone relapse (AUC = 0.833, *P* = 0.02) compared to miR-662 levels alone (Fig. 1e). Furthermore, Kaplan–Meier survival analysis revealed that the risk of bone metastasis was >threefold increased for patients with high circulating levels of miR-662 (HR = 3.36, *P* = .02), compared to those who had low circulating levels (Fig. 1f). No changes in miR-662 expression levels were observed in the serum from patients who received zoledronic acid (ZA) treatment, when all patients were considered (NOMET + MET), compared to those who did not receive ZA treatment, or when patients receiving ZA treatment were stratified according to the type of relapse (MET, NOMET, BONE MET) (Supplementary Fig. S3). Taken together, these results suggest that ZA treatment did not interfere with miR-662 ability to predict relapse.

Overall, our results show that miR-662 is associated with future (bone) relapse, and the quantification of miR-662 expression levels in the serum of early-stage BC patients might be a prognostic tool to predict the risk of disease progression toward metastasis.

### MiR-662 expression is independent of BC subtypes, and its overexpression increases metastatic abilities of human BC cells

**Fig. 2:**
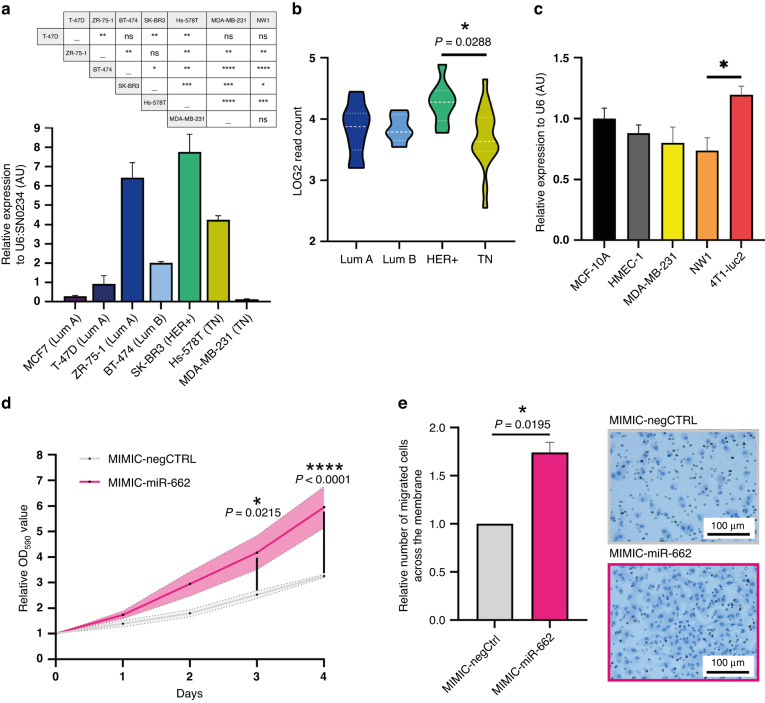
MiR-662 expression in BC subtypes and miR-662 overexpression effect on BC cell proliferation and migration properties. **a** Evaluation by real-time qPCR of miR-662 expression in human BC cell lines (T-47D, MCF7, ZR-75-1, BT-474, SK-BR3, Hs-578T, MDA-MB-231) that belong to luminal, TN or HER + BC subtypes. Relative expressions were obtained by comparing ΔCTs for miR-662 amplification to ΔCTs of U6:SNO234 housekeeping genes. **b** MiR-662 expressions (log_2_ Read Count) retrieved from CCLE database of BC cell lines which belong to luminal, TN and HER + BC subtypes. **c** Evaluation by real-time qPCR of miR-662 basal expression in human normal cell lines (MCF-10A, HMEC-1), BC cell lines (MDA-MB-231 and its engineered luc2-positive subpopulation, NW1), and an engineered murine BC cell line (4T1*-luc2*). Relative expressions were obtained by comparing ΔCTs for miR-662 amplification to ΔCTs of U6 as housekeeping gene. **d** Proliferation assay. MiR-662 overexpression promoted cell proliferation in MDA-MB-231 cells compared to control. The significance is observed starting by day 3. **e** Transwell migration assay. MiR-662 overexpression promoted cell migration across the membrane in MDA-MB-231 cells compared to control. Migrated cells have been manually counted on the entire surface of the membrane. Representative images of haematoxylin/eosin-stained control and miR-662-overexpressing cells migrated through the membrane are shown. Means of three independent experiments ± SEM were shown for all experiments, *P < 0.05, **P < 0.01, ***P < 0.001, ****P < 0.0001.

The role of miR-662 in BC is unknown. To determine its role in BC and metastasis, we first assessed miR-662 expression levels in established human BC cell lines by real-time qPCR (Fig. 2a). Irrespective of BC subtypes, miR-662 expression levels varied greatly from one cell line to another in luminal [oestrogen receptor (ER)-positive and/or progesterone receptor (PR)-positive] and triple negative (TN) [EGF receptor2 (HER2)-negative, and ER- and PR-negative] BC cells, with the highest expression levels shown by HER2-positive (HER + ) SK-BR3 cells (Fig. 2a). A similar trend was observed when using expression levels (Log2 Read Count) from 45 different human BC cell lines retrieved from the Cancer Cell Line Encyclopaedia (CCLE) (Fig. 2b and Supplementary Table S2). Overall, we found low-to-moderate miR-662 expression levels in human BC cell lines, irrespective of the luminal or TN BC subtype, with significantly higher (*P* = .0288) miR-662 expression levels in HER+ cell lines compared to TNBC cell lines only (Fig. 2b). Furthermore, miR-662 expression levels in TNBC MDA-MB-231 cells and their luciferase-expressing cell subpopulation, MDA-MB-231-*luc2*-NW1 (NW1), were similar to those observed in human MCF-10A and HMEC-1 normal epithelial cell lines (Fig. 2c). These results suggested that aggressive properties typical of TNBC cells have been acquired irrespective of endogenous miR-662 expression levels. We have also evaluated basal miR-662 expression levels in a murine TNBC cell line, 4T1-*luc2*, which is a spontaneous model for breast cancer metastasis in vivo. We found similar miR-662 expression levels compared to normal epithelial cells, and a significative increase compared to NW1 cells (Fig. 2c), although those levels remained low compared to those observed in other BC cell lines. Thus, because of these low miR-662 endogenous levels, we reasoned that TNBC MDA-MB-231, NW1 and 4T1-*luc2* cells were convenient models to conduct gain-of-function experiments in order investigate the role of miR-662 in breast cancer metastasis progression. Moreover, MDA-MB-231 cells offer useful application to study bone metastasis in vivo for its independency to oestrogen and its intrinsic bone and visceral tropism once cells are inoculated in the bloodstream [12, 36]. By contrast, hormone-sensitive BC cell models in mice are more appropriate to study earlier steps of metastatic progression, such as first steps of tumour cell escape from primary tumour xenografts [37].

MiRNAs mimics are synthetic double-stranded RNA oligonucleotides that imitate endogenous miRNA duplexes, and are therefore currently used to study biological functions of miRNAs [38]. We first explored miR-662 functions in TNBC cells by transfecting miR-662 mimics (MIMIC-miR-662) or scramble miRNA mimics (MIMIC-negCTRL) in the MDA-MB-231 cell line, which resulted in a transient overexpression of miR-662 that lasted for several days (>8 days) (Supplementary Fig. S1). We then performed proliferative and transwell migration in vitro assays, observing that miR-662 transient overexpression significantly increased MDA-MB-231 cell proliferation (*P* = 0.0215 at day 3; *P* < 0.0001 at day 4) (Fig. 2d) and migration (*P* = 0.0195) (Fig. 2e), compared to control transfected cells. These results suggested that high expression levels of miR-662 may potentially promote metastatic abilities of BC cells in vivo.

### MiR-662 overexpression in BC cells promotes the metastatic penetrance of tumour cells in the skeleton over time

**Fig. 3:**
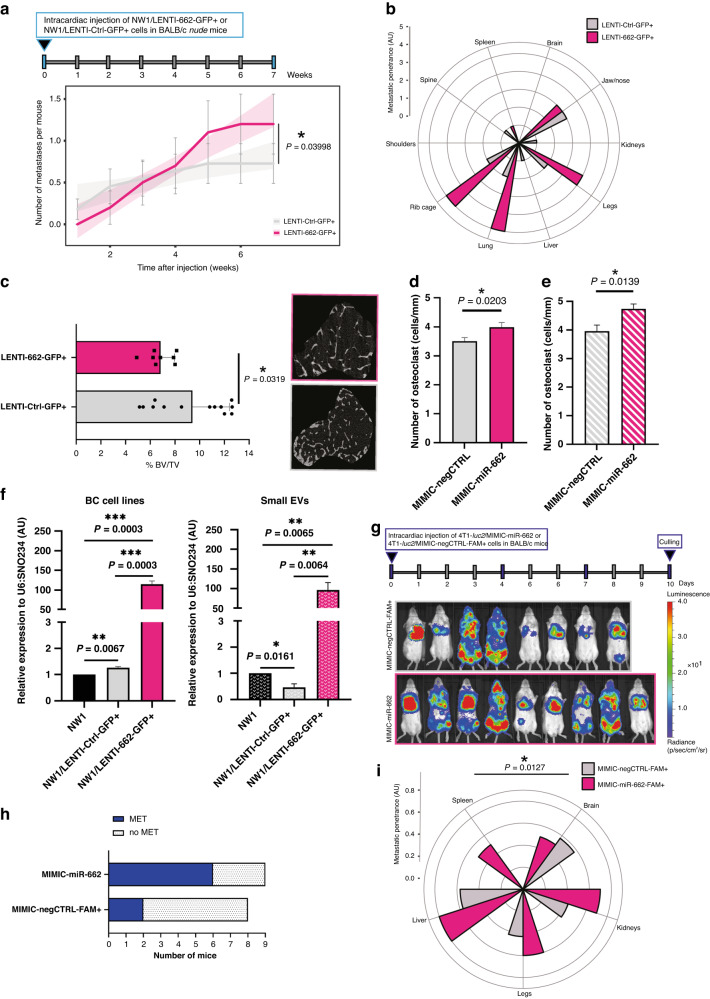
MiR-662 overexpression in BC cells promotes bone metastasis formation in vivo. **a** Upper part: Experimental design used with the immunocompromised model of BC bone metastasis, where intracardiac injections of NW1/LENTI-662-GFP+ (*N* = 10) or NW1/LENTI-Ctrl-GFP+ (*N* = 11) BC cells were injected intracardially to Balb/c *nude* mice. Lower part: Metastatic incidence (weekly recorded by BLI) of animals in the control or experimental group over a 7-week period. **b** Petal plot showing metastatic penetrance of miR-662 overexpressing cells in BC cells compared to control. Overt metastases have been evaluated by ex vivo imaging in several organs (brain, jaw/nose, kidneys, legs, liver, lung, rib cage, shoulders, spine, spleen). **c** Histograms showing *micro*-CT analysis of trabecular thickness as percentage of bone volume over total volume (%BV/TV) for left and/or right tibia of representative mice of experimental or control groups. Representative images of the trabecular area analysed for the control or experimental group. **d** Number of active osteoclasts (cells/mm) in TRAP-stained tibial sections of mice injected with NW1/LENTI-662-GFP+ cells compared to control. **e** Number of active osteoclasts (cells/mm) in TRAP-stained tibial sections of mice injected with NW1/LENTI-662-GFP+ cells compared to control that presented tumours in their legs. **f** MiR-662 expression levels in BC cell lines (parental NW1, NW1/LENTI-Ctrl-GFP + , NW1/LENTI-662-GFP + , SK-BR3) and in their respective BC cell-derived small extracellular vesicles (small EVs, sEVs) collected from conditioned media and then isolated by ultracentrifugation. Means of three independent experiments ± SEM were shown, *P < 0.05, **P < 0.01, ***P<0.001. **g** Upper part: Experimental design of the used in vivo syngeneic model of BC bone metastasis, where intracardiac injections of 4T1-*luc2*/MIMIC-miR-662 (*N* = 9) or 4T1-*luc2*/MIMIC-negCTRL-FAM+ (*N* = 8) BC cells were performed on BALB/c mice. Mice showed a first evidence of metastasis by day 4, and the experiment was concluded at day 10. Lower part: In vivo bioluminescent imaging (BLI) of *luc2*-positive tumour-bearing mice at day 7. BLI has been taken at same exposure time from luciferin injection, the bioluminescence is expressed in radiance (photons/second, ph/s), and the same scale of intensity has been used for all photographs. **h** Histogram showing the incidence of skeletal metastasis in control or experimental mice. **i** Petal plot showing metastatic penetrance of miR-662 overexpressing cells in BC cells compared to control. Overt metastases have been evaluated by ex vivo imaging in several organs (brain, kidneys, legs, liver, and spleen). Significance is shown as *P < 0.05, **P < 0.01, ***P < 0.001.

We showed that in the clinic high circulating levels of miR-662 at baseline were associated with a higher risk for BC patients to experience bone relapse years after primary tumour resection (Fig. 1). We also observed that miR-662 overexpression promoted metastatic abilities of BC cells in vitro (Fig. 2). Taken together, these findings prompted us to investigate the role of miR-662 in bone metastasis. Therefore, MDA-MB-231-*luc2*-NW1 (NW1) cells were stably transduced and selected using the lentiviral vector hCMV-TurboGFP encoding miR-662 (NW1/LENTI-662-GFP + ) or the control vector (NW1/LENTI-Ctrl-GFP + ). MiR-662 expression levels in the NW1/LENTI-662-GFP+ and control cell lines were validated by real-time qPCR (Supplementary Fig. S4). The stable overexpression of miR-662 increased cell proliferative and migratory properties of NW1 cells compared to control (Supplementary Fig. S5), as previously observed for MIMIC-662 transient transfection (Fig. 2). Transduced cells were then intracardially injected in BALB/c *nude* mice to mimic dissemination and metastatic outgrowth of BC cells in bone [36], and skeletal tumour burden was monitored weekly by BLI up to 7 weeks after tumour cell inoculation (Fig. 3a and Supplementary Fig. S6). Compared to the control group, we observed that the skeletal tumor burden in mice inoculated with miR-662-overexpressing tumor cells was attenuated during the first weeks, which corresponded to the early phase of bone metastasis formation (Fig. 3a). However, at a later stage of the bone metastatic process, miR-662 then enhanced metastatic tumor burden progression (*P* = 0.03998), leading to a higher extent of osteolytic lesions in these animals when compared to the control group (Fig. 3a). At the time of the culling of animals, we then assessed the metastatic penetrance ex vivo of NW1/LENTI-662-GFP+ and NW1/LENTI-Ctrl-GFP+ tumour cells in the skeleton and soft organs (lung, liver, brain, spleen, kidney) using BLI, and we found that miR-662-overexpressing tumour cells were more prone to disseminate in the skeleton and lungs compared to control tumour cells (Fig. 3b). Histomorphometric analysis of the trabecular region of tibiae from tumour-bearing animals showed that the bone volume/tissue volume (BV/TV) ratio was substantially decreased (*P* = 0.0319) in the miR-662-overexpressing group compared to control (Fig. 3c and Supplementary Fig. S6), which was indicative of an increased bone destruction in animals bearing miR-662-overexpressing tumours. We also observed an increase (*P* = 0.0203) in the number of active osteoclasts (cells/mm) in tibial sections of mice injected with NW1/LENTI-662-GFP+ cells compared to control (Fig. 3d and Supplementary Fig. S6). When we compared tumour-bearing tibiae between miR-662-overexpressing and control groups, there was still a significant difference (*P* = 0.0138) in the number of osteoclasts in tibial sections of mice injected with NW1/LENTI-662-GFP+ cells (Fig. 3e), whereas this difference was lost when only comparing tumour-free tibiae from both groups (Supplementary Fig. S7). Taken together, these results indicated that the increase in the number of osteoclasts was specifically due to the presence of miR-662-overexpressing tumour cells in bone. Additionally, we evaluated the ability of miR-662-overexpressing tumour cells to secrete miR-662 as cargo of small extracellular vesicles (sEV). Thus, sEV were isolated by ultracentrifugation of the sEV fraction from conditioned media of NW1/LENTI-662-GFP+ and NW1/LENTI-Ctrl-GFP+ cells, and parental NW1 cells (low miR-662 basal levels). We found that miR-662-overexpressing cells secreted higher levels of miR-662 in sEVs compared to control and parental NW1 cells (*P* = 0.0064 and *P* = 0.0065, respectively), mirroring miR-662 expression of these BC cell lines (Supplementary Fig. 3f).

To evaluate the metastatic capacity of miR-662-overexpressing tumour cells in the presence of a fully functional immune system, we utilised a syngeneic animal model in which highly invasive and metastatic murine 4T1-*luc2* BC cells, transiently transfected with MIMIC-miR-662 or negCTRL-FAM + , were injected intracardially into BALB/c mice 72 h-post transfection (Fig. 3g). While all animals showed metastasis in the lungs, which is usually the first site of metastasis of these cells, 9 days after tumour cell injection as judged by BLI detection (Fig. 3g), animals bearing miR-662-overexpressing tumour cells tended to have more frequent skeletal metastases in the hindlimbs compared to that observed in the control group (66.67% vs 25%, respectively; *P* = 0.103) (Fig. 3h). Using BLI imaging, we then examined ex vivo the metastatic penetrance of tumour cells in the skeleton and soft organs other than lungs (liver, brain, spleen, kidney), and we observed that miR-662-overexpressing tumour cells were in general more prone to disseminate to all organs, except for brain, compared to control tumour cells (Fig. 3i). Overall, there was an increase (*P* = 0.0127) in the metastatic penetrance of BC cells overexpressing miR-662 compared to control cells (Fig. 3i). Taken together, these results obtained in two different animal models of BC metastasis suggested that miR-662 in BC cells promoted the metastatic penetrance of tumour cells in the skeleton over time.

### MiR-662 attenuates skeletal tumour burden at an early stage of bone metastasis formation in vivo due to an inhibitory effect on osteoclast differentiation

Although metastasis is a late event during cancer progression, it has been shown that the systemic spread of BC cells is an early event, with disseminated tumour cells being present in distant organs even when the primary tumour is not yet clinically detectable [1]. It has been proposed there exists a latent phase preceding the metastatic phase during which disseminated cancer cells first have to survive and adapt in the bone microenvironment in order to grow and subsequently form macroscopic bone metastases [1]. Interestingly, our results obtained in mice model inoculated with NW1 cells overexpressing miR-662 (Fig. 3) showed that skeletal tumour burden was attenuated during the first weeks after miR-662 overexpressing cell injection compared to the control group, suggesting a bi-phasic effect of miR-662 during bone metastasis formation in vivo.

**Fig. 4:**
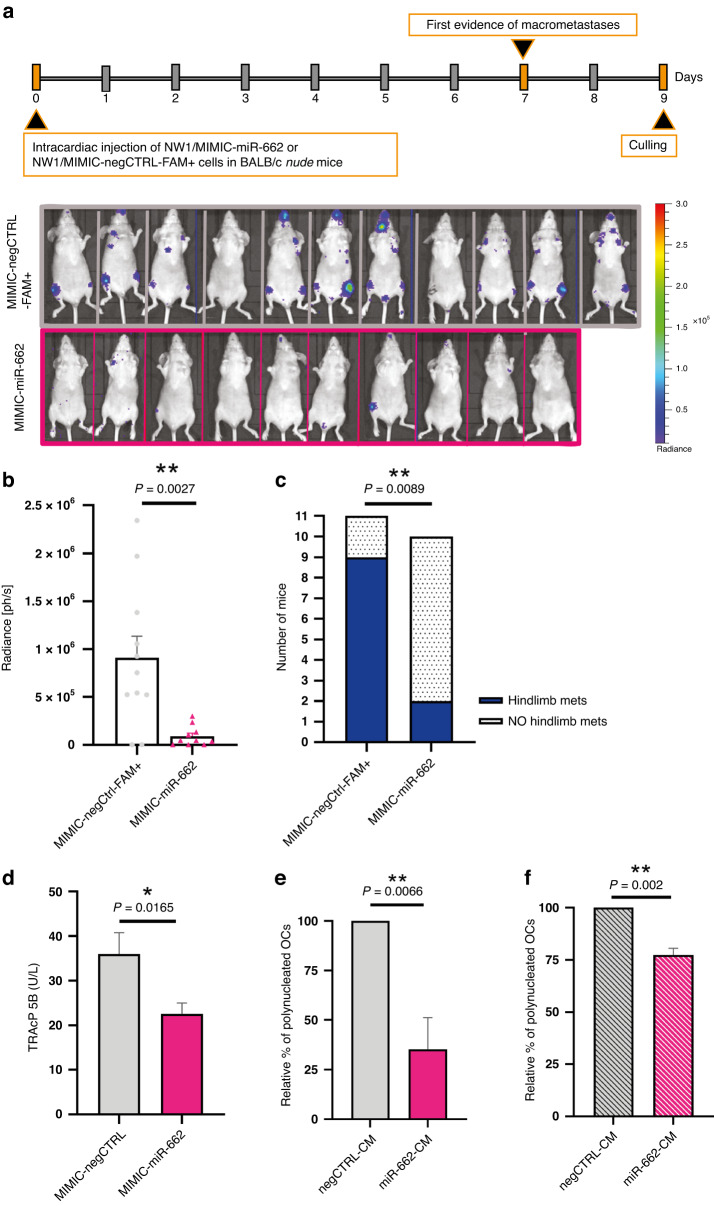
MiR-662 overexpression inhibits early-stages of bone metastasis formation in vivo and reduces osteoclast maturation. **a** Upper part: Experimental design used with the immunocompromised model of BC bone metastasis, where NW1/MIMIC-miR-662 (*N* = 10) or NW1/MIMIC-negCTRL-FAM+ cells (*N* = 11) BC cells were injected intracardially to Balb/c *nude* mice. Lower part: In vivo bioluminescent imaging (BLI) of mice at day 7. Images have been taken at same time exposure from luciferin injection, the bioluminescence is expressed in radiance (photons/second, ph/s), and the same scale of intensity has been used for all photographs. **b** Histogram showing the total tumour intensity (ph/s) of overt metastases in the whole-body of each mouse of control or experimental group. **c** Histogram showing the incidence of hindlimb metastasis in control or experimental mice. **d** Serum concentrations of TRAcP 5B (U/L) measured by ELISA. **e** Histogram showing relative percentages of mature, polynucleated murine osteoclasts (OCs) in presence of conditioned medium collected from NW1/MIMIC-miR-662 or control cells. **f** Histograms showing relative percentages of mature, polynucleated human donor-derived osteoclasts (OCs) in presence of conditioned media collected from NW1/MIMIC-miR-662 or MCF7/ MIMIC-miR-662 and their respective controls. Means of three independent experiments ± SEM are shown, *P < 0.05, **P < 0.01.

This observation encouraged us to investigate the possible effect of miR-662 in BC latency in bone. Thus, NW1 cells were transiently transfected with MIMIC-miR-662 or MIMIC-negCTRL-FAM+ and injected into immunocompromised female BALB/c *nude* mice 72h-post cell transfection. To specifically evaluate first steps of BC metastasis progression, mice were sacrificed 9 days after tumour cell inoculation (short-term experiment), at which time animals in the control group had evidence of skeletal tumour burden, as shown by bioluminescence imaging (BLI) (Fig. 4a). We found a >tenfold decrease in overall tumour burden and a substantial reduction in the number of BLI-positive hindlimbs of mice injected with NW1/MIMIC-miR-662 cells in comparison with control cells (Fig. 4b, c, respectively). Overall, these results obtained with an acute miR-662 overexpression (achieved by miR-662 MIMIC transient overexpression) in NW1 cells were similar to those obtained with stably miR-662-overexpressing NW1 cells when monitoring, in the same animal model, the skeletal tumour burden 7 to 9 days after tumour cell inoculation (Fig. 3).

Since BC cells usually promote osteolytic lesions by stimulating osteoclast resorption and repressing osteoblast formation, which ultimately results in an imbalance of bone homeostasis [1], we questioned whether miR-662 had any influence on bone resorption and/or bone formation at an early stage of bone metastasis formation in vivo. Compared to control, we found a statistically significant decrease of tartrate-resistant acid phosphatase-5b (TRAcP 5b) serum levels, a marker of osteoclast activity, in animals inoculated with NW1/MIMIC-miR-662 cells (Fig. 4d), whereas serum levels of pro-collagen type I N propeptide (P1NP), a bone formation marker, remained unchanged (Supplementary Fig. S8). We then investigated whether miR-662 had any influence on osteoclast differentiation in vitro. To address this question, we performed osteoclastogenesis assays where cultured primary murine, bone marrow-derived monocytes were treated with pro-differentiation factors (RANK-L and M-CSF) in the presence of the conditioned medium (6.25% v/v) from transfected NW1/MIMIC-miR-662 or NW1/MIMIC-negCTRL cells. Consistent with in vivo data (Fig. 4d), the number of TRAP-positive multinucleated osteoclasts was decreased by ~65% in the presence of the conditioned medium from NW1/MIMIC-miR-662 cells, when compared with that observed for the conditioned medium of NW1/MIMIC-negCTRL cells (Fig. 4e and Supplementary Fig. S9). Furthermore, osteoclast differentiation experiments were conducted using peripheral blood mononuclear cells from healthy human donors, and an inhibitory effect on human osteoclast differentiation was observed with the conditioned medium from human NW1/MIMIC-miR-662 cells compared to control cells (Fig. 4f and Supplementary Fig. S10).

Overall, these data suggested that miR-662 inhibits osteoclast-mediated bone resorption preventing tumour growth at an early stage of metastasis progression in bone. However, even if miR-662-overexpressing BC cells can remain indolent for a period, we have clinical (Fig. 1) and experimental (Fig. 3) evidence that BC cells overtake this indolent phase to then proliferate into (clinically) detectable overt metastasis. Thus, we further investigated the molecular mechanism that could stimulate the expansion of miR-662-overexpressing BC cells in bone, leading to an increase of tumour burden in murine models and bone relapse in early-stage BC patients.

### MiR-662 promotes metastasis by inducing a stem-like phenotype to BC cells

**Fig. 5:**
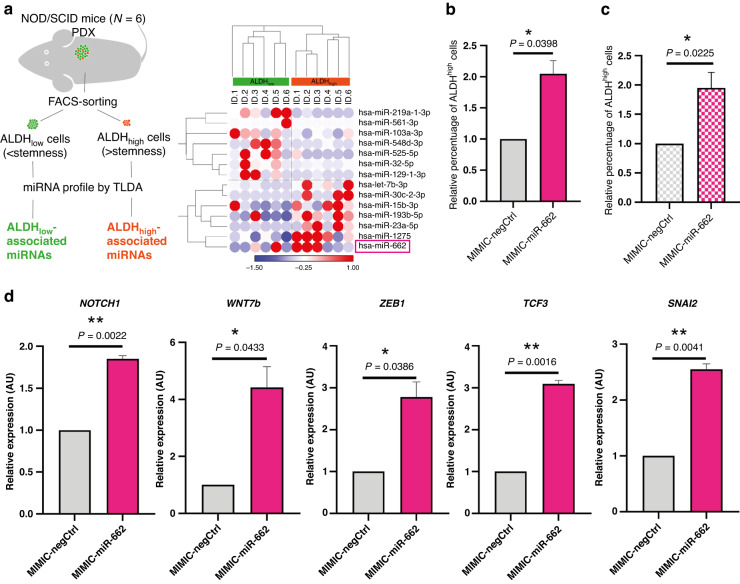
MiR-662 is associated with the ALDH^high^ BC cell subpopulation, and miR-662 overexpression promotes the expression of stem-related genes in vitro. **a** Left-hand panel: Experimental design used with the patient-derived xenograft (PDX) model where ER+ or TN primary breast tumours were implanted in the fat pad of NOD/SCID immunodeficient mice (*N* = 6). Single tumour cells retrieved from xenografts were FACS-sorted according to their aldehyde dehydrogenase (ALDH) enzymatic activity levels (ALDH^high^ or ALDH^low^). Then, miRNA profile by TLDA was conducted on ALDH^high^ and ALDH^low^ cells. Right-hand panel: Heatmap of DE-miRNAs between ALDH^high^ and ALDH^low^ BC cells. MiR-662 is overexpressed in ALDH^high^ cells. **b** Histogram showing the relative percentage of ALDH^high^ BC cells upon overexpression of miR-662 mimic (MIMIC-miR-662) or the mimic negative control (MIMIC-negCTRL) in MDA-MB-231 cells grown in a monolayer evaluated by flow-cytometry. **c** Same experiment of panel B, but with MDA-MB-231 cells grown in as 3D organoid (tumorsphere) after 7 days from transfection. **d** Evaluation by RT-qPCR of stemness-related human genes (*NOTCH1*, *WNT7b*, *ZEB1*, *TCF3*, *SNAI2*) in miR-662-overexpressing cells in comparison with control cells. Relative expressions were obtained by comparing ΔCTs for each gene amplification to the mean ΔCT value between U6 and SNO234 HKGs. Means of three independent experiments ± SEM were shown, *P < 0.05, **P < 0.01.

Recently, BC stemness has been pointed out as one of the key features that highly tumorigenic BC cells have, allowing them to better succeed in colonising new microenvironments and being often associated with drug resistance, self-renewal, and immune escape mechanisms [39, 40]. In order to determine whether miR-662 expression could be specifically associated with the stem-like subpopulation in BC primary tumours, we performed patient-derived xenograft (PDX) experiments using ER+ or TN primary breast tumours (*N* = 6) that were implanted in the fat pad of NOD/SCID immunodeficient mice (Fig. 5a). At the time of tumour resection, each tumour xenograft was dissociated into a single-cell suspension, and cells were FACS-sorted according to their aldehyde dehydrogenase (ALDH) enzymatic activity levels, a well-established marker of stemness, where high levels (ALDH^high^) are an indication of a stem-like phenotype [17]. MiRNA expression levels in ALDH^high^ and ALDH^low^ tumour subpopulations were then screened by TLDA. As shown in Fig. 5a, miR-662 was among miRNAs that were highly expressed in ALDH^high^ primary BC cells. To validate these results in vitro, we transiently overexpressed miR-662 (MIMIC-miR-662) or a miRNA negative control (MIMIC-negCTRL) in MDA-MB-231 cells, and we evaluated by flow-cytometry the effect of miR-662 overexpression on the percentage of ALDH^high^ cells 72 h post-transfection. Compared to a negative control, miR-662 overexpression induced a >twofold increase (*P* = 0.0398) of the ALDH^high^ cell subpopulation of MDA-MB-231 cells, when grown in monolayer (Fig. 5b and Supplementary Fig. S11). Similarly, transient miR-662 overexpression of MDA-MB-231 cells grown under non-adherent conditions as tumour spheres (3D organoids) led to a >1.5-fold increase (*P* = 0.0225) of the ALDH^high^ cell subpopulation compared to a negative control (Fig. 5c and Supplementary Fig. S12).

We then explored by real-time qPCR the impact of miR-662 overexpression in MDA-MB-231 cells on expression levels of well-established stemness markers that are involved in embryogenesis and cancer –*NOTCH1*, *WNT7b*, *ZEB1*, *TCF3*, *CTNNB1*, *CD44*, *CD24*, *SNAI2, OCT-04*, *c-MYC*, *EZH2*– [41, 42]. Since these genes were predicted not to be direct targets of miR-662 using TargetScan prediction tool (Supplementary Table S6), cells were collected after 6 days post-transfection. Compared to a negative control mimic, *NOTCH1*, *WNT7b*, *ZEB1*, *TCF3* and *SNAI2* mRNA expression levels were significantly increased in miR-662-overexpressing MDA-MB-231 cells (Fig. 5d), whereas no difference was observed for *OCT-04*, *c-MYC*, *CD44*, *CD24*, *CTNNB1* and *EZH2* mRNA expression levels (Supplementary Fig. S13). In addition, we performed a ClueGo-based analysis [28] on the top 200 predicted targets of miR-662 to evaluate which gene/protein functionally organised in GO/pathway networks could potentially be repressed by miR-662 biological functions. We identified 106 gene/protein networks (Term *P* value <0.05, Group *P* value < 0.05), among Gene Ontology (GO) terms, Kyoto Encyclopedia of Genes and Genomes (KEGG) pathways and Human Reactome (R-HSA) database (Supplementary Table S6 and Supplementary Figs. S14 and 15). Interestingly, in the gene network which refers to *‘DOT1L (KMT4) methylates methyl-lysine-80 of histone H3 (H3K79)*’ we found reactome pathways involving SIRT6, NOTCH1, and NOTCH4 genes, which are very well known to regulate cell stemness [43]. We also observed that predicted targets for miR-662 were involved in a gene network identified as ‘*Chromatin Silencing at Telomere*’, suggesting that miR-662 could potentially affect gene transcription efficiency, thus global protein production, an additional feature associated with cell stemness [44–47].

**Fig. 6:**
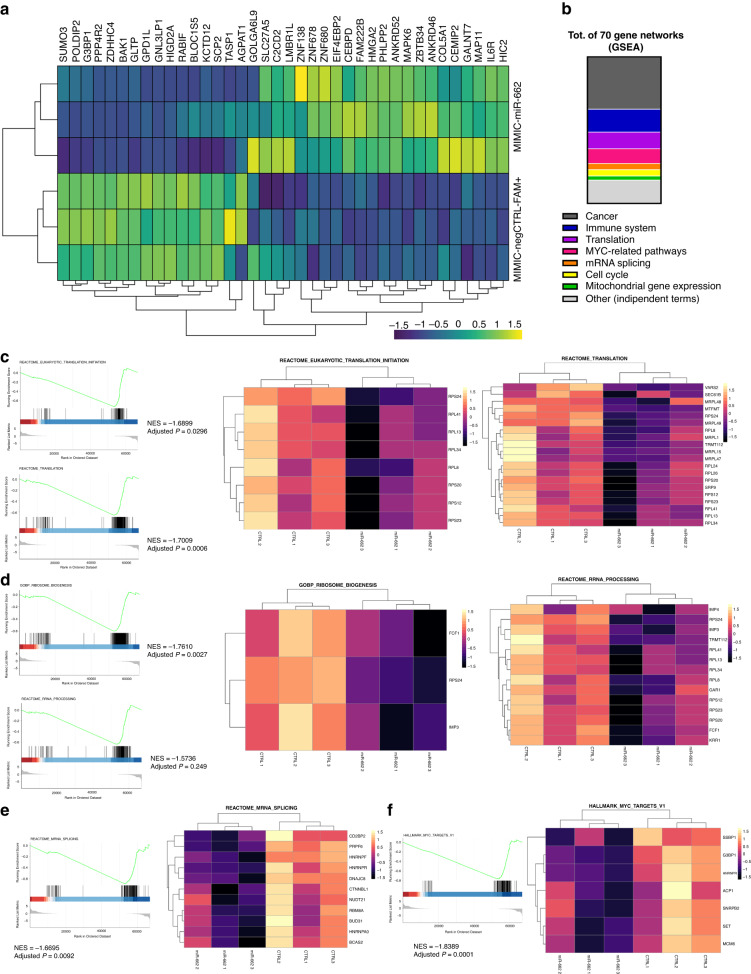
MiR-662 overexpression reduces global protein synthesis, enhancing stem-like traits of BC cells. **a** Heatmap showing expression levels of the 38 significantly deregulated DE-mRNAs (21 upregulated, 17 downregulated) in miR-662-overexpressing NW1 cells compared to mock-transfected cells obtained by RNA-seq. **b** Manual annotation of the 70 gene networks being affected by miR-662 overexpression in NW1 cells compared to mock-transfected cells obtained by GSEA analysis on RNA-seq data. Gene networks (represented as part of the whole) have been classified in 7 different groups, or reported as independent terms (8th group). **c** GSEA on RNA-seq data of gene networks involved in translation. Hallmark genes in translation were repressed by miR-662 overexpression compared to control cells. **d** GSEA of gene networks involved in ribosome biogenesis and rRNA processing. Hallmark genes were repressed by miR-662 overexpression compared to control cells. **e** GSEA of a gene network involved in mRNA splicing. Hallmark genes were repressed by miR-662 overexpression compared to control cells. **f** GSEA of a gene network that involves the MYC pathway and its targets. Hallmark genes were repressed by miR-662 overexpression compared to control cells.

We performed RNA sequencing (RNA-seq) of NW1 cells transfected with MIMIC-miR-662 or MIMIC-negCTRL-FAM+ at 36 h post-transfection to identify early transcriptomic alterations induced by miR-662 overexpression, a strategy used also in a previous published work [21]. Expression levels of 38 DE-mRNA transcripts were significantly deregulated (21 upregulated, 17 downregulated) in miR-662-overexpressing tumour cells compared to mock-transfected cells (Fig. 6a and Supplementary Fig. S16). Among DE-mRNAs, we observed genes previously reported in facilitating BC cell migration and proliferation (*CEMIP*) [48], BC bone metastasis formation (*IL6R*) [49, 50] and/or bone remodelling (*SUMO3*, *POLDIP2*) [51, 52] (Fig. 6a and Supplementary Fig. S16), reinforcing our experimental results. Additionally, the up-regulation of *HMGA2* and *IL6R* expression, which also directly contributes to BC stemness properties [53–55], and the higher expression levels of the transcription factor *C/EBPδ*, which acts upstream of IL-6 signalling through activation of *IL6R* [56], are further indication that both bone tropism and stemness traits were increased in miR-662-expressing BC cells in comparison with control cells (Fig. 6a and Supplementary Fig. S16). To further understand the overall impact of miR-662 overexpression in BC cells, we next performed gene set enrichment analysis (GSEA) on RNA-seq data. We obtained a significative enrichment in 70 gene networks mainly related to cancer, immune system, translation, and MYC-related pathways, according to a manual annotation (Fig. 6b and Supplementary Table S5). Moreover, all gene networks except one resulted with a negative enrichment score, indicating that miR-662 overexpression attenuated the activation of these pathways (Supplementary Table S5). We found particularly interesting that many of the repressed gene pathways were related to impaired protein synthesis, as shown by the inhibition of translation (Fig. 6c), ribosome biogenesis and rRNA processing (Fig. 6d), and mRNA splicing (Fig. 6e), and by the repression of the transcription factor MYC and its pathways (Fig. 6f), the latter regulating the transcription of thousands of genes and non-coding RNAs [57]. These results were in line with previous observation that inhibition of protein synthesis is associated with cancer cell stemness [44–47].

Thus, these results show that miR-662 overexpression in BC cells promotes cancer stemness, which can explain at least in part miR-662 role in promoting (bone) metastasis in animal models.

## Discussion

**Fig. 7:**
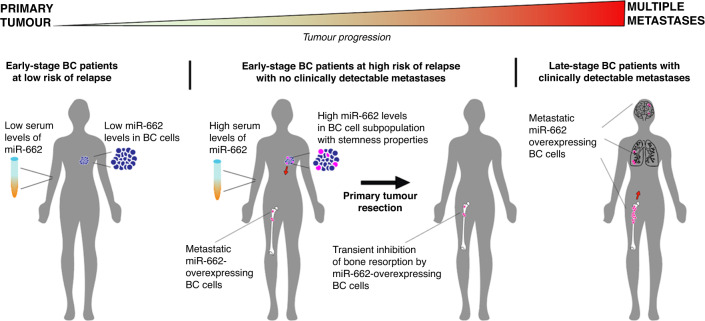
Graphical abstract. In early-stage BC patients, circulating miR-662 can be detected in serum. High levels of circulating miR-662 at the time of primary tumour resection associates with future (bone) relapse. Moreover, miR-662 overexpression is observed in the stem-like BC cell subpopulation, known to be highly tumorigenic and prone to migrate, to successfully adapt to new microenvironments, and to easily evade current treatments. Once miR-662 overexpressing cells arrives in bone, they may attenuate the development of bone metastasis through a reduction of osteoclastic resorption at an early stage of the metastatic disease. However, given the enhanced pro-metastatic properties of miR-662-expressing BC cells, this inhibition is only transient, and tumour growth resume over time, leading to the formation of clinically detectable metastases to bone and other distant organs.

Metastatic breast cancer is the most advanced stage of cancer progression, and this process can be driven by microRNAs, which post-transcriptionally regulate gene expression in cells [4]. Moreover, miRNAs can be detected in biological fluids, and miRNA expression levels can be used as biomarkers to predict the risk of disease development and/or follow its progression during time [4]. Here, we unravelled the role of miR-662 in the progression of BC towards metastasis. A functional role for miR-662 has been first described by Filipska and colleagues [10] in SCC cell lines (H520, H1703), where miR-662 inhibition decreased cell clonogenicity and motility, and sensibility to etoposide but not to cisplatin. In this work [10], miR-662 is shown to modulate transcripts involved in chemoresistance, invasiveness, EMT, and immune evasion. Here, we first showed that high miR-662 serum levels in early-stage BC patients were associated with future recurrence in bone. In the light of our experimental findings, it is highly likely that tumour-derived miR-662 was released into the bloodstream as cargo of small extracellular vesicles, which contributed to its detection in the serum of BC patients. We also demonstrated that miR-662 overexpression in human BC cells promotes metastasis in animal models. Mechanistically, miR-662 enhances tumour cell migration and proliferation as well as BC stem-like cell traits. In addition, we demonstrated that miR-662-overexpressing BC cells inhibit osteoclastic resorption, which may restrain miR-662-overexpressing BC cells from expanding in the bone marrow, but only at an early-stage of bone metastasis formation, as the acquisition of stem-like properties ultimately prevails, favouring tumour cell engraftment and leading to the formation of distant metastases, including osteolytic bone metastases (Fig. 7).

In BC, various studies demonstrated that cancer cells with stem-like properties are highly tumorigenic and more drug-, chemo- and radio-resistant [58–60], posing a challenge for the success of treatments for BC patients in the clinic. Additionally, cell stemness and specific translational signature of stem-like cells have been correlated with aggressive cell behaviour and are predictive of poor overall survival in patients, suggesting that cancer stem-like cells may be critical therapeutic targets [61, 62]. In this study, we reported that high circulating levels of miR-662 were associated with future relapse in early-stage BC patients (Fig. 1), and that miR-662 was selectively expressed in highly tumorigenic ALDH^high^ primary BC cells, irrespective of the BC subtype (Fig. 5a). So far, several stemness markers, such as CD44, CD24, and CD133, have been used to identify cancer stem cells in pre-clinical models [17, 63–65]. However, high ALDH enzymatic activity levels are also particularly suitable to identify stem cell subpopulations in BC [17, 64, 66]. Furthermore, miR-662 overexpression in MDA-MB-231 cells substantially increased mRNA expression levels of stemness markers involved in embryogenesis and cancer, such as *NOTCH1*, *WNT7b*, *ZEB1*, *TCF3*, and *SNAI2* (Fig. 5c), reinforcing the notion that miR-662 expression enhanced stem-like properties of BC cells. Additionally, the RNA-seq data analysis on transcriptomic changes due to miR-662 overexpression in BC cells revealed that *HMGA2* and *IL6R*, which both directly contribute to breast cancer stemness properties [53–55], and the transcription factor *C/EBPδ*, which acts upstream of IL-6 signalling through activation of *IL6R* [56], were upregulated in miR-662-overexpressing MDA-MB-231 cells compared to controls (Fig. 6a). Furthermore, both *IL6* and *IL6R* are involved in bone metastasis formation [1]. Thus, our results strongly suggest that miR-662 promotes BC stem cell traits, which can further correlate with a high incidence of metastasis in patients.

Recent evidence indicates that stem cells are characterised by low rates of global protein synthesis [44–47]. For example, components of the eukaryotic initiation factor 4F (eIF4F) complex, namely eIF4E and eIF4G, mediate translation programmes that are generally repressed in stem cells [45]. Specifically, eIF4E-binding proteins (eIF4E-BPs) bind to eIF4E in their unphosphorylated state and prevent its interaction with eIF4G, thereby impairing initiation of the 5’ cap-dependent translation [45]. Here, we observed that eIF4E-BP2 was highly expressed in miR-662-expressing BC cells (Fig. 6a). Moreover, we showed that multiple gene networks involved in inhibition of translation, ribosome biogenesis, rRNA processing, and mRNA splicing were repressed in miR-662-expressing BC cells (Fig. 6). Similarly, we observed the repression of the transcription factor MYC and its pathways (Fig. 6), the latter being a multipotent transcription factor and a regulator of ribosome biogenesis that promotes the eIF4F-dependent translation [45, 67]. Thus, our results strongly suggest that miR-662 impairs translational efficiency in BC leading to the low rates of protein synthesis, thus contributing to the acquisition of stem cell traits as reported by others [44–47].

It has been demonstrated that stemness and the cell reprogramming occurring during the epithelial-to-mesenchymal transition (EMT) are tightly interconnected [68, 69]. In fact, during EMT, epithelial-like cancer cells that acquire mesenchymal properties also increase their stemness traits [68], with stem-like BC cells expressing EMT markers and increased tumour-initiating capacity [69]. In our study, we demonstrated that the overexpression of miR-662 in BC cells not only increased cell stemness, but also promoted other malignant cell traits (proliferation, migration) of BC cells (Fig. 2). Moreover, among genes that were the most upregulated by miR-662 in MDA-MB-231 cells (Fig. 6a), we identified *CEMIP* (cell migration-inducing hyaluronidase 1), which has previously been shown to facilitate BC cell migration and proliferation through different signalling pathways including EMT, Wnt/ β-catenin, MEK/ERK and PI3K/Akt [48], and *GALTN7*, which promotes proliferation and invasion of different cancer cell types, including glioma, papillary thyroid carcinoma and cervical carcinoma cells [70–72]. The role of these genes in promoting breast cancer bone metastasis formation has never been reported so far. In general, while stemness is a key feature for cancer cells to adapt to new and challenging microenvironments, when combined to other aggressive properties –such as cell mobility, self-renewal and high-proliferation rate– it can result as a considerable advantage for cancer cells prone to metastasise [73]. Moreover, the cell plasticity between epithelial-, mesenchymal-, stem-like phenotypes is extremely high [74], and tumour cells quickly react to environmental stimuli being able to switch between states. This suggests that miR-662 contributes to the acquisition of a malignant cell phenotype by increasing cancer stemness and modulating pathways associated with EMT, leading to miR-662-overexpressing cells able to successfully form metastases in animal models.

Although we demonstrated that miR-662 enhanced metastatic burden in animal models (Fig. 3), we also observed that miR-662 attenuated bone metastasis development at an early phase of the metastatic progression (Fig. 4). The use of the proposed experimental model of breast cancer metastasis to the skeleton in mice is the optimal approach to evaluate the fine-tuned equilibrium established by cancer cells in bone, and its multifactorial variables, at different stages [1, 75]. Particularly, in our two in vivo studies, we deciphered both early (Fig. 4) and late (Fig. 3) stages of BC bone metastasis progression, and the bi-phasic function of miR-662 in this process. The evidence of attenuated bone metastasis at an early stage was further supported by the in vitro demonstration that conditioned media from BC cells overexpressing miR-662 mimics inhibited osteoclast differentiation of murine and human peripheral blood mononuclear cells treated with M-CSF and RANKL, compared to negative control mimics (Fig. 4). Inhibitory molecular mechanisms through which miR-662 inhibited the differentiation of monocytes into osteoclasts are unknown. However, it has previously been reported that BC cells secrete soluble factors that directly regulate osteoclast differentiation in a positive- (lysyl oxidase, interleukin 1β) or negative-manner (osteoprotegerin, endothelin-1) [1]. The hypothesis that miR-662 induced the production of some inhibitory factors, including osteoprotegerin and/or endothelin-1, warrants further investigation. Furthermore, our findings suggest that through the inhibition of osteoclastogenesis, miR-662 could influence the so-called ‘vicious cycle’, thus delaying BC cell proliferation in bone. In fact, in BC bone metastasis, the activation of osteoclastic bone resorption is usually a key step that allows BC cell sustainment and proliferation in this new and challenging environment [1]. Thus, miR-662-overexpressing cells able to seed in bone niches could have a transient latent phase before being able to proliferate as over-metastasis. Indeed, our data demonstrated that the proliferation of miR-662 in bone resumes over time, allowing tumour growth and leading to the subsequent formation of metastatic osteolytic lesions in animals, which is likely due to the creation of the feed-forward cycle usually seen with the progression of the metastatic disease. This is particularly interesting considering that in the clinic there is evidence of long period of latency between the diagnosis of primary BC and overt metastasis detection [76, 77]. This latency can be specially long in ER-positive BC tumour [78], and the mechanism for which disseminated tumour cells slowly evolve in macro-metastasis is still largely unknown, posing a challenge in the correct approach of clinicians on preventive treatments of BC patients at high risk of relapse. In this study, we provide evidence that although the overexpression of miR-662 can be the cause of a transient latency of BC cells in bone, the promotion of stem-like traits by miR-662 can be then the driving mechanism that promote BC cells to colonise, survive and expand in bone over time. One possible explanation of this switch between the arrest (latency) and reactivation (towards overt metastasis) of disseminated miR-662-overexpressing BC cells may be the presence of external stimuli, which will require further investigation.

## Conclusion

Overall, our results suggest that miR-662 acts as a BC metastasis promoter. High circulating levels of miR-662 are detected in the serum of BC patients at an early-stage of the disease, and are associated with future (bone) relapse. Thus, measuring miR-662 serum levels in BC patients at the onset of the disease might be an efficient way to monitor disease progression and/or predict the future risk of (bone) relapse, so patients could potentially benefit from preventive treatments. Moreover, miR-662 overexpression is associated with cancer cell stemness. Cancer stem cells are widely considered as a key cancer cell subpopulation able to easily evade current treatments posing a risk of a future metastatic recurrence. Additionally, they are the core of cells able to be highly tumorigenic, prone to migrate and successfully adapt to new microenvironments. Thus, our findings can provide a solid starting point for the development of new and innovative treatments targeting miRNA expression in cells to reduce the risk of metastasis development and/or therapy resistance. However, in case of miR-662, specific in vitro and in vivo experimental designs that will include the use of a carrier with miR-662 inhibitors, and further evaluation of efficiency and cytotoxicity in animal models, are needed to further address this point.

## Supplementary information

Supplementary Figures S1-S15

Supplementary Table 1

Supplementary Table 2

Supplementary Table 3

Supplementary Table 4

Supplementary Table 5

Supplementary Table 6

## Data Availability

All data presented in this work are present in the paper and/or in the Supplementary Materials. Other data that support the findings of this study are available from corresponding authors (MP and PC) on reasonable request. The dataset used or analysed during the current study are available from corresponding authors (MP and PC) on reasonable request.
