# Supplementary material for: MiR-662 is associated with metastatic relapse in early-stage breast cancer and promotes metastasis by stimulating cancer cell stemness
Source: Br J Cancer. 2023 Jul 13;129(5):754–71. doi: 10.1038/s41416-023-02340-9 (PMC10449914; doi:10.1038/s41416-023-02340-9)
Supplement: Supplementary file 1 — Supplementary Figures S1-S15 [file 41416_2023_2340_MOESM1_ESM.docx]

**Figure S1**

We first optimised MIMIC-miR-662 and MIMIC-negCTRL overexpression in MDA-MB-231-*luc2* cells using Lipofectamine2000 (ThermoFisher) as transfecting agent. For cell transfection, we tested two concentrations (5, 50 nM) of MIMIC-negCTRL-FAM+, and we evaluated the efficiency of transfection by imaging (EVOS microscope, ThermoFisher) (**PANEL A**), flow-cytometry (**PANEL B**), and real-time qPCR (**PANEL C**), using wild-type MDA-MB-231-*luc2* cells as a negative control of transfection. Then, we confirmed MIMIC-miR-662 and MIMIC-negCTRL overexpression in MDA-MB-231-*luc2* cells over time by evaluating their expression at different time points (24h, 72h, 6d, 8d) (**PANEL D**). Scale bars: 400 μm.


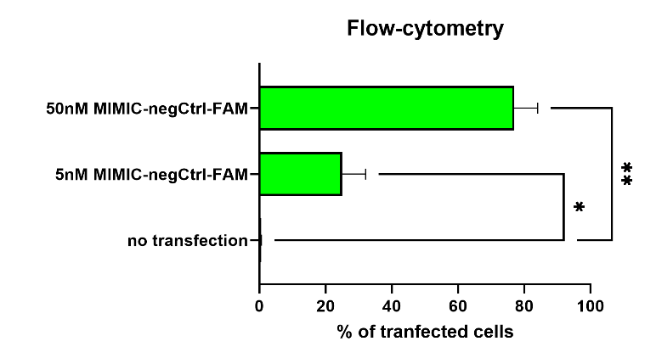

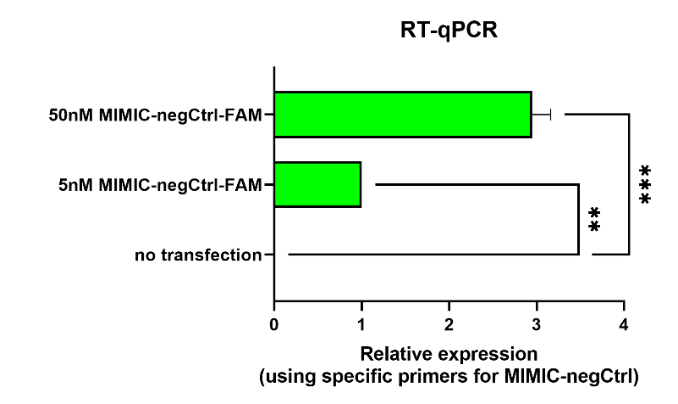


B

C


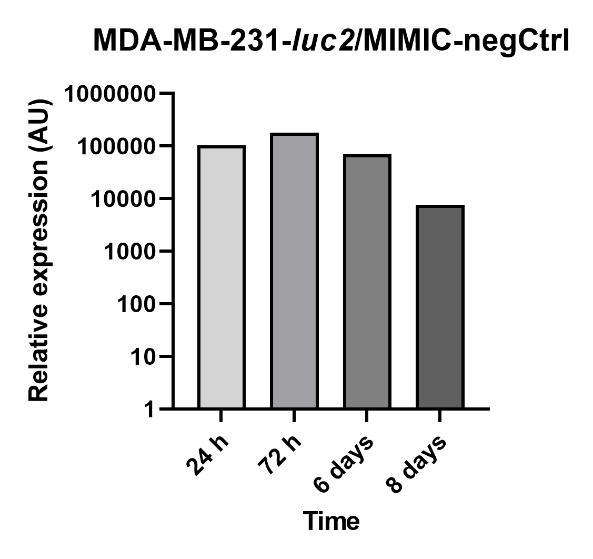

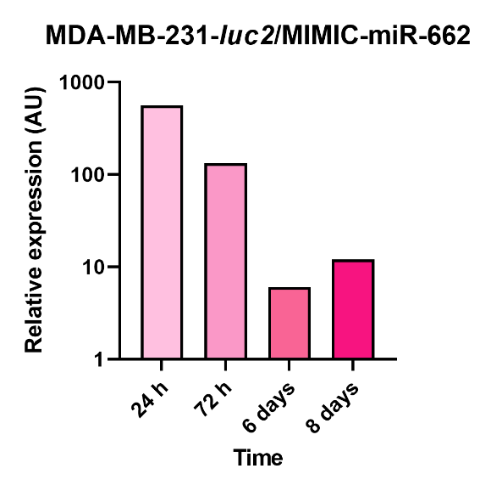


D

**
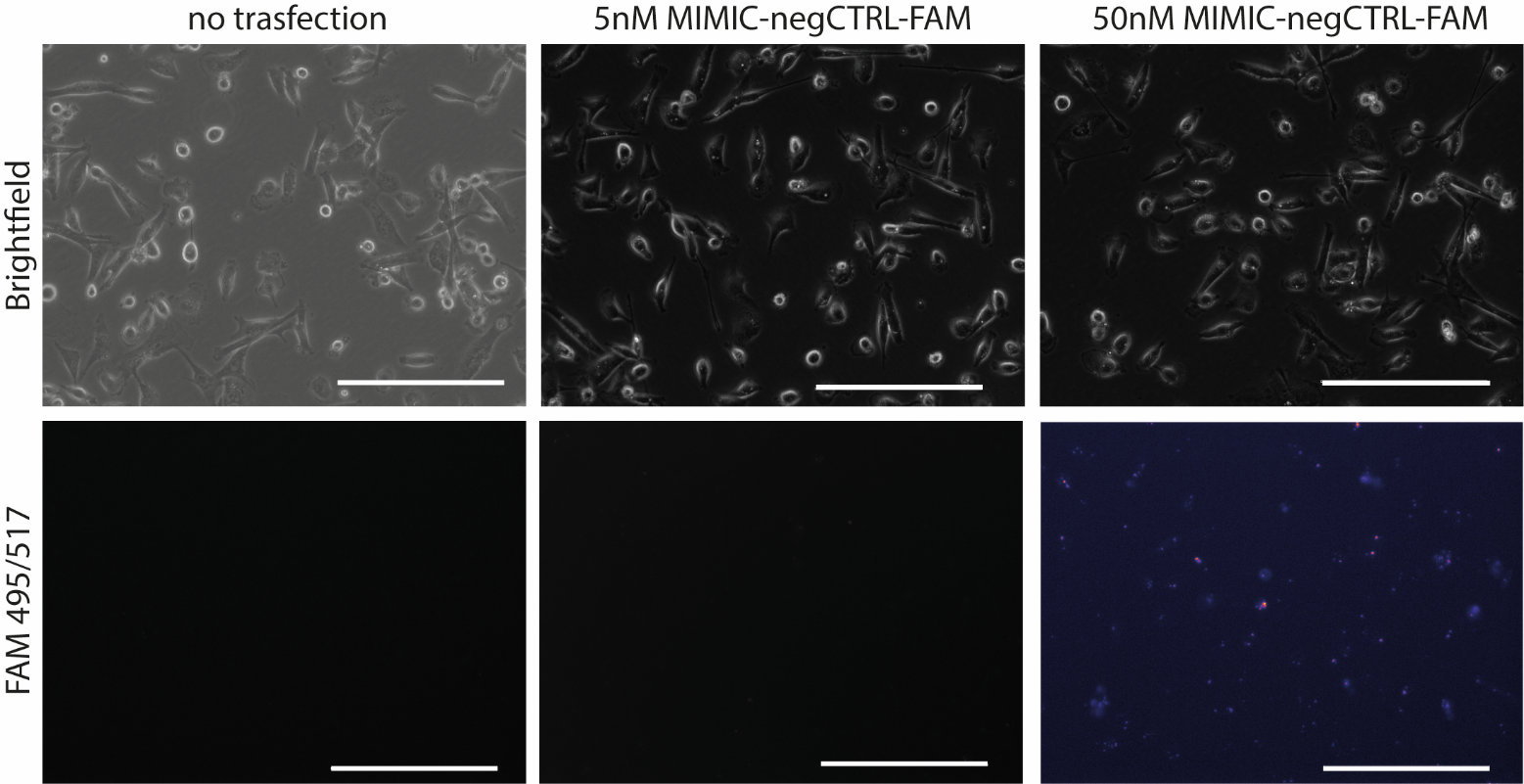
**

A

**Figure S2**

Gates for MDA-MB-231-*luc2* cells transfected with MIMIC-negCtrl or MIMIC-miR-662 were established using ALDEFLUOR-stained DEAB-treated (+DEAB) cells as negative controls (<0.5% ALDH+ cells) (**PANEL A, B**) for each sample. Percentage of ALDH+ cells (in green) were obtained on viable and single cell population (in blue) (**PANEL C, D**).


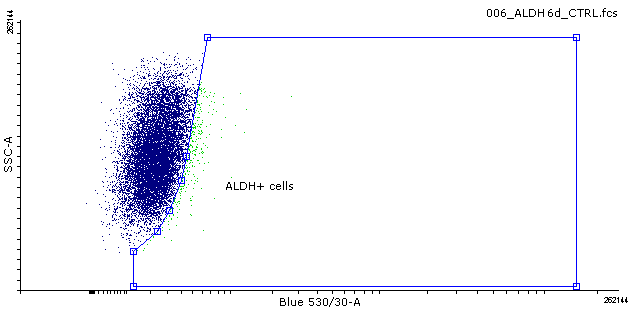


C


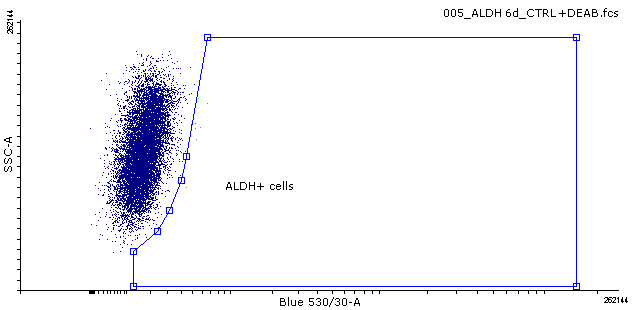


A


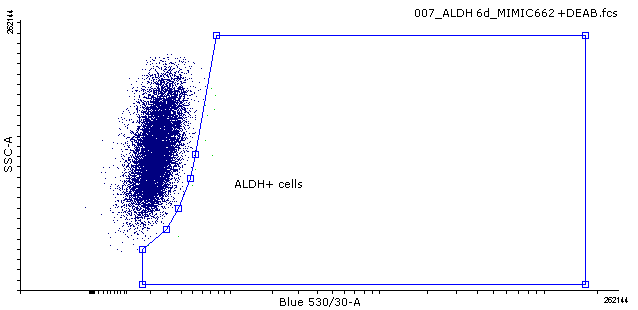


B


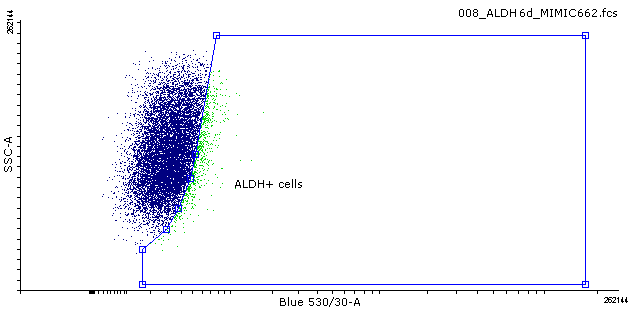


D

**Figure S3**

Expression levels of miR-662 in the serum of breast cancer patients according to zoledronic acid (ZA) treatment. Patients who received or did not receive the treatment are identified as ZA+ or ZA-, respectively. All patients (NOMET+MET) or their subgroups (MET, NOMET, BONE MET) have been taken into consideration for this analysis.


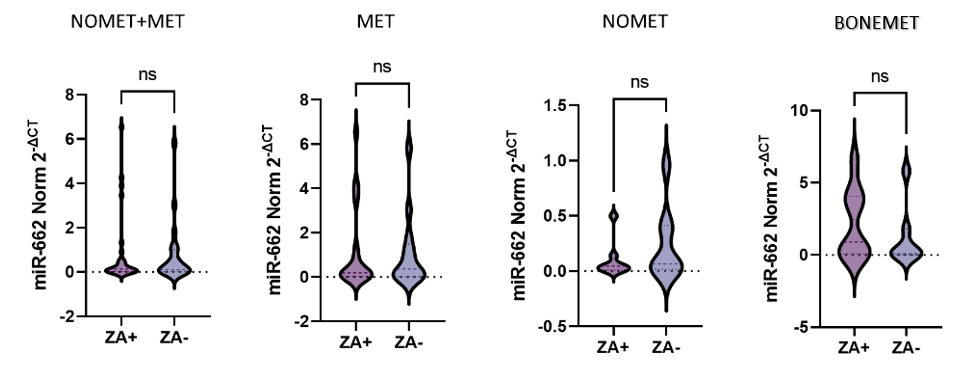


**Figure S4**

Stable overexpression of miR-662 in NW1 cells (LENTI-662-GFP+) in comparison to their relative control (LENTI-Ctrl-GFP+).

**
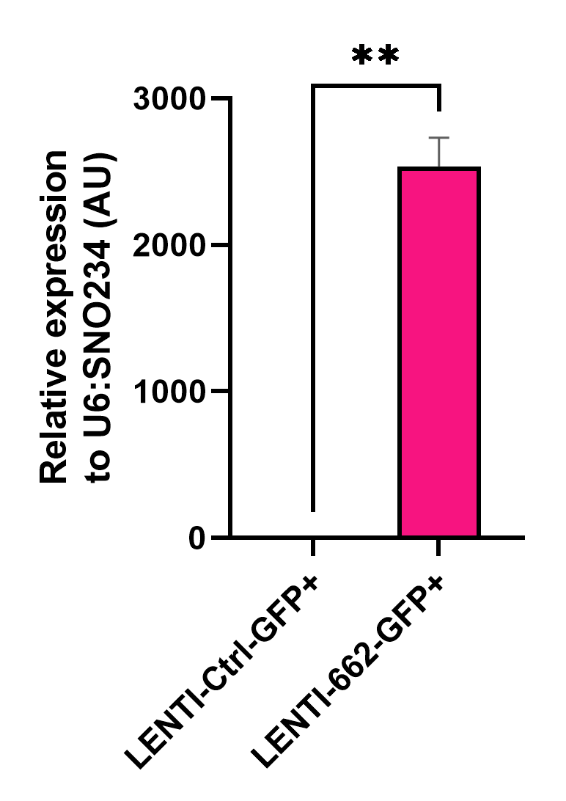
**

**Figure S5**

MiR-662-overexpressing NW1 cells (LENTI-miR-662) showed an increase in cell proliferation compared to control (LENTI-negCTRL) **(A).** MiR-662-overexpressing NW1 cells (LENTI-miR-662) showed an increase in cell migration across the membrane compared to control (LENTI-negCTRL). Migrated cells have been manually counted on ROI (1-3) representative of the entire surface of the membrane **(B)**. Means of three independent experiments ± SEM were shown for all experiments, *p<.05.


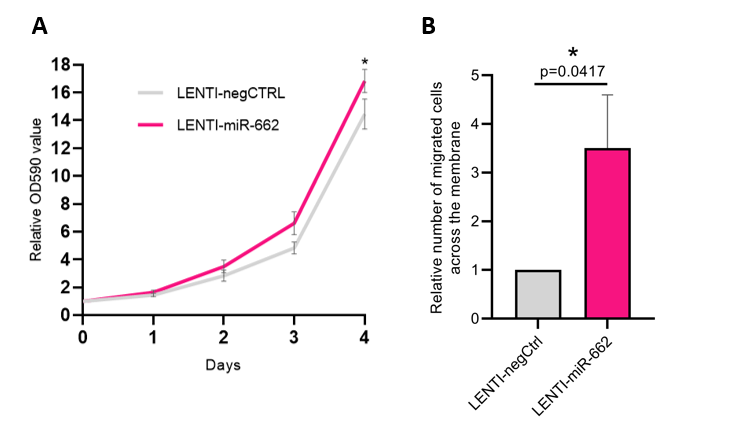


**Figure S6**

Representative images of BLI imaging, microCT scan and TRAP-stained tibia section of mice belonging to the experimental (NW1/LENTI-662) or control (LENTI-Ctrl-GFP+) group. Arrows indicate macro-metastasis in mice.


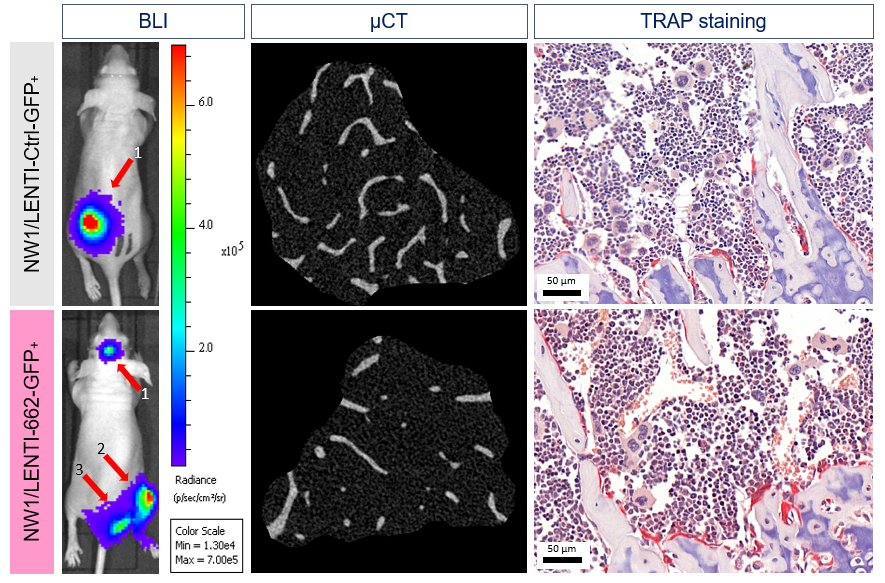


**Figure S7**

Number of osteoclast (cells/mm) evaluated in free-tumour tibiae sections of mice injected with NW1/LENTI-662-GFP+ cells or control cells.

**
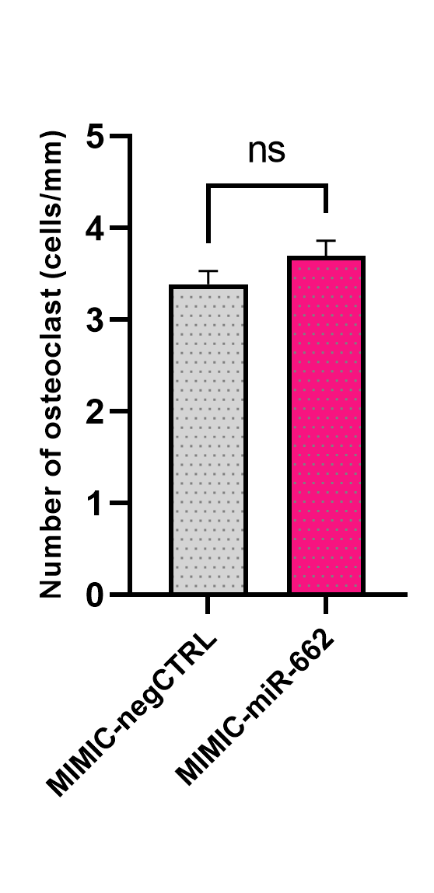
**

**Figure S8**

**
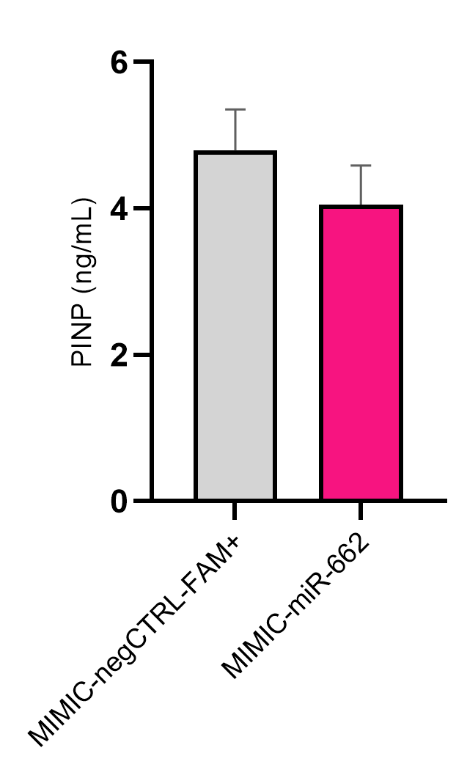
**Serum concentrations for P1NP in the experimental and control group, with no difference between two groups.

**Figure S9**

Representative images (2.5, 10, 20 X) of the osteoclastogenesis assay in presence of conditioned medium (CM) from NW1 cells transfected with MIMIC-miR-662 or MIMIC-negCTRL. Images were taken at day 7, after TRAP staining to specifically identify osteoclasts (in purple). Multinucleated (>3 nuclei) osteoclasts have been counted as mature osteoclasts in 3 independent wells of each experiment performed in triplicates. Scale bars: 1 mm (2.5 X), 500 μm (10 X), 100 μm (20 X).

**
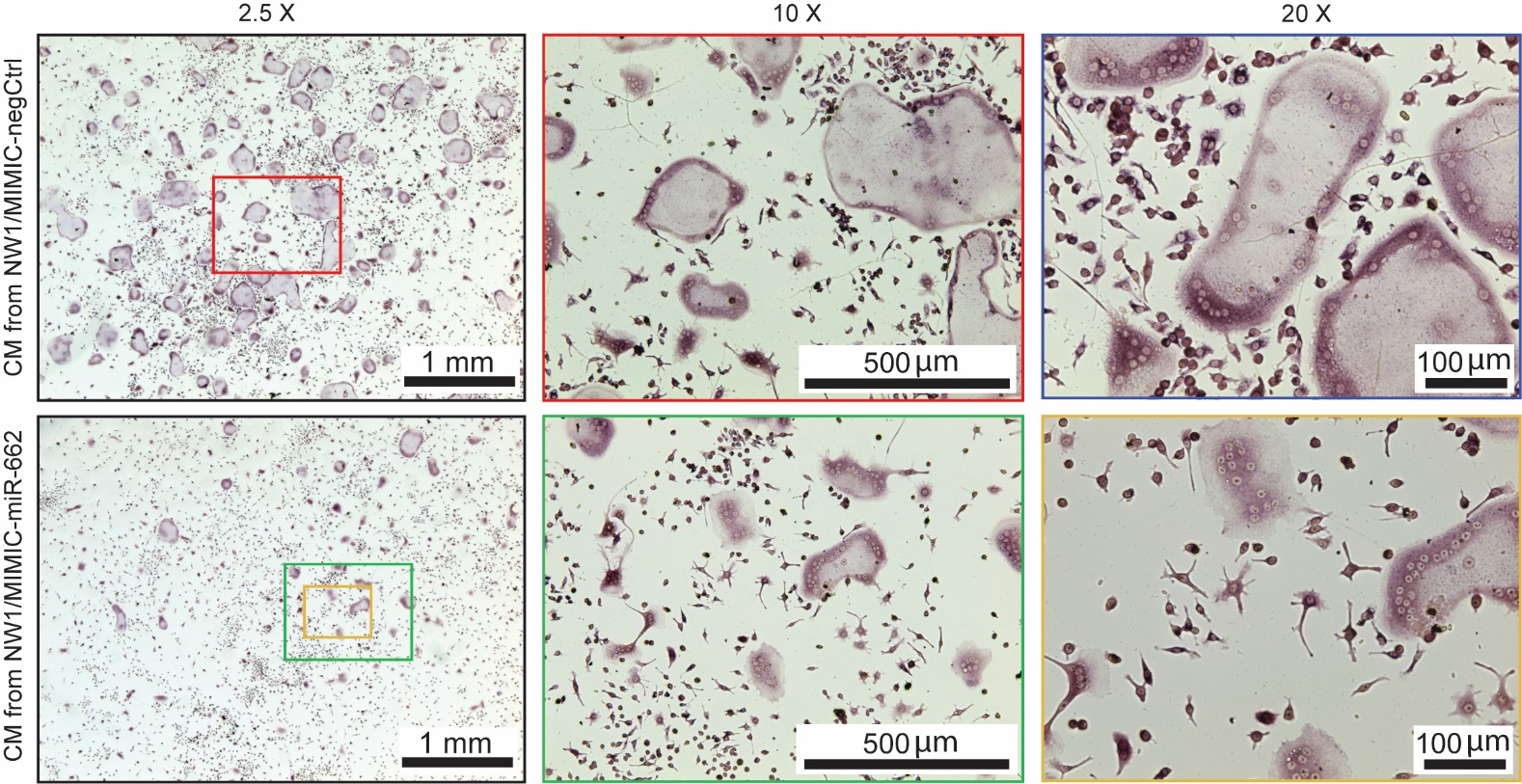
**

**Figure S10**

Representative images (10 X) of the osteoclastogenesis assay in presence of conditioned medium (CM) from NW1 cells transfected with MIMIC-miR-662 or MIMIC-negCTRL. Images were taken at day 12, after TRAP staining to specifically identify osteoclasts (in purple). Multinucleated (>3 nuclei) osteoclasts have been counted as mature osteoclasts in 3 independent wells of each experiment performed in triplicates. Scale bars: 100 μm.

**
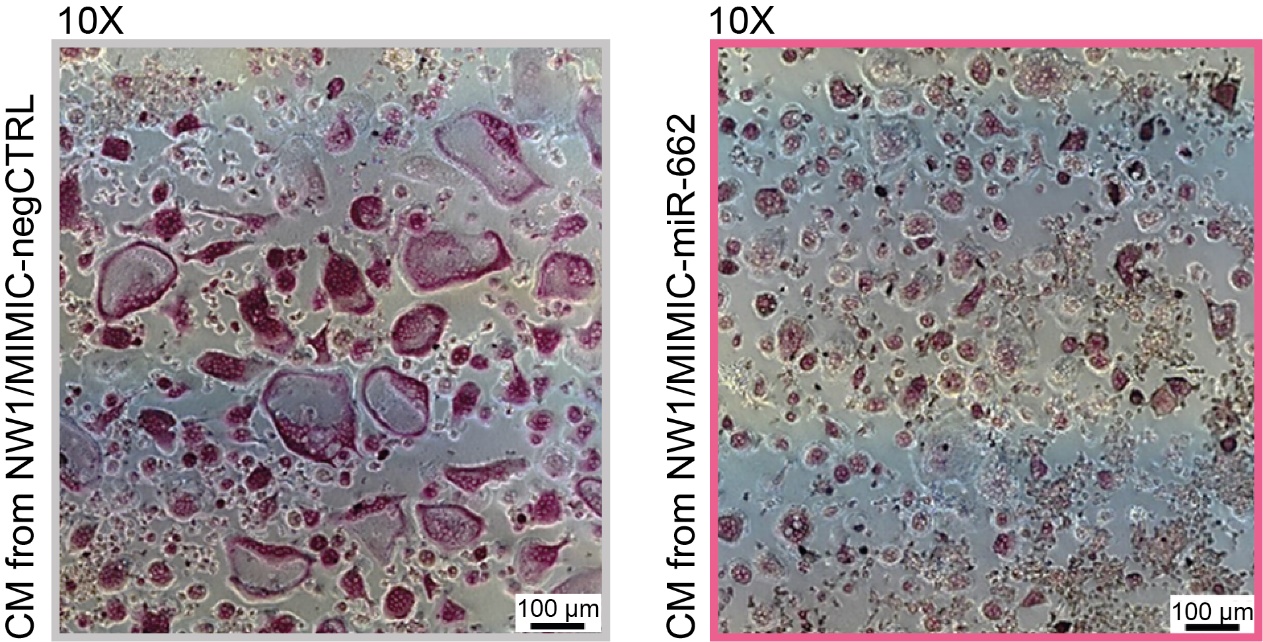
**

**Figure S11**


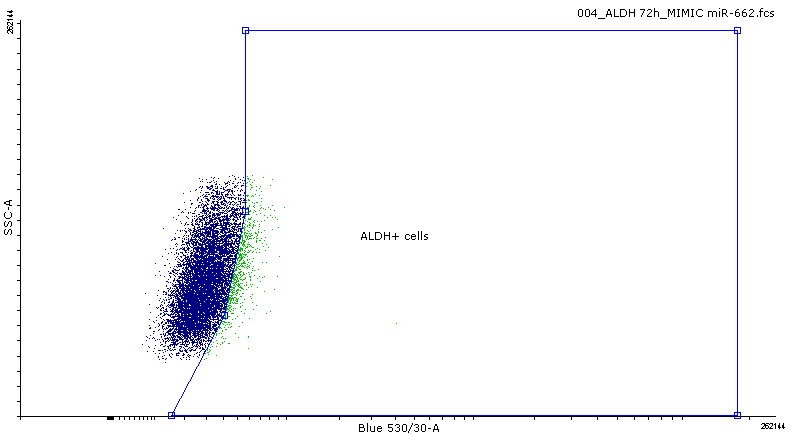

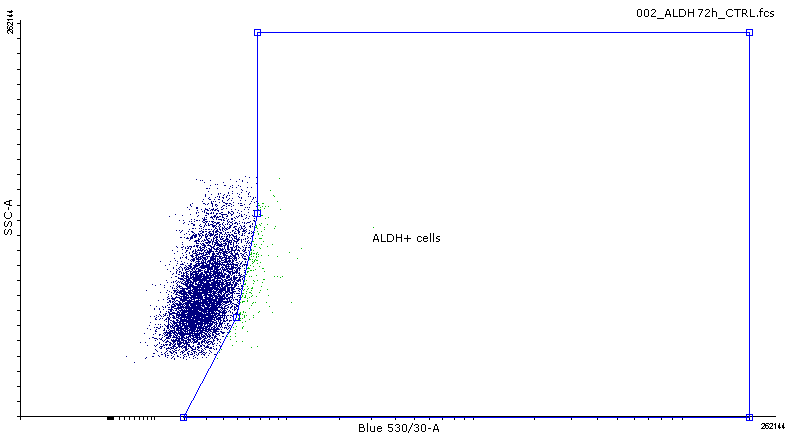
MDA-MB-231-*luc2/*MIMIC-negCtrl and MDA-MB-231-*luc2/*MIMIC-miR-662 were grown in a monolayer culture, and cells were collected 72 hour-post transfections and analysed by flow-cytometry. Gates were established as previous described (**Fig. S2**). Relative percentage of ALDH^high^ cells (in green) were obtained on viable and single cell population (in blue) for experimental (on the right) and control samples (on the left). Here, representative results are shown.

**Figure S12**

MDA-MB-231-*luc2/*MIMIC-negCtrl and MDA-MB-231-*luc2/*MIMIC-miR-662 were grown in as tumorspheres (3D organoids), cells were collected 1 week-post transfections and analysed by flow-cytometry. Gates were established as previous described (**Fig. S2**). Relative percentage of ALDH^high^ cells (in green) were obtained on viable and single cell population (in blue) for experimental and control samples. Here, representative results are shown.


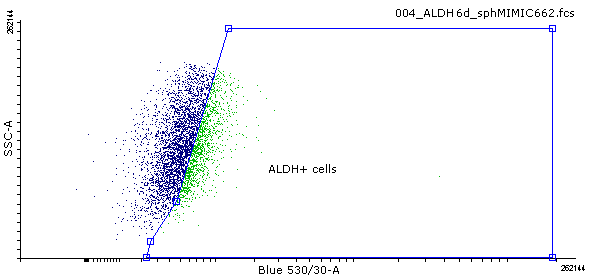

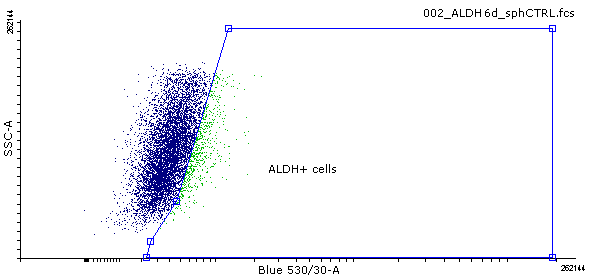


**Figure S13**

Relative expressions (compared to the HKG, b2m) of stemness-related genes (*OCT-04*, *c-MYC*, *CD44, CD24*, *CTNNB1, EZH2*) in MDA-MB-231 cells transfected with MIMIC-negCTRL or MIMIC-miR-662 after 6 days from transfection. Not significative changes are reported for these gene expressions.

**
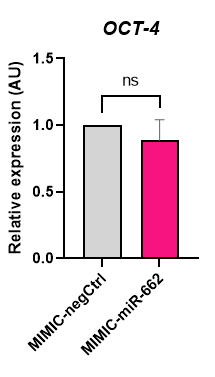

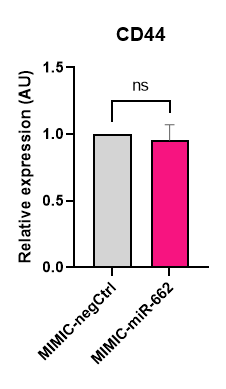

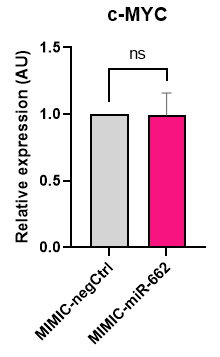
**

**
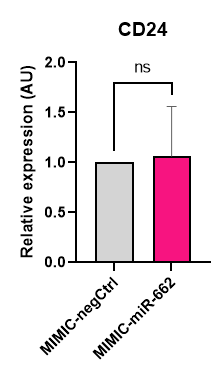

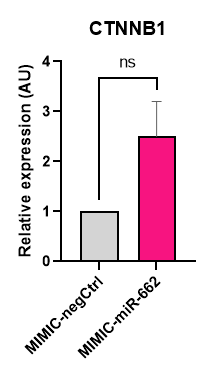
***
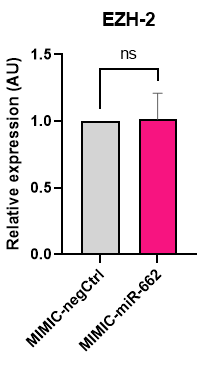
*

**Figure S14**

Gene-concept networks as result of a ClueGo-based analysis using top 200 miR-662 predicted direct targets.


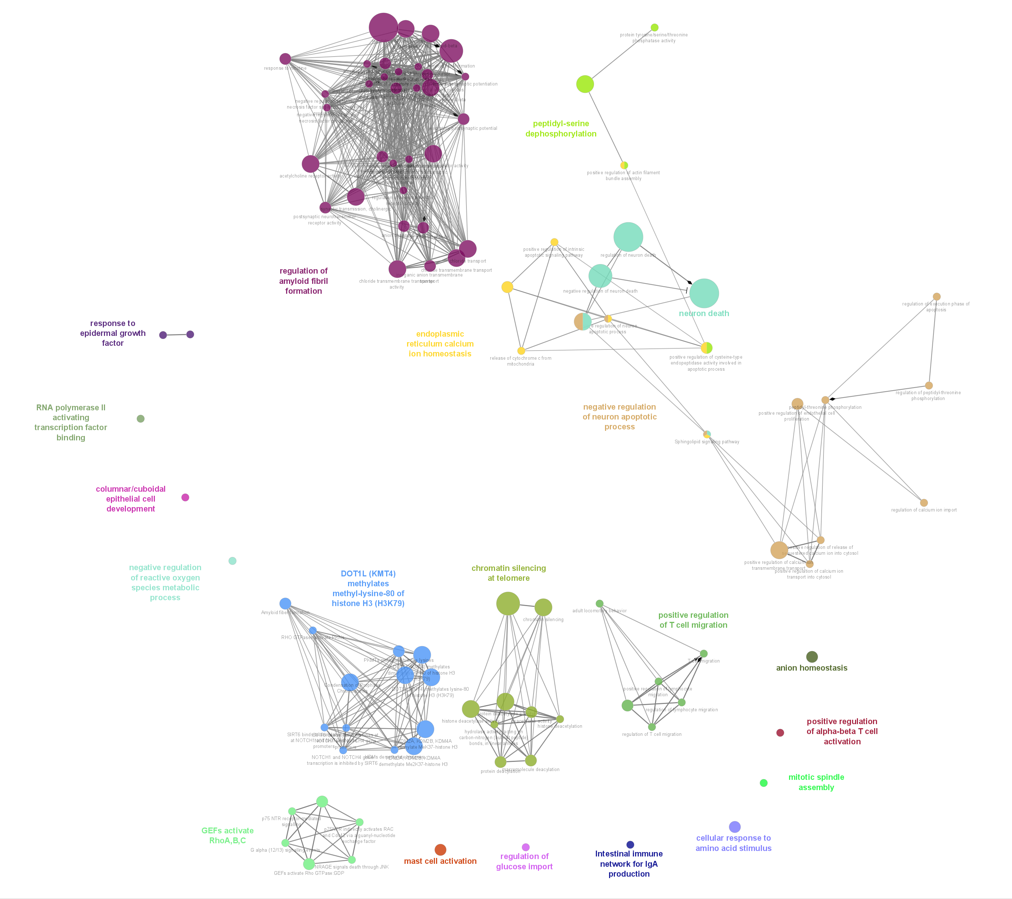


**Figure S15**

Table showing the percentage and number of genes associated to each gene network as results of the ClueGo-based analysis using top 200 miR-662 predicted direct targets.

**
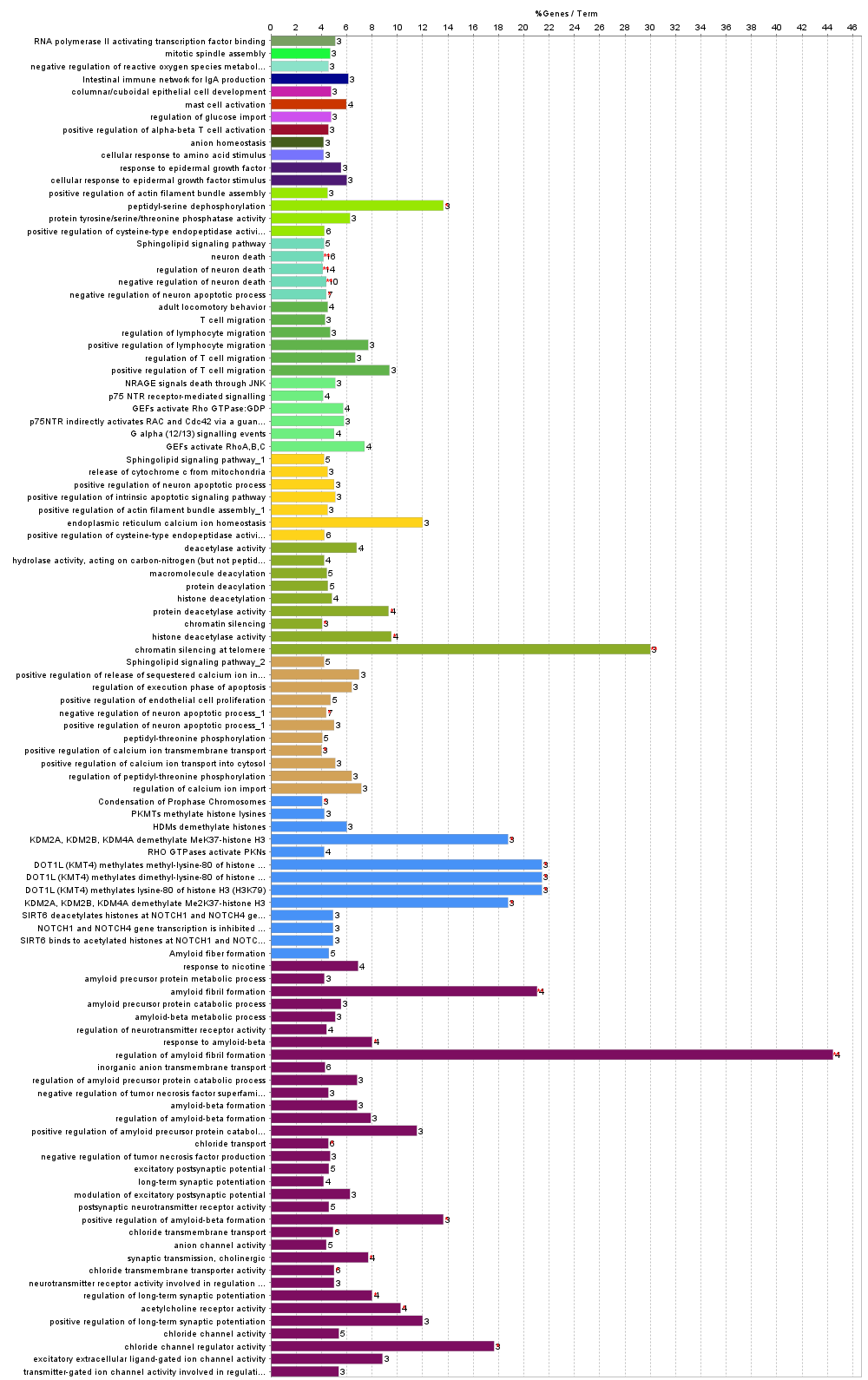
**

**Figure S16**

Volcano plot of genes that resulted modulated (down- or up-regulated) 36 hours post-transfection in MDA-MB-231-*luc2*-NW1/MIMIC-miR-662 (n=3) in comparison to relative control (n=3). Modulated transcripts are labeled in the plot.


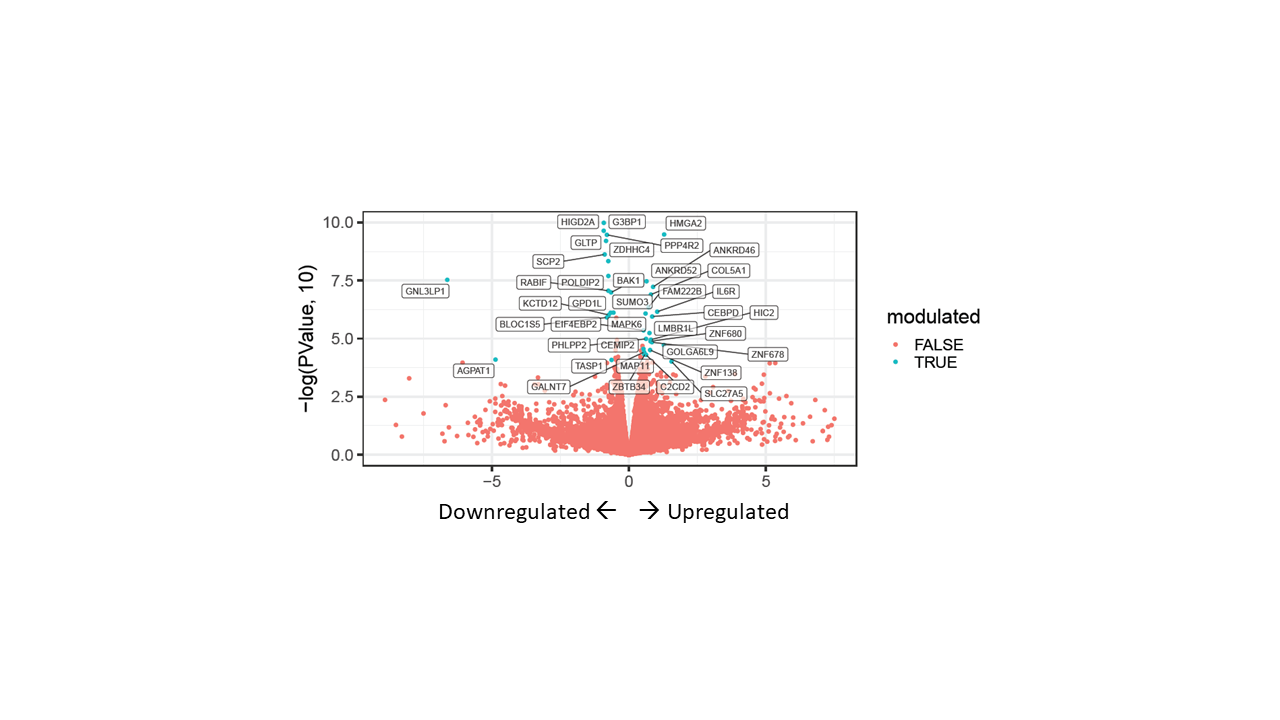


**Table S1**

Detailed list of the 646 human miRNAs targeted in TLDA plates (plate A, plate B), here reported in alphabetic number. In addition, ath-miR159a was quantified in both plates, and further used as process control.

**Table S2**

CCLE database information (Accessed May, 2020) on miR-662 expression values (LOG2 Read Count) in BC cell lines. BC cells lines were subdivided based on their BC subtype (Lum A, Lum B, HER+, TN).

**Table S3**

Raw data from TLDA miRNA screening (code, CTs, 2^-ΔCT^), and group comparison between BC patients with different relapse status (MET vs NOMET, BONEMET vs NOMET, SOFTMET vs NOMET). MiR-662 values have been highlighted in yellow.

**Table S4**

Complete lists of miRNAs resulted significative for ROC analysis (AUC>.70, asymptotic signature<.05) in predict metastasis recurrence (MET vs NOMET, BONEMET vs NOMET, SOFTMET vs NOMET). Only for MET vs NOMET comparison, we included values with AUC>.67 (highlighted in pale yellow), since some of these miRNAs are then significative in the other two comparisons. Lower and upper bounds for confidence interval is shown.

**Table S5**

Summary of GSEA analysis results on RNA-seq data.

**Table S6**

Complete list of miR-662 predicted target obtained using TargetScanHuman 7.0 software. Top 200 predicted targets (arbitrary threshold) were used to perform a ClueGo-based analysis entering ‘GO Biological Process’, ‘GO molecular functions’, ‘KEGG’, ‘Reac Pathways’ and ‘Reac Reactions’ as databases. Detailed results are here shown.
