# Supplementary material for: MiR-662 is associated with metastatic relapse in early-stage breast cancer and promotes metastasis by stimulating cancer cell stemness
Source: Br J Cancer. 2023 Jul 13;129(5):754–71. doi: 10.1038/s41416-023-02340-9 (PMC10449914; doi:10.1038/s41416-023-02340-9)
Supplement: Supplementary file 2 — Supplementary Table 1 [file 41416_2023_2340_MOESM2_ESM.docx]

**Table S1**

|  | **Target Name** | **Target Name** | **Target Name** | **Target Name** | **Target Name** | **Target Name** | **Target Name** |
| --- | --- | --- | --- | --- | --- | --- | --- |
|  | **A** | **B** | **C** | **D** | **E** | **F** | **G** |
| **1** | hsa-let-7a-3p | hsa-miR-1305 | hsa-miR-19b-3p | hsa-miR-330-5p | hsa-miR-492 | hsa-miR-562 | hsa-miR-765 |
| **2** | hsa-let-7a-5p | hsa-miR-130a-3p | hsa-miR-200a-3p | hsa-miR-331-3p | hsa-miR-493-3p | hsa-miR-564 | hsa-miR-769-3p |
| **3** | hsa-let-7b-3p | hsa-miR-130b-3p | hsa-miR-200b-3p | hsa-miR-331-5p | hsa-miR-494-3p | hsa-miR-566 | hsa-miR-769-5p |
| **4** | hsa-let-7b-5p | hsa-miR-130b-5p | hsa-miR-200b-5p | hsa-miR-335-3p | hsa-miR-495-3p | hsa-miR-567 | hsa-miR-770-5p |
| **5** | hsa-let-7c-5p | hsa-miR-132-3p | hsa-miR-200c-3p | hsa-miR-335-5p | hsa-miR-496 | hsa-miR-569 | hsa-miR-873-5p |
| **6** | hsa-let-7d-3p | hsa-miR-1324 | hsa-miR-202-3p | hsa-miR-337-3p | hsa-miR-497-5p | hsa-miR-570-3p | hsa-miR-874-3p |
| **7** | hsa-let-7d-5p | hsa-miR-132-5p | hsa-miR-203a-3p | hsa-miR-337-5p | hsa-miR-499a-5p | hsa-miR-571 | hsa-miR-875-3p |
| **8** | hsa-let-7e-3p | hsa-miR-133a-3p | hsa-miR-204-5p | hsa-miR-338-3p | hsa-miR-500a-3p | hsa-miR-572 | hsa-miR-875-5p |
| **9** | hsa-let-7e-5p | hsa-miR-133b | hsa-miR-205-5p | hsa-miR-338-5P | hsa-miR-500a-5p | hsa-miR-573 | hsa-miR-876-3p |
| **10** | hsa-let-7f-1-3p | hsa-miR-134-5p | hsa-miR-206 | hsa-miR-339-3p | hsa-miR-501-3p | hsa-miR-574-3p | hsa-miR-876-5p |
| **11** | hsa-let-7f-2-3p | hsa-miR-135a-5p | hsa-miR-208a-3p | hsa-miR-339-5p | hsa-miR-501-5p | hsa-miR-575 | hsa-miR-885-5p |
| **12** | hsa-let-7f-5p | hsa-miR-135b-3p | hsa-miR-208b-3p | hsa-miR-33a-3p | hsa-miR-502-3p | hsa-miR-576-3p | hsa-miR-886-3p |
| **13** | hsa-let-7g-3p | hsa-miR-135b-5p | hsa-miR-20a-3p | hsa-miR-33a-5p | hsa-miR-502-5p | hsa-miR-576-5p | hsa-miR-886-5p |
| **14** | hsa-let-7g-5p | hsa-miR-136-3p | hsa-miR-20a-5p | hsa-miR-33b-5p | hsa-miR-503-5p | hsa-miR-577 | hsa-miR-888-3p |
| **15** | hsa-let-7i-3p | hsa-miR-136-5p | hsa-miR-20b-3p | hsa-miR-340-3p | hsa-miR-504-5p | hsa-miR-578 | hsa-miR-888-5p |
| **16** | hsa-miR-100-3p | hsa-miR-137 | hsa-miR-20b-5p | hsa-miR-340-5p | hsa-miR-505-3p | hsa-miR-579-3p | hsa-miR-889-3p |
| **17** | hsa-miR-100-5p | hsa-miR-138-2-3p | hsa-miR-210-3p | hsa-miR-342-3p | hsa-miR-505-5p | hsa-miR-580-3p | hsa-miR-890 |
| **18** | hsa-miR-101-3p | hsa-miR-138-5p | hsa-miR-211-5p | hsa-miR-342-5p | hsa-miR-506-3p | hsa-miR-581 | hsa-miR-891a-5p |
| **19** | hsa-miR-101-5p | hsa-miR-139-3p | hsa-miR-212-3p | hsa-miR-345-5p | hsa-miR-508-3p | hsa-miR-582-3p | hsa-miR-892b |
| **20** | hsa-miR-103a-3p | hsa-miR-139-5p | hsa-miR-21-3p | hsa-miR-346 | hsa-miR-509-3-5p | hsa-miR-582-5p | hsa-miR-921 |
| **21** | hsa-miR-105-3p | hsa-miR-1-3p | hsa-miR-214-3p | hsa-miR-34a-3p | hsa-miR-509-5p | hsa-miR-584-5p | hsa-miR-922 |
| **22** | hsa-miR-105-5p | hsa-miR-140 | hsa-miR-214-5p | hsa-miR-34a-5p | hsa-miR-510-5p | hsa-miR-586 | hsa-miR-924 |
| **23** | hsa-miR-106a-5p | hsa-miR-140-3p | hsa-miR-215-5p | hsa-miR-34b-3p | hsa-miR-511-5p | hsa-miR-587 | hsa-miR-92a-1-5p |
| **24** | hsa-miR-106b-3p | hsa-miR-141 | hsa-miR-21-5p | hsa-miR-34b-5p | hsa-miR-512-3p | hsa-miR-589-3p | hsa-miR-92a-3p |
| **25** | hsa-miR-106b-5p | hsa-miR-141-5p | hsa-miR-216a-5p | hsa-miR-34c-5p | hsa-miR-512-5p | hsa-miR-589-5p | hsa-miR-92b-5p |
| **26** | hsa-miR-107 | hsa-miR-142-3p | hsa-miR-216b-5p | hsa-miR-361-3p | hsa-miR-515-3p | hsa-miR-590-3p | hsa-miR-933 |
| **27** | hsa-miR-10a-3p | hsa-miR-142-5p | hsa-miR-218-1-3p | hsa-miR-361-5p | hsa-miR-515-5p | hsa-miR-590-5p | hsa-miR-93-3p |
| **28** | hsa-miR-10a-5p | hsa-miR-143 | hsa-miR-218-5p | hsa-miR-362-3p | hsa-miR-516-3p | hsa-miR-591 | hsa-miR-935 |
| **29** | hsa-miR-10b-3p | hsa-miR-143-5p | hsa-miR-219a-1-3p | hsa-miR-362-5p | hsa-miR-516a-5p | hsa-miR-592 | hsa-miR-93-5p |
| **30** | hsa-miR-10b-5p | hsa-miR-144-3p | hsa-miR-219a-2-3p | hsa-miR-363-3p | hsa-miR-516b-5p | hsa-miR-593-3p | hsa-miR-937-3p |
| **31** | hsa-miR-1179 | hsa-miR-144-5p | hsa-miR-219a-5p | hsa-miR-363-5p | hsa-miR-517-5p | hsa-miR-593-5p | hsa-miR-938 |
| **32** | hsa-miR-1180-3p | hsa-miR-145 | hsa-miR-220 | hsa-miR-365a-3p | hsa-miR-517a-3p | hsa-miR-595 | hsa-miR-939-5p |
| **33** | hsa-miR-1183 | hsa-miR-145-3p | hsa-miR-220b | hsa-miR-367-3p | hsa-miR-517c-3p | hsa-miR-596 | hsa-miR-9-3p |
| **34** | hsa-miR-1184 | hsa-miR-146a | hsa-miR-221-3p | hsa-miR-367-5p | hsa-miR-518a-3p | hsa-miR-597-5p | hsa-miR-941 |
| **35** | hsa-miR-1197 | hsa-miR-146b | hsa-miR-221-5p | hsa-miR-369-3p | hsa-miR-518a-5p | hsa-miR-598-3p | hsa-miR-942-5p |
| **36** | hsa-miR-1201 | hsa-miR-146b-3p | hsa-miR-222-3p | hsa-miR-369-5p | hsa-miR-518b | hsa-miR-599 | hsa-miR-943 |
| **37** | hsa-miR-1203 | hsa-miR-147a | hsa-miR-222-5p | hsa-miR-370-3p | hsa-miR-518d-3p | hsa-miR-600 | hsa-miR-944 |
| **38** | hsa-miR-1208 | hsa-miR-147b | hsa-miR-223-5p | hsa-miR-371a-3p | hsa-miR-518d-5p | hsa-miR-601 | hsa-miR-95-3p |
| **39** | hsa-miR-122-3p | hsa-miR-148a-3p | hsa-miR-22-3p | hsa-miR-372-3p | hsa-miR-518e# | hsa-miR-603 | hsa-miR-9-5p |
| **40** | hsa-miR-1225-3p | hsa-miR-148a-5p | hsa-miR-224-5p | hsa-miR-373-3p | hsa-miR-518e-3p | hsa-miR-604 | hsa-miR-96-3p |
| **41** | hsa-miR-122-5p | hsa-miR-148b-3p | hsa-miR-22-5p | hsa-miR-374a-3p | hsa-miR-518f-3p | hsa-miR-605-5p | hsa-miR-96-5p |
| **42** | hsa-miR-1226-5p | hsa-miR-148b-5p | hsa-miR-23a-3p | hsa-miR-374a-5p | hsa-miR-518f-5p | hsa-miR-606 | hsa-miR-98-5p |
| **43** | hsa-miR-1227-3p | hsa-miR-149-3p | hsa-miR-23a-5p | hsa-miR-374b-3p | hsa-miR-519a-3p | hsa-miR-614 | hsa-miR-99a-3p |
| **44** | hsa-miR-1228-5p | hsa-miR-149-5p | hsa-miR-23b-3p | hsa-miR-374b-5p | hsa-miR-519b-3p | hsa-miR-615-3p | hsa-miR-99a-5p |
| **45** | hsa-miR-1233-3p | hsa-miR-150-5p | hsa-miR-23b-5p | hsa-miR-375 | hsa-miR-519c-3p | hsa-miR-615-5p | hsa-miR-99b-3p |
| **46** | hsa-miR-1238-3p | hsa-miR-151a-5p | hsa-miR-24-1-5p | hsa-miR-376a-3p | hsa-miR-519d-3p | hsa-miR-616-3p | hsa-miR-99b-5p |
| **47** | hsa-miR-1243 | hsa-miR-152-3p | hsa-miR-24-2-5p | hsa-miR-376a-5p | hsa-miR-519e-3p | hsa-miR-616-5p |  |
| **48** | hsa-miR-124-3p | hsa-miR-153-3p | hsa-miR-24-3p | hsa-miR-376b-3p | hsa-miR-519e-5p | hsa-miR-617 |  |
| **49** | hsa-miR-1244 | hsa-miR-154-3p | hsa-miR-25-3p | hsa-miR-376c-3p | hsa-miR-520a-3p | hsa-miR-618 |  |
| **50** | hsa-miR-124-5p | hsa-miR-154-5p | hsa-miR-25-5p | hsa-miR-377-3p | hsa-miR-520a-5p | hsa-miR-621 |  |
| **51** | hsa-miR-1247-5p | hsa-miR-155-5p | hsa-miR-26a-1-3p | hsa-miR-377-5p | hsa-miR-520b | hsa-miR-622 |  |
| **52** | hsa-miR-1248 | hsa-miR-15a-3p | hsa-miR-26a-2-3p | hsa-miR-378 | hsa-miR-520c-3p | hsa-miR-623 |  |
| **53** | hsa-miR-1249-3p | hsa-miR-15a-5p | hsa-miR-26a-5p | hsa-miR-378a-5p | hsa-miR-520d-3p | hsa-miR-624-3p |  |
| **54** | hsa-miR-1250-5p | hsa-miR-15b-3p | hsa-miR-26b-3p | hsa-miR-379-5p | hsa-miR-520f-3p | hsa-miR-624-5p |  |
| **55** | hsa-miR-1252-5p | hsa-miR-15b-5p | hsa-miR-26b-5p | hsa-miR-380-3p | hsa-miR-520g-3p | hsa-miR-625-3p |  |
| **56** | hsa-miR-1253 | hsa-miR-16-1-3p | hsa-miR-27a-3p | hsa-miR-380-5p | hsa-miR-520h | hsa-miR-625-5p |  |
| **57** | hsa-miR-1254 | hsa-miR-16-5p | hsa-miR-27a-5p | hsa-miR-381-3p | hsa-miR-521 | hsa-miR-627-5p |  |
| **58** | hsa-miR-1255a | hsa-miR-17-3p | hsa-miR-27b-3p | hsa-miR-382-5p | hsa-miR-522-3p | hsa-miR-628-3p |  |
| **59** | hsa-miR-1255b-5p | hsa-miR-17-5p | hsa-miR-27b-5p | hsa-miR-383-5p | hsa-miR-523-3p | hsa-miR-628-5p |  |
| **60** | hsa-miR-1256 | hsa-miR-181a-2-3p | hsa-miR-28-3p | hsa-miR-409-3p | hsa-miR-524-3p | hsa-miR-629-3p |  |
| **61** | hsa-miR-1259 | hsa-miR-181a-3p | hsa-miR-28-5p | hsa-miR-409-5p | hsa-miR-524-5p | hsa-miR-629-5p |  |
| **62** | hsa-miR-125a-3p | hsa-miR-181a-5p | hsa-miR-296-3p | hsa-miR-410-3p | hsa-miR-525-3p | hsa-miR-630 |  |
| **63** | hsa-miR-125a-5p | hsa-miR-181c-3p | hsa-miR-296-5p | hsa-miR-411-3p | hsa-miR-525-5p | hsa-miR-633 |  |
| **64** | hsa-miR-125b-1-3p | hsa-miR-181c-5p | hsa-miR-298 | hsa-miR-411-5p | hsa-miR-526b-5p | hsa-miR-635 |  |
| **65** | hsa-miR-125b-2-3p | hsa-miR-182-3p | hsa-miR-299-5p | hsa-miR-412-3p | hsa-miR-532-3p | hsa-miR-636 |  |
| **66** | hsa-miR-125b-5p | hsa-miR-1825 | hsa-miR-29a-3p | hsa-miR-422a | hsa-miR-532-5p | hsa-miR-638 |  |
| **67** | hsa-miR-1260a | hsa-miR-182-5p | hsa-miR-29a-5p | hsa-miR-423-5p | hsa-miR-539-5p | hsa-miR-639 |  |
| **68** | hsa-miR-1262 | hsa-miR-183-3p | hsa-miR-29b-1-5p | hsa-miR-424-3p | hsa-miR-541-3p | hsa-miR-640 |  |
| **69** | hsa-miR-126-3p | hsa-miR-183-5p | hsa-miR-29b-2-5p | hsa-miR-424-5p | hsa-miR-541-5p | hsa-miR-641 |  |
| **70** | hsa-miR-1265 | hsa-miR-184 | hsa-miR-29b-3p | hsa-miR-425-3p | hsa-miR-542-3p | hsa-miR-642a-5p |  |
| **71** | hsa-miR-126-5p | hsa-miR-185-3p | hsa-miR-29c-3p | hsa-miR-425-5p | hsa-miR-542-5p | hsa-miR-643 |  |
| **72** | hsa-miR-1267 | hsa-miR-185-5p | hsa-miR-29c-5p | hsa-miR-429 | hsa-miR-543 | hsa-miR-644a |  |
| **73** | hsa-miR-1269a | hsa-miR-186-3p | hsa-miR-301a-3p | hsa-miR-431-3p | hsa-miR-544a | hsa-miR-645 |  |
| **74** | hsa-miR-1270 | hsa-miR-186-5p | hsa-miR-301b-3p | hsa-miR-431-5p | hsa-miR-545-3p | hsa-miR-646 |  |
| **75** | hsa-miR-1271-5p | hsa-miR-187-3p | hsa-miR-302a-3p | hsa-miR-432-3p | hsa-miR-545-5p | hsa-miR-648 |  |
| **76** | hsa-miR-127-3p | hsa-miR-188-3p | hsa-miR-302a-5p | hsa-miR-432-5p | hsa-miR-548a-3p | hsa-miR-649 |  |
| **77** | hsa-miR-1274A | hsa-miR-18a-3p | hsa-miR-302b-3p | hsa-miR-433-3p | hsa-miR-548a-5p | hsa-miR-650 |  |
| **78** | hsa-miR-1274B | hsa-miR-18a-5p | hsa-miR-302c-3p | hsa-miR-449a | hsa-miR-548a-5p | hsa-miR-651-5p |  |
| **79** | hsa-miR-1275 | hsa-miR-18b-5p | hsa-miR-302c-5p | hsa-miR-449b-5p | hsa-miR-548b-3p | hsa-miR-653-5p |  |
| **80** | hsa-miR-1276 | hsa-miR-190a-5p | hsa-miR-302d-3p | hsa-miR-450a-5p | hsa-miR-548b-5p | hsa-miR-654-3p |  |
| **81** | hsa-miR-1282 | hsa-miR-190b | hsa-miR-30a-3p | hsa-miR-450b-3p | hsa-miR-548c-3p | hsa-miR-654-5p |  |
| **82** | hsa-miR-1283 | hsa-miR-191-3p | hsa-miR-30a-5p | hsa-miR-450b-5p | hsa-miR-548d-3p | hsa-miR-655-3p |  |
| **83** | hsa-miR-128-3p | hsa-miR-191-5p | hsa-miR-30b-5p | hsa-miR-451a | hsa-miR-548d-5p | hsa-miR-656-3p |  |
| **84** | hsa-miR-1284 | hsa-miR-192-3p | hsa-miR-30c-5p | hsa-miR-452-5p | hsa-miR-548e-3p | hsa-miR-658 |  |
| **85** | hsa-miR-1285-3p | hsa-miR-192-5p | hsa-miR-30d-3p | hsa-miR-454-3p | hsa-miR-548h-5p | hsa-miR-659-3p |  |
| **86** | hsa-miR-1286 | hsa-miR-193a-3p | hsa-miR-30d-5p | hsa-miR-454-5p | hsa-miR-548j-5p | hsa-miR-660-5p |  |
| **87** | hsa-miR-1288-3p | hsa-miR-193a-5p | hsa-miR-30e-3p | hsa-miR-455-3p | hsa-miR-548m | hsa-miR-661 |  |
| **88** | hsa-miR-1290 | hsa-miR-193b-3p | hsa-miR-31-3p | hsa-miR-455-5p | hsa-miR-548p | hsa-miR-662 |  |
| **89** | hsa-miR-1291 | hsa-miR-193b-5p | hsa-miR-31-5p | hsa-miR-483-3p | hsa-miR-549a | hsa-miR-663b |  |
| **90** | hsa-miR-129-1-3p | hsa-miR-194-5p | hsa-miR-320a | hsa-miR-483-5p | hsa-miR-550a-3p | hsa-miR-664a-3p |  |
| **91** | hsa-miR-129-2-3p | hsa-miR-195-5p | hsa-miR-320b | hsa-miR-484 | hsa-miR-550a-5p | hsa-miR-668-3p |  |
| **92** | hsa-miR-1292-5p | hsa-miR-196b-5p | hsa-miR-323a-3p | hsa-miR-485-3p | hsa-miR-551a | hsa-miR-671-3p |  |
| **93** | hsa-miR-1293 | hsa-miR-197-3p | hsa-miR-324-3p | hsa-miR-486-5p | hsa-miR-551b-3p | hsa-miR-675-5p |  |
| **94** | hsa-miR-1294 | hsa-miR-198 | hsa-miR-324-5p | hsa-miR-487a-3p | hsa-miR-551b-5p | hsa-miR-708-5p |  |
| **95** | hsa-miR-129-5p | hsa-miR-199a-3p | hsa-miR-325 | hsa-miR-487b-3p | hsa-miR-555 | hsa-miR-720 |  |
| **96** | hsa-miR-1296-5p | hsa-miR-199a-5p | hsa-miR-32-5p | hsa-miR-488-5p | hsa-miR-556-3p | hsa-miR-7-2-3p |  |
| **97** | hsa-miR-1298-5p | hsa-miR-199b-5p | hsa-miR-326 | hsa-miR-489-3p | hsa-miR-556-5p | hsa-miR-744-3p |  |
| **98** | hsa-miR-1300 | hsa-miR-19a-3p | hsa-miR-328-3p | hsa-miR-490-3p | hsa-miR-557 | hsa-miR-744-5p |  |
| **99** | hsa-miR-1301-3p | hsa-miR-19a-5p | hsa-miR-329-3p | hsa-miR-491-3p | hsa-miR-559 | hsa-miR-758-3p |  |
| **100** | hsa-miR-1303 | hsa-miR-19b-1-5p | hsa-miR-330-3p | hsa-miR-491-5p | hsa-miR-561-3p | hsa-miR-7-5p |  |
