# Supplementary material for: MiR-662 is associated with metastatic relapse in early-stage breast cancer and promotes metastasis by stimulating cancer cell stemness
Source: Br J Cancer. 2023 Jul 13;129(5):754–71. doi: 10.1038/s41416-023-02340-9 (PMC10449914; doi:10.1038/s41416-023-02340-9)
Supplement: Supplementary file 3 — Supplementary Table 2 [file 41416_2023_2340_MOESM3_ESM.docx]

**Table S2**

| **Lum A** | | **Lum B** | | **HER+** | | **TN** | |
| --- | --- | --- | --- | --- | --- | --- | --- |
| **MDAMB175VII** | 4.022368 | **MDAMB361** | 3.759156 | **HCC202** | 3.914565 | **DU4475** | 4.337711 |
| **T47D** | 3.217231 | **EFM192A** | 3.998196 | **HCC2218** | 4.889474 | **BT20** | 2.550901 |
| **KPL1** | 4.099295 | **BT474** | 3.787641 | **HCC1954** | 4.491212 | **HCC1143** | 3.82273 |
| **CAMA1** | 3.647315 | **UACC812** | 3.545968 | **AU565** | 4.276497 | **HCC1937** | 4.136684 |
| **ZR751** | 4.084064 | **ZR7530** | 4.144862 | **SKBR3** | 4.140779 | **HCC1187** | 3.298658 |
| **MCF7** | 3.203201 |  |  | **HCC1569** | 4.278728 | **HCC70** | 3.942984 |
| **EFM19** | 3.823749 |  |  | **MDAMB453** | 3.776104 | **HCC2157** | 3.404631 |
| **MDAMB415** | 3.874797 |  |  | **UACC893** | 4.380591 | **MDAMB468** | 4.054848 |
| **MDAMB134VI** | 4.357552 |  |  |  |  | **HCC1806** | 3.960697 |
| **BT483** | 3.496974 |  |  |  |  | **CAL148** | 4.218781 |
| **HCC1428** | 4.444932 |  |  |  |  | **HCC1599** | 4.65019 |
|  |  |  |  |  |  | **HCC38** | 3.496973581 |
|  |  |  |  |  |  | **MDAMB231** | 3.606442228 |
|  |  |  |  |  |  | **HS578T** | 3.469885976 |
|  |  |  |  |  |  | **HCC1395** | 3.534808661 |
|  |  |  |  |  |  | **MDAMB157** | 3.513490746 |
|  |  |  |  |  |  | **BT549** | 3.609991295 |
|  |  |  |  |  |  | **CAL851** | 3.665620164 |
|  |  |  |  |  |  | **CAL51** | 2.871843649 |
|  |  |  |  |  |  | **CAL120** | 3.925999419 |
