# Supplementary material for: MiR-662 is associated with metastatic relapse in early-stage breast cancer and promotes metastasis by stimulating cancer cell stemness
Source: Br J Cancer. 2023 Jul 13;129(5):754–71. doi: 10.1038/s41416-023-02340-9 (PMC10449914; doi:10.1038/s41416-023-02340-9)
Supplement: Supplementary file 5 — Supplementary Table 4 [file 41416_2023_2340_MOESM5_ESM.docx]

**Table S4**

|  | **MET vs NOMET** | | | | **NOMET vs MET** | | | | | |
| --- | --- | --- | --- | --- | --- | --- | --- | --- | --- | --- |
|  |  | | **Asymptotic 95% Confidence Interval** | |  | | | **Asymptotic 95% Confidence Interval** | | |
| **miRNA ID** | **Area** | **Asymptotic Signature** | **Lower Bound** | **Upper Bound** | | **Area** | **Asymptotic Signature** | | **Lower Bound** | **Upper Bound** |
| **miR-708-5p** | 0.742 | 0.01 | 0.588 | 0.896 | | 0.258 | 0.01 | | 0.104 | 0.412 |
| **miR-30a-5p** | 0.731 | 0.01 | 0.58 | 0.883 | | 0.269 | 0.01 | | 0.117 | 0.42 |
| **miR-30d-5p** | 0.722 | 0.01 | 0.573 | 0.871 | | 0.278 | 0.01 | | 0.129 | 0.427 |
| **miR-1253** | 0.718 | 0.02 | 0.565 | 0.871 | | 0.282 | 0.02 | | 0.129 | 0.435 |
| **miR-545-3p** | 0.715 | 0.02 | 0.551 | 0.878 | | 0.285 | 0.02 | | 0.122 | 0.449 |
| **miR-643** | 0.702 | 0.02 | 0.548 | 0.856 | | 0.298 | 0.02 | | 0.144 | 0.452 |
| **miR-29c-3p** | 0.698 | 0.03 | 0.548 | 0.848 | | 0.302 | 0.03 | | 0.152 | 0.452 |
| **miR-662** | 0.697 | 0.03 | 0.548 | 0.846 | | 0.303 | 0.03 | | 0.154 | 0.452 |
| **miR-548d-5p** | 0.692 | 0.03 | 0.53 | 0.855 | | 0.308 | 0.03 | | 0.145 | 0.47 |
| **miR-320a** | 0.688 | 0.04 | 0.533 | 0.842 | | 0.313 | 0.04 | | 0.158 | 0.467 |
| **miR-106b-3p** | 0.684 | 0.04 | 0.534 | 0.834 | | 0.316 | 0.04 | | 0.166 | 0.466 |
| **miR-886-3p** | 0.681 | 0.04 | 0.52 | 0.841 | | 0.319 | 0.04 | | 0.159 | 0.48 |
| **miR-181c-5p** | 0.677 | 0.05 | 0.521 | 0.833 | | 0.323 | 0.05 | | 0.167 | 0.479 |
| **miR-495-3p** | 0.324 | 0.05 | 0.155 | 0.493 | | 0.676 | 0.05 | | 0.507 | 0.845 |
| **miR-30e-3p** | 0.323 | 0.05 | 0.165 | 0.482 | | 0.677 | 0.05 | | 0.518 | 0.835 |
| **miR-615-3p** | 0.322 | 0.05 | 0.154 | 0.49 | | 0.678 | 0.05 | | 0.51 | 0.846 |
| **miR-19b-1-5p** | 0.32 | 0.04 | 0.168 | 0.473 | | 0.68 | 0.04 | | 0.527 | 0.832 |
| **miR-22-5p** | 0.32 | 0.04 | 0.171 | 0.469 | | 0.68 | 0.04 | | 0.531 | 0.829 |
| **miR-107** | 0.319 | 0.04 | 0.165 | 0.473 | | 0.681 | 0.04 | | 0.527 | 0.835 |
| **miR-342-3p** | 0.319 | 0.04 | 0.164 | 0.475 | | 0.681 | 0.04 | | 0.525 | 0.836 |
| **miR-625-3p** | 0.318 | 0.04 | 0.17 | 0.466 | | 0.682 | 0.04 | | 0.534 | 0.83 |
| **miR-664a-3p** | 0.318 | 0.04 | 0.146 | 0.49 | | 0.682 | 0.04 | | 0.51 | 0.854 |
| **miR-197-3p** | 0.317 | 0.04 | 0.164 | 0.471 | | 0.683 | 0.04 | | 0.529 | 0.836 |
| **miR-23a-3p** | 0.315 | 0.04 | 0.15 | 0.48 | | 0.685 | 0.04 | | 0.52 | 0.85 |
| **miR-194-5p** | 0.314 | 0.04 | 0.149 | 0.48 | | 0.686 | 0.04 | | 0.52 | 0.851 |
| **miR-140** | 0.312 | 0.04 | 0.15 | 0.473 | | 0.688 | 0.04 | | 0.527 | 0.85 |
| **miR-24-2-5p** | 0.31 | 0.03 | 0.142 | 0.477 | | 0.69 | 0.03 | | 0.523 | 0.858 |
| **miR-338-3p** | 0.305 | 0.03 | 0.135 | 0.474 | | 0.695 | 0.03 | | 0.526 | 0.865 |
| **miR-1271-5p** | 0.299 | 0.02 | 0.141 | 0.456 | | 0.701 | 0.02 | | 0.544 | 0.859 |
| **miR-129-5p** | 0.297 | 0.02 | 0.13 | 0.464 | | 0.703 | 0.02 | | 0.536 | 0.87 |
| **miR-301a-3p** | 0.297 | 0.02 | 0.14 | 0.454 | | 0.703 | 0.02 | | 0.546 | 0.86 |
| **miR-10b-3p** | 0.293 | 0.02 | 0.136 | 0.45 | | 0.707 | 0.02 | | 0.55 | 0.864 |
| **miR-10b-5p** | 0.293 | 0.02 | 0.137 | 0.449 | | 0.707 | 0.02 | | 0.551 | 0.863 |
| **miR-485-3p** | 0.293 | 0.02 | 0.135 | 0.451 | | 0.707 | 0.02 | | 0.549 | 0.865 |
| **miR-323a-3p** | 0.292 | 0.02 | 0.133 | 0.451 | | 0.708 | 0.02 | | 0.549 | 0.867 |
| **let-7d-3p** | 0.291 | 0.02 | 0.126 | 0.456 | | 0.709 | 0.02 | | 0.544 | 0.874 |
| **miR-223-5p** | 0.284 | 0.02 | 0.139 | 0.43 | | 0.716 | 0.02 | | 0.57 | 0.861 |
| **miR-425-3p** | 0.278 | 0.01 | 0.126 | 0.431 | | 0.722 | 0.01 | | 0.569 | 0.874 |
| **miR-199a-3p** | 0.275 | 0.01 | 0.13 | 0.42 | | 0.725 | 0.01 | | 0.58 | 0.87 |
| **miR-766-3p** | 0.243 | 0.00 | 0.101 | 0.386 | | 0.757 | 0.00 | | 0.614 | 0.899 |

|  | **SOFTMET vs NOMET** | | | | **NOMET vs SOFTMET** | | | |
| --- | --- | --- | --- | --- | --- | --- | --- | --- |
|  |  | | **Asymptotic 95% Confidence Interval** | |  | | **Asymptotic 95% Confidence Interval** | |
| **miRNA ID** | **Area** | **Asymptotic Signature** | **Lower Bound** | **Upper Bound** | **Area** | **Asymptotic Signature** | **Lower Bound** | **Upper Bound** |
| **miR-579-3p** | 0.75 | 0.02 | 0.577 | 0.923 | 0.25 | 0.02 | 0.077 | 0.423 |
| **miR-643** | 0.748 | 0.02 | 0.57 | 0.926 | 0.252 | 0.02 | 0.074 | 0.43 |
| **miR-451a** | 0.742 | 0.02 | 0.562 | 0.922 | 0.258 | 0.02 | 0.078 | 0.438 |
| **miR-377-5p** | 0.738 | 0.02 | 0.557 | 0.92 | 0.262 | 0.02 | 0.08 | 0.443 |
| **miR-106b-5p** | 0.734 | 0.02 | 0.555 | 0.914 | 0.266 | 0.02 | 0.086 | 0.445 |
| **miR-30a-5p** | 0.734 | 0.02 | 0.55 | 0.918 | 0.266 | 0.02 | 0.082 | 0.45 |
| **miR-361-3p** | 0.734 | 0.02 | 0.551 | 0.917 | 0.266 | 0.02 | 0.083 | 0.449 |
| **miR-106b-3p** | 0.732 | 0.03 | 0.547 | 0.918 | 0.268 | 0.03 | 0.082 | 0.453 |
| **miR-30d-5p** | 0.732 | 0.03 | 0.549 | 0.916 | 0.268 | 0.03 | 0.084 | 0.451 |
| **miR-545-3p** | 0.732 | 0.03 | 0.555 | 0.91 | 0.268 | 0.03 | 0.09 | 0.445 |
| **miR-452-5p** | 0.73 | 0.03 | 0.555 | 0.906 | 0.27 | 0.03 | 0.094 | 0.445 |
| **miR-590-5p** | 0.729 | 0.03 | 0.551 | 0.906 | 0.271 | 0.03 | 0.094 | 0.449 |
| **miR-29c-3p** | 0.723 | 0.03 | 0.543 | 0.903 | 0.277 | 0.03 | 0.097 | 0.457 |
| **miR-708-5p** | 0.719 | 0.04 | 0.539 | 0.899 | 0.281 | 0.04 | 0.101 | 0.461 |
| **miR-320a** | 0.715 | 0.04 | 0.533 | 0.896 | 0.285 | 0.04 | 0.104 | 0.467 |
| **miR-216a-5p** | 0.713 | 0.04 | 0.527 | 0.898 | 0.287 | 0.04 | 0.102 | 0.473 |
| **miR-205-5p** | 0.709 | 0.04 | 0.523 | 0.895 | 0.291 | 0.04 | 0.105 | 0.477 |
| **miR-660-5p** | 0.709 | 0.04 | 0.528 | 0.89 | 0.291 | 0.04 | 0.11 | 0.472 |
| **miR-576-3p** | 0.705 | 0.05 | 0.511 | 0.899 | 0.295 | 0.05 | 0.101 | 0.489 |
| **miR-941** | 0.701 | 0.05 | 0.516 | 0.886 | 0.299 | 0.05 | 0.114 | 0.484 |
| **miR-425-3p** | 0.299 | 0.05 | 0.106 | 0.491 | 0.701 | 0.05 | 0.509 | 0.894 |
| **miR-129-5p** | 0.289 | 0.04 | 0.107 | 0.471 | 0.711 | 0.04 | 0.529 | 0.893 |
| **miR-199a-3p** | 0.289 | 0.04 | 0.107 | 0.471 | 0.711 | 0.04 | 0.529 | 0.893 |
| **miR-766-3p** | 0.275 | 0.03 | 0.096 | 0.455 | 0.725 | 0.03 | 0.545 | 0.904 |
| **miR-107** | 0.244 | 0.01 | 0.075 | 0.414 | 0.756 | 0.01 | 0.586 | 0.925 |

|  | **BONEMET vs NOMET** | | | | **NOMET vs BONEMET** | | | |
| --- | --- | --- | --- | --- | --- | --- | --- | --- |
|  |  | | **Asymptotic 95% Confidence Interval** | |  | | **Asymptotic 95% Confidence Interval** | |
| **miRNA ID** | **Area** | **Asymptotic Signature** | **Lower Bound** | **Upper Bound** | **Area** | **Asymptotic Signature** | **Lower Bound** | **Upper Bound** |
| **miR-1253** | 0.814 | 0.00 | 0.657 | 0.972 | 0.186 | 0.00 | 0.028 | 0.343 |
| **miR-888-5p** | 0.773 | 0.01 | 0.603 | 0.944 | 0.227 | 0.01 | 0.056 | 0.397 |
| **miR-708-5p** | 0.766 | 0.01 | 0.599 | 0.932 | 0.234 | 0.01 | 0.068 | 0.401 |
| **miR-662** | 0.754 | 0.01 | 0.585 | 0.923 | 0.246 | 0.01 | 0.077 | 0.415 |
| **miR-548d-5p** | 0.746 | 0.02 | 0.576 | 0.916 | 0.254 | 0.02 | 0.084 | 0.424 |
| **miR-30a-5p** | 0.729 | 0.03 | 0.541 | 0.916 | 0.271 | 0.03 | 0.084 | 0.459 |
| **miR-577** | 0.719 | 0.04 | 0.54 | 0.898 | 0.281 | 0.04 | 0.102 | 0.46 |
| **miR-30d-5p** | 0.711 | 0.04 | 0.523 | 0.898 | 0.289 | 0.04 | 0.102 | 0.477 |
| **miR-296-3p** | 0.703 | 0.05 | 0.513 | 0.893 | 0.297 | 0.05 | 0.107 | 0.487 |
| **miR-886-3p** | 0.701 | 0.05 | 0.514 | 0.889 | 0.299 | 0.05 | 0.111 | 0.486 |
| **miR-146b** | 0.297 | 0.05 | 0.109 | 0.484 | 0.703 | 0.05 | 0.516 | 0.891 |
| **miR-142-3p** | 0.295 | 0.05 | 0.109 | 0.481 | 0.705 | 0.05 | 0.519 | 0.891 |
| **miR-26a-1-3p** | 0.293 | 0.05 | 0.107 | 0.479 | 0.707 | 0.05 | 0.521 | 0.893 |
| **miR-19b-1-5p** | 0.291 | 0.04 | 0.092 | 0.49 | 0.709 | 0.04 | 0.51 | 0.908 |
| **miR-29c-5p** | 0.291 | 0.04 | 0.109 | 0.473 | 0.709 | 0.04 | 0.527 | 0.891 |
| **miR-664a-3p** | 0.291 | 0.04 | 0.098 | 0.484 | 0.709 | 0.04 | 0.516 | 0.902 |
| **miR-93-3p** | 0.291 | 0.04 | 0.104 | 0.478 | 0.709 | 0.04 | 0.522 | 0.896 |
| **miR-18b-5p** | 0.289 | 0.04 | 0.099 | 0.479 | 0.711 | 0.04 | 0.521 | 0.901 |
| **miR-145-3p** | 0.285 | 0.04 | 0.104 | 0.466 | 0.715 | 0.04 | 0.534 | 0.896 |
| **miR-10b-5p** | 0.281 | 0.04 | 0.1 | 0.463 | 0.719 | 0.04 | 0.537 | 0.9 |
| **miR-194-5p** | 0.281 | 0.04 | 0.102 | 0.461 | 0.719 | 0.04 | 0.539 | 0.898 |
| **miR-197-3p** | 0.281 | 0.04 | 0.1 | 0.462 | 0.719 | 0.04 | 0.538 | 0.9 |
| **miR-20b-5p** | 0.281 | 0.04 | 0.093 | 0.469 | 0.719 | 0.04 | 0.531 | 0.907 |
| **miR-409-3p** | 0.281 | 0.04 | 0.097 | 0.465 | 0.719 | 0.04 | 0.535 | 0.903 |
| **miR-543** | 0.281 | 0.04 | 0.092 | 0.47 | 0.719 | 0.04 | 0.53 | 0.908 |
| **miR-21-5p** | 0.277 | 0.03 | 0.095 | 0.459 | 0.723 | 0.03 | 0.541 | 0.905 |
| **miR-24-3p** | 0.275 | 0.03 | 0.097 | 0.454 | 0.725 | 0.03 | 0.546 | 0.903 |
| **miR-10b-3p** | 0.271 | 0.03 | 0.087 | 0.456 | 0.729 | 0.03 | 0.544 | 0.913 |
| **miR-1271-5p** | 0.271 | 0.03 | 0.088 | 0.455 | 0.729 | 0.03 | 0.545 | 0.912 |
| **miR-148a-5p** | 0.271 | 0.03 | 0.094 | 0.449 | 0.729 | 0.03 | 0.551 | 0.906 |
| **miR-30e-3p** | 0.27 | 0.03 | 0.089 | 0.45 | 0.73 | 0.03 | 0.55 | 0.911 |
| **miR-223-5p** | 0.264 | 0.02 | 0.086 | 0.441 | 0.736 | 0.02 | 0.559 | 0.914 |
| **miR-199a-3p** | 0.262 | 0.02 | 0.09 | 0.433 | 0.738 | 0.02 | 0.567 | 0.91 |
| **miR-769-5p** | 0.26 | 0.02 | 0.078 | 0.442 | 0.74 | 0.02 | 0.558 | 0.922 |
| **miR-145** | 0.258 | 0.02 | 0.074 | 0.442 | 0.742 | 0.02 | 0.558 | 0.926 |
| **miR-425-3p** | 0.258 | 0.02 | 0.078 | 0.437 | 0.742 | 0.02 | 0.563 | 0.922 |
| **miR-758-3p** | 0.254 | 0.02 | 0.079 | 0.429 | 0.746 | 0.02 | 0.571 | 0.921 |
| **miR-24-2-5p** | 0.252 | 0.02 | 0.081 | 0.423 | 0.748 | 0.02 | 0.577 | 0.919 |
| **miR-338-3p** | 0.252 | 0.02 | 0.073 | 0.431 | 0.748 | 0.02 | 0.569 | 0.927 |
| **miR-128-3p** | 0.246 | 0.01 | 0.063 | 0.43 | 0.754 | 0.01 | 0.57 | 0.937 |
| **miR-532-5p** | 0.246 | 0.01 | 0.072 | 0.42 | 0.754 | 0.01 | 0.58 | 0.928 |
| **miR-23a-3p** | 0.244 | 0.01 | 0.065 | 0.424 | 0.756 | 0.01 | 0.576 | 0.935 |
| **miR-323a-3p** | 0.24 | 0.01 | 0.061 | 0.419 | 0.76 | 0.01 | 0.581 | 0.939 |
| **let-7d-3p** | 0.238 | 0.01 | 0.065 | 0.412 | 0.762 | 0.01 | 0.588 | 0.935 |
| **miR-1201** | 0.238 | 0.01 | 0.068 | 0.408 | 0.762 | 0.01 | 0.592 | 0.932 |
| **miR-495-3p** | 0.234 | 0.01 | 0.07 | 0.398 | 0.766 | 0.01 | 0.602 | 0.93 |
| **miR-301a-3p** | 0.23 | 0.01 | 0.066 | 0.394 | 0.77 | 0.01 | 0.606 | 0.934 |
| **miR-485-3p** | 0.215 | 0.01 | 0.056 | 0.374 | 0.785 | 0.01 | 0.626 | 0.944 |
| **miR-766-3p** | 0.211 | 0.01 | 0.045 | 0.377 | 0.789 | 0.01 | 0.623 | 0.955 |
| **miR-140** | 0.209 | 0.01 | 0.05 | 0.368 | 0.791 | 0.01 | 0.632 | 0.95 |
| **miR-22-5p** | 0.195 | 0.00 | 0.028 | 0.363 | 0.805 | 0.00 | 0.637 | 0.972 |
