# Supplementary material for: MiR-662 is associated with metastatic relapse in early-stage breast cancer and promotes metastasis by stimulating cancer cell stemness
Source: Br J Cancer. 2023 Jul 13;129(5):754–71. doi: 10.1038/s41416-023-02340-9 (PMC10449914; doi:10.1038/s41416-023-02340-9)
Supplement: Supplementary file 7 — Supplementary Table 6 [file 41416_2023_2340_MOESM7_ESM.pdf]

Table S6

GOID	GOTerm
GO:0001102	RNA polymerase II activating transcription factor binding
GO:0090307	mitotic spindle assembly
GO:2000378	negative regulation of reactive oxygen species metabolic process
KEGG:04672	Intestinal immune network for IgA production
GO:0002066	columnar/cuboidal epithelial cell development
GO:0045576	mast cell activation
GO:0046324	regulation of glucose import
GO:0046635	positive regulation of alpha-beta T cell activation
GO:0055081	anion homeostasis
GO:0071230	cellular response to amino acid stimulus
GO:0070849	response to epidermal growth factor
GO:0071364	cellular response to epidermal growth factor stimulus
GO:0032233	positive regulation of actin filament bundle assembly
GO:0070262	peptidyl-serine dephosphorylation
GO:0008138	protein tyrosine/serine/threonine phosphatase activity
GO:0043280	positive regulation of cysteine-type endopeptidase activity involved in apoptotic process
KEGG:04071	Sphingolipid signaling pathway
GO:0070997	neuron death
GO:1901214	regulation of neuron death
GO:1901215	negative regulation of neuron death
GO:0043524	negative regulation of neuron apoptotic process
GO:0008344	adult locomotory behavior
GO:0072678	T cell migration
GO:2000401	regulation of lymphocyte migration
GO:2000403	positive regulation of lymphocyte migration
GO:2000404	regulation of T cell migration
GO:2000406	positive regulation of T cell migration
R-HSA:193648	NRAGE signals death through JNK
R-HSA:193704	p75 NTR receptor-mediated signalling
R-HSA:194913	GEFs activate Rho GTPase:GDP
R-HSA:205039	p75NTR indirectly activates RAC and Cdc42 via a guanyl-nucleotide exchange factor
R-HSA:416482	G alpha (12/13) signalling events
R-HSA:419166	GEFs activate RhoA,B,C
KEGG:04071	Sphingolipid signaling pathway
GO:0001836	release of cytochrome c from mitochondria
GO:0043525	positive regulation of neuron apoptotic process
GO:2001244	positive regulation of intrinsic apoptotic signaling pathway
GO:0032233	positive regulation of actin filament bundle assembly
GO:0032469	endoplasmic reticulum calcium ion homeostasis
GO:0043280	positive regulation of cysteine-type endopeptidase activity involved in apoptotic process
GO:0019213	deacetylase activity
GO:0016811	hydrolase activity, acting on carbon-nitrogen (but not peptide) bonds, in linear amides
GO:0098732	macromolecule deacylation
GO:0035601	protein deacylation
GO:0016575	histone deacetylation
GO:0033558	protein deacetylase activity
GO:0006342	chromatin silencing
GO:0004407	histone deacetylase activity
GO:0006348	chromatin silencing at telomere
KEGG:04071	Sphingolipid signaling pathway
GO:0051281	positive regulation of release of sequestered calcium ion into cytosol
GO:1900117	regulation of execution phase of apoptosis
GO:0001938	positive regulation of endothelial cell proliferation
GO:0043524	negative regulation of neuron apoptotic process
GO:0043525	positive regulation of neuron apoptotic process
GO:0018107	peptidyl-threonine phosphorylation
GO:1904427	positive regulation of calcium ion transmembrane transport

GO:0010524	**positive regulation of calcium ion transport into cytosol**
GO:0010799	**regulation of peptidyl-threonine phosphorylation**
GO:0090279	**regulation of calcium ion import**
R-HSA:2299718	**Condensation of Prophase Chromosomes**
R-HSA:3214841	**PKMTs methylate histone lysines**
R-HSA:3214842	**HDMs demethylate histones**
R-HSA:4722133	**KDM2A, KDM2B, KDM4A demethylate MeK37-histone H3**
R-HSA:5625740	**RHO GTPases activate PKNs**
R-HSA:5649764	**DOT1L (KMT4) methylates methyl-lysine-80 of histone H3 (H3K79)**
R-HSA:5649799	**DOT1L (KMT4) methylates dimethyl-lysine-80 of histone H3 (H3K79)**
R-HSA:5649801	**DOT1L (KMT4) methylates lysine-80 of histone H3 (H3K79)**
R-HSA:5661114	**KDM2A, KDM2B, KDM4A demethylate Me2K37-histone H3**
R-HSA:9604829	**SIRT6 deacetylates histones at NOTCH1 and NOTCH4 gene promoters**
R-HSA:9604831	**NOTCH1 and NOTCH4 gene transcription is inhibited by SIRT6**
R-HSA:9604834	**SIRT6 binds to acetylated histones at NOTCH1 and NOTCH4 gene promoters**
R-HSA:977225	**Amyloid fiber formation**
GO:0035094	**response to nicotine**
GO:0042982	**amyloid precursor protein metabolic process**
GO:1990000	**amyloid fibril formation**
GO:0042987	**amyloid precursor protein catabolic process**
GO:0050435	**amyloid-beta metabolic process**
GO:0099601	**regulation of neurotransmitter receptor activity**
GO:1904645	**response to amyloid-beta**
GO:1905906	**regulation of amyloid fibril formation**
GO:0098661	**inorganic anion transmembrane transport**
GO:1902991	**regulation of amyloid precursor protein catabolic process**
GO:1903556	**negative regulation of tumor necrosis factor superfamily cytokine production**
GO:0034205	**amyloid-beta formation**
GO:1902003	**regulation of amyloid-beta formation**
GO:1902993	**positive regulation of amyloid precursor protein catabolic process**
GO:0006821	**chloride transport**
GO:0032720	**negative regulation of tumor necrosis factor production**
GO:0060079	**excitatory postsynaptic potential**
GO:0060291	**long-term synaptic potentiation**
GO:0098815	**modulation of excitatory postsynaptic potential**
GO:0098960	**postsynaptic neurotransmitter receptor activity**
GO:1902004	**positive regulation of amyloid-beta formation**
GO:1902476	**chloride transmembrane transport**
GO:0005253	**anion channel activity**
GO:0007271	**synaptic transmission, cholinergic**
GO:0015108	**chloride transmembrane transporter activity**
GO:0099529	**neurotransmitter receptor activity involved in regulation of postsynaptic membrane potential**
GO:1900271	**regulation of long-term synaptic potentiation**
GO:0015464	**acetylcholine receptor activity**
GO:1900273	**positive regulation of long-term synaptic potentiation**
GO:0005254	**chloride channel activity**
GO:0017081	**chloride channel regulator activity**
GO:0005231	**excitatory extracellular ligand-gated ion channel activity**
GO:1904315	**transmitter-gated ion channel activity involved in regulation of postsynaptic membrane potential**

Ontology Source	Term PValue	Term PValue C	Group PValue
GO_MolecularFunction-EBI-UniProt-GOA-ACAP-ARAP_08.05.2020_00h00	0.01	0.15	0.01
GO_BiologicalProcess-EBI-UniProt-GOA-ACAP-ARAP_08.05.2020_00h00	0.02	0.13	0.02
GO_BiologicalProcess-EBI-UniProt-GOA-ACAP-ARAP_08.05.2020_00h00	0.02	0.12	0.02
KEGG_08.05.2020	0.01	0.15	0.01
GO_BiologicalProcess-EBI-UniProt-GOA-ACAP-ARAP_08.05.2020_00h00	0.02	0.14	0.02
GO_BiologicalProcess-EBI-UniProt-GOA-ACAP-ARAP_08.05.2020_00h00	0.00	0.09	0.00
GO_BiologicalProcess-EBI-UniProt-GOA-ACAP-ARAP_08.05.2020_00h00	0.02	0.14	0.02
GO_BiologicalProcess-EBI-UniProt-GOA-ACAP-ARAP_08.05.2020_00h00	0.02	0.12	0.02
GO_BiologicalProcess-EBI-UniProt-GOA-ACAP-ARAP_08.05.2020_00h00	0.02	0.07	0.02
GO_BiologicalProcess-EBI-UniProt-GOA-ACAP-ARAP_08.05.2020_00h00	0.02	0.07	0.02
GO_BiologicalProcess-EBI-UniProt-GOA-ACAP-ARAP_08.05.2020_00h00	0.01	0.14	0.01
GO_BiologicalProcess-EBI-UniProt-GOA-ACAP-ARAP_08.05.2020_00h00	0.01	0.15	0.01
GO_BiologicalProcess-EBI-UniProt-GOA-ACAP-ARAP_08.05.2020_00h00	0.02	0.11	0.00
GO_BiologicalProcess-EBI-UniProt-GOA-ACAP-ARAP_08.05.2020_00h00	0.00	0.04	0.00
GO_BiologicalProcess-EBI-UniProt-GOA-ACAP-ARAP_08.05.2020_00h00	0.01	0.15	0.00
GO_BiologicalProcess-EBI-UniProt-GOA-ACAP-ARAP_08.05.2020_00h00	0.00	0.06	0.00
KEGG_08.05.2020	0.00	0.11	0.00
GO_BiologicalProcess-EBI-UniProt-GOA-ACAP-ARAP_08.05.2020_00h00	0.00	0.00	0.00
GO_BiologicalProcess-EBI-UniProt-GOA-ACAP-ARAP_08.05.2020_00h00	0.00	0.00	0.00
GO_BiologicalProcess-EBI-UniProt-GOA-ACAP-ARAP_08.05.2020_00h00	0.00	0.00	0.00
GO_BiologicalProcess-EBI-UniProt-GOA-ACAP-ARAP_08.05.2020_00h00	0.00	0.02	0.00
GO_BiologicalProcess-EBI-UniProt-GOA-ACAP-ARAP_08.05.2020_00h00	0.01	0.15	0.01
GO_BiologicalProcess-EBI-UniProt-GOA-ACAP-ARAP_08.05.2020_00h00	0.02	0.10	0.01
GO_BiologicalProcess-EBI-UniProt-GOA-ACAP-ARAP_08.05.2020_00h00	0.02	0.13	0.01
GO_BiologicalProcess-EBI-UniProt-GOA-ACAP-ARAP_08.05.2020_00h00	0.00	0.13	0.01
GO_BiologicalProcess-EBI-UniProt-GOA-ACAP-ARAP_08.05.2020_00h00	0.01	0.15	0.01
GO_BiologicalProcess-EBI-UniProt-GOA-ACAP-ARAP_08.05.2020_00h00	0.00	0.09	0.01
REACTOME_Pathways_08.05.2020	0.01	0.15	0.03
REACTOME_Pathways_08.05.2020	0.01	0.14	0.03
REACTOME_Reactions_08.05.2020	0.00	0.10	0.03
REACTOME_Reactions_08.05.2020	0.01	0.14	0.03
REACTOME_Pathways_08.05.2020	0.00	0.13	0.03
REACTOME_Reactions_08.05.2020	0.00	0.05	0.03
KEGG_08.05.2020	0.00	0.11	0.00
GO_BiologicalProcess-EBI-UniProt-GOA-ACAP-ARAP_08.05.2020_00h00	0.02	0.11	0.00
GO_BiologicalProcess-EBI-UniProt-GOA-ACAP-ARAP_08.05.2020_00h00	0.01	0.15	0.00
GO_BiologicalProcess-EBI-UniProt-GOA-ACAP-ARAP_08.05.2020_00h00	0.01	0.15	0.00
GO_BiologicalProcess-EBI-UniProt-GOA-ACAP-ARAP_08.05.2020_00h00	0.02	0.11	0.00
GO_BiologicalProcess-EBI-UniProt-GOA-ACAP-ARAP_08.05.2020_00h00	0.00	0.05	0.00
GO_BiologicalProcess-EBI-UniProt-GOA-ACAP-ARAP_08.05.2020_00h00	0.00	0.06	0.00
GO_MolecularFunction-EBI-UniProt-GOA-ACAP-ARAP_08.05.2020_00h00	0.00	0.06	0.02
GO_MolecularFunction-EBI-UniProt-GOA-ACAP-ARAP_08.05.2020_00h00	0.01	0.15	0.02
GO_BiologicalProcess-EBI-UniProt-GOA-ACAP-ARAP_08.05.2020_00h00	0.00	0.09	0.02
GO_BiologicalProcess-EBI-UniProt-GOA-ACAP-ARAP_08.05.2020_00h00	0.00	0.09	0.02
GO_BiologicalProcess-EBI-UniProt-GOA-ACAP-ARAP_08.05.2020_00h00	0.00	0.14	0.02
GO_BiologicalProcess-EBI-UniProt-GOA-ACAP-ARAP_08.05.2020_00h00	0.00	0.03	0.02
GO_BiologicalProcess-EBI-UniProt-GOA-ACAP-ARAP_08.05.2020_00h00	0.02	0.05	0.02
GO_BiologicalProcess-EBI-UniProt-GOA-ACAP-ARAP_08.05.2020_00h00	0.00	0.02	0.02
GO_BiologicalProcess-EBI-UniProt-GOA-ACAP-ARAP_08.05.2020_00h00	0.00	0.00	0.02
KEGG_08.05.2020	0.00	0.11	0.00
GO_BiologicalProcess-EBI-UniProt-GOA-ACAP-ARAP_08.05.2020_00h00	0.01	0.15	0.00
GO_BiologicalProcess-EBI-UniProt-GOA-ACAP-ARAP_08.05.2020_00h00	0.01	0.16	0.00
GO_BiologicalProcess-EBI-UniProt-GOA-ACAP-ARAP_08.05.2020_00h00	0.00	0.08	0.00
GO_BiologicalProcess-EBI-UniProt-GOA-ACAP-ARAP_08.05.2020_00h00	0.00	0.02	0.00
GO_BiologicalProcess-EBI-UniProt-GOA-ACAP-ARAP_08.05.2020_00h00	0.01	0.15	0.00
GO_BiologicalProcess-EBI-UniProt-GOA-ACAP-ARAP_08.05.2020_00h00	0.00	0.12	0.00
GO_BiologicalProcess-EBI-UniProt-GOA-ACAP-ARAP_08.05.2020_00h00	0.02	0.02	0.00

GO_BiologicalProcess-EBI-UniProt-GOA-ACAP-ARAP_08.05.2020_00h00	0.01	0.15	0.00
GO_BiologicalProcess-EBI-UniProt-GOA-ACAP-ARAP_08.05.2020_00h00	0.01	0.16	0.00
GO_BiologicalProcess-EBI-UniProt-GOA-ACAP-ARAP_08.05.2020_00h00	0.01	0.14	0.00
REACTOME_Pathways_08.05.2020	0.02	0.05	0.00
REACTOME_Pathways_08.05.2020	0.02	0.08	0.00
REACTOME_Pathways_08.05.2020	0.01	0.15	0.00
REACTOME_Reactions_08.05.2020	0.00	0.02	0.00
REACTOME_Pathways_08.05.2020	0.01	0.15	0.00
REACTOME_Reactions_08.05.2020	0.00	0.01	0.00
REACTOME_Reactions_08.05.2020	0.00	0.01	0.00
REACTOME_Reactions_08.05.2020	0.00	0.01	0.00
REACTOME_Reactions_08.05.2020	0.00	0.02	0.00
REACTOME_Reactions_08.05.2020	0.01	0.14	0.00
REACTOME_Reactions_08.05.2020	0.01	0.14	0.00
REACTOME_Reactions_08.05.2020	0.01	0.14	0.00
REACTOME_Pathways_08.05.2020	0.00	0.09	0.00
GO_BiologicalProcess-EBI-UniProt-GOA-ACAP-ARAP_08.05.2020_00h00	0.00	0.06	0.00
GO_BiologicalProcess-EBI-UniProt-GOA-ACAP-ARAP_08.05.2020_00h00	0.02	0.08	0.00
GO_BiologicalProcess-EBI-UniProt-GOA-ACAP-ARAP_08.05.2020_00h00	0.00	0.00	0.00
GO_BiologicalProcess-EBI-UniProt-GOA-ACAP-ARAP_08.05.2020_00h00	0.01	0.14	0.00
GO_BiologicalProcess-EBI-UniProt-GOA-ACAP-ARAP_08.05.2020_00h00	0.01	0.15	0.00
GO_BiologicalProcess-EBI-UniProt-GOA-ACAP-ARAP_08.05.2020_00h00	0.01	0.15	0.00
GO_BiologicalProcess-EBI-UniProt-GOA-ACAP-ARAP_08.05.2020_00h00	0.00	0.04	0.00
GO_BiologicalProcess-EBI-UniProt-GOA-ACAP-ARAP_08.05.2020_00h00	0.00	0.00	0.00
GO_BiologicalProcess-EBI-UniProt-GOA-ACAP-ARAP_08.05.2020_00h00	0.00	0.05	0.00
GO_BiologicalProcess-EBI-UniProt-GOA-ACAP-ARAP_08.05.2020_00h00	0.01	0.15	0.00
GO_BiologicalProcess-EBI-UniProt-GOA-ACAP-ARAP_08.05.2020_00h00	0.02	0.12	0.00
GO_BiologicalProcess-EBI-UniProt-GOA-ACAP-ARAP_08.05.2020_00h00	0.01	0.15	0.00
GO_BiologicalProcess-EBI-UniProt-GOA-ACAP-ARAP_08.05.2020_00h00	0.00	0.12	0.00
GO_BiologicalProcess-EBI-UniProt-GOA-ACAP-ARAP_08.05.2020_00h00	0.00	0.06	0.00
GO_BiologicalProcess-EBI-UniProt-GOA-ACAP-ARAP_08.05.2020_00h00	0.00	0.04	0.00
GO_BiologicalProcess-EBI-UniProt-GOA-ACAP-ARAP_08.05.2020_00h00	0.02	0.13	0.00
GO_BiologicalProcess-EBI-UniProt-GOA-ACAP-ARAP_08.05.2020_00h00	0.00	0.09	0.00
GO_BiologicalProcess-EBI-UniProt-GOA-ACAP-ARAP_08.05.2020_00h00	0.01	0.14	0.00
GO_BiologicalProcess-EBI-UniProt-GOA-ACAP-ARAP_08.05.2020_00h00	0.01	0.15	0.00
GO_BiologicalProcess-EBI-UniProt-GOA-ACAP-ARAP_08.05.2020_00h00	0.00	0.09	0.00
GO_BiologicalProcess-EBI-UniProt-GOA-ACAP-ARAP_08.05.2020_00h00	0.00	0.04	0.00
GO_BiologicalProcess-EBI-UniProt-GOA-ACAP-ARAP_08.05.2020_00h00	0.00	0.03	0.00
GO_BiologicalProcess-EBI-UniProt-GOA-ACAP-ARAP_08.05.2020_00h00	0.00	0.10	0.00
GO_BiologicalProcess-EBI-UniProt-GOA-ACAP-ARAP_08.05.2020_00h00	0.00	0.05	0.00
GO_BiologicalProcess-EBI-UniProt-GOA-ACAP-ARAP_08.05.2020_00h00	0.00	0.03	0.00
GO_BiologicalProcess-EBI-UniProt-GOA-ACAP-ARAP_08.05.2020_00h00	0.01	0.15	0.00
GO_BiologicalProcess-EBI-UniProt-GOA-ACAP-ARAP_08.05.2020_00h00	0.00	0.04	0.00
GO_BiologicalProcess-EBI-UniProt-GOA-ACAP-ARAP_08.05.2020_00h00	0.00	0.02	0.00
GO_BiologicalProcess-EBI-UniProt-GOA-ACAP-ARAP_08.05.2020_00h00	0.00	0.05	0.00
GO_BiologicalProcess-EBI-UniProt-GOA-ACAP-ARAP_08.05.2020_00h00	0.00	0.05	0.00
GO_BiologicalProcess-EBI-UniProt-GOA-ACAP-ARAP_08.05.2020_00h00	0.00	0.02	0.00
GO_BiologicalProcess-EBI-UniProt-GOA-ACAP-ARAP_08.05.2020_00h00	0.00	0.10	0.00
GO_BiologicalProcess-EBI-UniProt-GOA-ACAP-ARAP_08.05.2020_00h00	0.01	0.15	0.00

Group PValue Co	GOLevels	GOGroups
0.10	[6]	Group00
0.06	[5, 6, 7, 8, 9]	Group01
0.05	[4, 5, 6]	Group02
0.08	[-1]	Group03
0.08	[5, 6, 7]	Group04
0.03	[4, 5]	Group05
0.08	[5, 6, 7, 8, 10, 11]	Group06
0.05	[6, 7, 8, 9]	Group07
0.04	[6]	Group08
0.04	[5, 6]	Group09
0.09	[3, 5]	Group10
0.09	[4, 6]	Group10
0.00	[4, 5, 6, 7, 8, 9, 10, 11]	Group11
0.00	[7, 8]	Group11
0.00	[8, 9]	Group11
0.00	[6, 7, 8, 9, 10, 11, 12]	Group11
0.00	[-1]	Group12
0.00	[3]	Group12
0.00	[4, 5]	Group12
0.00	[4, 5, 6]	Group12
0.00	[5, 6, 7, 8]	Group12
0.10	[3]	Group13
0.10	[4, 6, 7]	Group13
0.10	[4, 5, 6, 7, 8]	Group13
0.10	[4, 5, 6, 7, 8, 9]	Group13
0.10	[5, 6, 7, 8, 9]	Group13
0.10	[5, 6, 7, 8, 9, 10]	Group13
0.03	[-1]	Group14
0.03	[-1]	Group14
0.03	[-1]	Group14
0.03	[-1]	Group14
0.03	[-1]	Group14
0.03	[-1]	Group14
0.00	[-1]	Group15
0.00	[4, 5, 6, 7]	Group15
0.00	[5, 6, 7, 8]	Group15
0.00	[5, 6, 7, 8, 9]	Group15
0.00	[4, 5, 6, 7, 8, 9, 10, 11]	Group15
0.00	[8, 10]	Group15
0.00	[6, 7, 8, 9, 10, 11, 12]	Group15
0.09	[3]	Group16
0.09	[4]	Group16
0.09	[5]	Group16
0.09	[6, 7]	Group16
0.09	[7, 8, 9]	Group16
0.09	[8, 9]	Group16
0.09	[3, 6, 7, 8, 9, 10, 11, 12, 13]	Group16
0.09	[8, 9, 10]	Group16
0.09	[4, 7, 8, 9, 10, 11, 12, 13, 14]	Group16
0.00	[-1]	Group17
0.00	[4, 5, 6, 7, 8, 9, 10, 11, 12, 13, 14, 15, 16]	Group17
0.00	[3, 6, 7]	Group17
0.00	[5, 6, 7]	Group17
0.00	[5, 6, 7, 8]	Group17
0.00	[5, 6, 7, 8]	Group17
0.00	[7, 8, 9]	Group17
0.00	[5, 6, 7, 8, 9, 10, 11]	Group17

0.00	[3, 4, 5, 6, 7, 8, 9, 10, 11, 12, 13, 14]	Group17
0.00	[7, 8, 9, 10]	Group17
0.00	[7, 8, 9, 10]	Group17
0.00	[-1]	Group18
0.00	[-1]	Group18
0.00	[-1]	Group18
0.00	[-1]	Group18
0.00	[-1]	Group18
0.00	[-1]	Group18
0.00	[-1]	Group18
0.00	[-1]	Group18
0.00	[-1]	Group18
0.00	[-1]	Group18
0.00	[-1]	Group18
0.00	[-1]	Group18
0.00	[-1]	Group18
0.00	[4]	Group19
0.00	[4, 5]	Group19
0.00	[4, 5]	Group19
0.00	[5, 6]	Group19
0.00	[5, 6]	Group19
0.00	[4, 5, 6, 7]	Group19
0.00	[5, 6]	Group19
0.00	[5, 6]	Group19
0.00	[5, 7]	Group19
0.00	[5, 6, 7]	Group19
0.00	[4, 5, 6, 7, 8]	Group19
0.00	[6, 7]	Group19
0.00	[5, 6, 7, 8]	Group19
0.00	[5, 6, 7, 8]	Group19
0.00	[7]	Group19
0.00	[5, 6, 7, 8, 9]	Group19
0.00	[5, 6, 7, 8, 9]	Group19
0.00	[4, 5, 6, 7, 8, 9, 10]	Group19
0.00	[4, 5, 6, 7, 8, 9, 10]	Group19
0.00	[5, 6, 7, 8, 9]	Group19
0.00	[5, 6, 7, 8, 9]	Group19
0.00	[6, 8]	Group19
0.00	[6, 7, 8, 9]	Group19
0.00	[7, 8]	Group19
0.00	[6, 7, 8, 9]	Group19
0.00	[5, 6, 7, 8, 9, 10]	Group19
0.00	[4, 5, 6, 7, 8, 9, 10, 11]	Group19
0.00	[6, 7, 8, 9, 10]	Group19
0.00	[4, 5, 6, 7, 8, 9, 10, 11, 12]	Group19
0.00	[7, 8, 9, 10]	Group19
0.00	[5, 8, 9, 10, 11]	Group19
0.00	[6, 7, 8, 9, 10, 11, 12]	Group19
0.00	[6, 7, 8, 9, 10, 11, 12, 13]	Group19

% Associated Genes	Nr. Genes
5.08	3.00
4.69	3.00
4.55	3.00
6.12	3.00
4.76	3.00
5.97	4.00
4.76	3.00
4.55	3.00
4.17	3.00
4.17	3.00
5.56	3.00
6.00	3.00
4.48	3.00
13.64	3.00
6.25	3.00
4.23	6.00
4.20	5.00
4.16	16.00
4.11	14.00
4.39	10.00
4.38	7.00
4.49	4.00
4.29	3.00
4.69	3.00
7.69	3.00
6.67	3.00
9.38	3.00
5.08	3.00
4.12	4.00
5.71	4.00
5.77	3.00
5.00	4.00
7.41	4.00
4.20	5.00
4.48	3.00
5.00	3.00
5.08	3.00
4.48	3.00
12.00	3.00
4.23	6.00
6.78	4.00
4.21	4.00
4.42	5.00
4.50	5.00
4.82	4.00
9.30	4.00
4.05	3.00
9.52	4.00
30.00	3.00
4.20	5.00
6.98	3.00
6.38	3.00
4.72	5.00
4.38	7.00
5.00	3.00
4.07	5.00
4.00	3.00

5.08	3.00
6.38	3.00
7.14	3.00
4.05	3.00
4.23	3.00
6.00	3.00
18.75	3.00
4.21	4.00
21.43	3.00
21.43	3.00
21.43	3.00
18.75	3.00
4.92	3.00
4.92	3.00
4.92	3.00
4.59	5.00
6.90	4.00
4.23	3.00
21.05	4.00
5.56	3.00
5.08	3.00
4.40	4.00
8.00	4.00
44.44	4.00
4.29	6.00
6.82	3.00
4.55	3.00
6.82	3.00
7.89	3.00
11.54	3.00
4.55	6.00
4.69	3.00
4.59	5.00
4.17	4.00
6.25	3.00
4.59	5.00
13.64	3.00
4.92	6.00
4.39	5.00
7.69	4.00
5.00	6.00
5.00	3.00
8.00	4.00
10.26	4.00
12.00	3.00
5.38	5.00
17.65	3.00
8.82	3.00
5.36	3.00

Associated Genes Found
[BEX1, DUSP28, TBX15]
[CHMP1A, NDRG1, RHOA]
[HK2, RHOA, SIRT2]
[CCR10, CXCL12, LTBR]
[NKX6-1, PDPK1, YIPF6]
[CD300LF, CPLX2, NDRG1, PDPK1]
[HK2, OSTN, SIRT6]
[EBI3, RHOA, TNFSF4]
[ADORA1, BSG, SLC12A7]
[BCL2L2, GLRA1, SH3BP4]
[DNAJC5, EEF1A1, PDPK1]
[DNAJC5, EEF1A1, PDPK1]
[PPM1F, RHOA, SWAP70]
[DUSP28, PPM1F, SWAP70]
[DUSP28, DUSP5, PPM1F]
[BAX, FIS1, IAPP, PDCD6, PPM1F, RHOA]
[ADORA1, BAX, PDPK1, RHOA, S1PR3]
[ADORA1, APP, BAX, BSG, CHRFAM7A, CHRNA7, CSF3, DNAJC5, FIS1, ITSN1, KDM2B, PDPK1, RHOA, SET, SIAH1, T
[ADORA1, BAX, BSG, CHRFAM7A, CHRNA7, CSF3, DNAJC5, FIS1, ITSN1, KDM2B, PDPK1, RHOA, SET, TMEM259]
[BAX, BSG, CSF3, DNAJC5, ITSN1, KDM2B, PDPK1, RHOA, SET, TMEM259]
[BAX, DNAJC5, ITSN1, KDM2B, PDPK1, RHOA, SET]
[ABHD12, APP, CXCL12, GLRA1]
[APP, CXCL12, RHOA]
[APP, CXCL12, RHOA]
[APP, CXCL12, RHOA]
[APP, CXCL12, RHOA]
[APP, CXCL12, RHOA]
[ARHGEF37, FGD1, ITSN1]
[ARHGEF37, FGD1, ITSN1, RHOA]
[ARHGEF37, FGD1, ITSN1, RHOA]
[ARHGEF37, FGD1, ITSN1]
[ARHGEF37, FGD1, ITSN1, RHOA]
[ARHGEF37, FGD1, ITSN1, RHOA]
[ADORA1, BAX, PDPK1, RHOA, S1PR3]
[BAX, BCL2L2, FIS1]
[BAX, FIS1, RHOA]
[BAX, FIS1, SIAH1]
[PPM1F, RHOA, SWAP70]
[APP, BAX, FIS1]
[BAX, FIS1, IAPP, PDCD6, PPM1F, RHOA]
[MIER2, PRKD2, SIRT2, SIRT6]
[MIER2, PRKD2, SIRT2, SIRT6]
[ABHD12, MIER2, PRKD2, SIRT2, SIRT6]
[ABHD12, MIER2, PRKD2, SIRT2, SIRT6]
[MIER2, PRKD2, SIRT2, SIRT6]
[MIER2, PRKD2, SIRT2, SIRT6]
[DOT1L, SIRT2, SIRT6]
[MIER2, PRKD2, SIRT2, SIRT6]
[DOT1L, SIRT2, SIRT6]
[ADORA1, BAX, PDPK1, RHOA, S1PR3]
[BAX, PDPK1, PRKD2]
[BAX, GCG, SIRT2]
[CXCL12, PDCD6, PDPK1, PRKD2, SIRT6]
[BAX, DNAJC5, ITSN1, KDM2B, PDPK1, RHOA, SET]
[BAX, FIS1, RHOA]
[APP, GCG, PDPK1, PRKD2, SIRT2]
[BAX, PDPK1, PRKD2]

[BAX, PDPK1, PRKD2]
[APP, GCG, SIRT2]
[CXCL12, GCG, PRKD2]
[H3C14, H3C15, SET]
[DOT1L, H3C14, H3C15]
[H3C14, H3C15, KDM2B]
[H3C14, H3C15, KDM2B]
[H3C14, H3C15, PDPK1, RHOA]
[DOT1L, H3C14, H3C15]
[DOT1L, H3C14, H3C15]
[DOT1L, H3C14, H3C15]
[H3C14, H3C15, KDM2B]
[H3C14, H3C15, SIRT6]
[H3C14, H3C15, SIRT6]
[H3C14, H3C15, SIRT6]
[APP, H3C14, H3C15, IAPP, SIAH1]
[BSG, CHRFAM7A, CHRNA7, NKX6-1]
[APP, CHRFAM7A, CHRNA7]
[APP, CHRFAM7A, CHRNA7, IAPP]
[APP, CHRFAM7A, CHRNA7]
[APP, CHRFAM7A, CHRNA7]
[APP, CHRFAM7A, LY6E, PATE4]
[APP, BCL2L2, CHRFAM7A, CHRNA7]
[APP, CHRFAM7A, CHRNA7, IAPP]
[BEST1, CHRFAM7A, CHRNA7, GLRA1, SGK1, SLC12A7]
[APP, CHRFAM7A, CHRNA7]
[CHRFAM7A, CHRNA7, ILRUN]
[APP, CHRFAM7A, CHRNA7]
[APP, CHRFAM7A, CHRNA7]
[APP, CHRFAM7A, CHRNA7]
[BEST1, CHRFAM7A, CHRNA7, GLRA1, SGK1, SLC12A7]
[CHRFAM7A, CHRNA7, ILRUN]
[ADORA1, APP, CHRFAM7A, CHRNA7, GLRA1]
[ADORA1, APP, CHRFAM7A, CHRNA7]
[APP, CHRFAM7A, CHRNA7]
[CHRFAM7A, CHRNA7, GLRA1, LY6E, PATE4]
[APP, CHRFAM7A, CHRNA7]
[BEST1, CHRFAM7A, CHRNA7, GLRA1, SGK1, SLC12A7]
[BEST1, CHRFAM7A, CHRNA7, GLRA1, SGK1]
[CHRFAM7A, CHRNA7, LY6E, PATE4]
[BEST1, CHRFAM7A, CHRNA7, GLRA1, SGK1, SLC12A7]
[CHRFAM7A, CHRNA7, GLRA1]
[ADORA1, APP, CHRFAM7A, CHRNA7]
[CHRFAM7A, CHRNA7, LY6E, PATE4]
[APP, CHRFAM7A, CHRNA7]
[BEST1, CHRFAM7A, CHRNA7, GLRA1, SGK1]
[CHRFAM7A, CHRNA7, SGK1]
[CHRFAM7A, CHRNA7, GLRA1]
[CHRFAM7A, CHRNA7, GLRA1]

MEM259]
